# Identifying Population Segments by Differing Levels of COVID-19 Vaccine Confidence and Evaluating Subsequent Uptake of COVID-19 Prevention Behaviors: Web-Based, Longitudinal, Probability-Based Panel Survey

**DOI:** 10.2196/56044

**Published:** 2024-09-10

**Authors:** Joseph Luchman, Morgane Bennett, Elissa Kranzler, Rugile Tuskeviciute, Ronald Vega, Benjamin Denison, Sarah Trigger, Tyler Nighbor, Monica Vines, Leah Hoffman

**Affiliations:** 1 Fors Marsh Arlington, VA United States; 2 Department of Health and Human Services Office of the Assistant Secretary for Public Affairs Washington, DC United States

**Keywords:** COVID-19, COVID-19 vaccination, vaccine, United States, segmentation, latent class cluster analysis, vaccines, vaccination, segmentation analysis, estimation, validation, attitude, attitudes, belief, beliefs, behavior, behaviors, sociodemographic, nonintender, nonintenders, waiter, waiters, confident, confidents, social distancing, bivariate, regression analysis, survey, respondent, respondents

## Abstract

**Background:**

The COVID-19 pandemic prompted the launch of the US Department of Health and Human Services’ COVID-19 Public Education Campaign to boost vaccine confidence and uptake among adults, as vaccines are key to preventing severe illness and death.

**Objective:**

Past segmentation research relevant to COVID-19 behavior has found important differences in attitudes, sociodemographics, and subsequent COVID-19 prevention behaviors across population segments. This study extends prior work by incorporating a more comprehensive set of attitudes, behaviors, and sociodemographic variables to identify population segments by differing levels of COVID-19 vaccine confidence and evaluate differences in their subsequent uptake of COVID-19 prevention behaviors.

**Methods:**

Data were obtained from 5 waves (January 2021 to June 2022) of a web-based longitudinal, probability-based panel survey of US adults (N=4398) administered in English and in Spanish. Participants were recruited from NORC at the University of Chicago’s national AmeriSpeak panel and were invited to participate across multiple waves. Latent class cluster analysis estimated segments of respondents based on over 40 COVID-19 attitudes, beliefs, behaviors, and sociodemographics as reported in wave 1. Survey-weighted cross-tabulations and bivariate regression analyses assessed differences in COVID-19 vaccine uptake, booster uptake, mask use, and social distancing in all segments across all 5 survey waves.

**Results:**

A total of 6 segments (hardline nonintenders, prevention-compliant nonintenders, burned-out waiters, anxious waiters, skeptical confidents, and ready confidents) were identified, which differed by their COVID-19 vaccine confidence, prevention-related attitudes and behaviors, and sociodemographics. Cross-tabulations and regression results indicated significant segment membership differences in COVID-19 vaccine and booster timing, mask use, and social distancing. Results from survey-weighted cross-tabulations comparing COVID-19 vaccine and booster uptake across segments indicate statistically significant differences in these outcomes across the 6 segments (*P*<.001). Results were statistically significant for each segment (*P*<.01 for booster uptake among burned-out waiters; *P*<.001 for all other coefficients), indicating that, on average, respondents in segments with lower intentions to vaccinate reported later receipt of COVID-19 vaccines and boosters relative to the timing of vaccine and booster uptake among ready confidents.

**Conclusions:**

Results extend previous research by showing that initial beliefs and behaviors relevant to COVID-19 vaccination, mask use, and social distancing are important for understanding differences in subsequent compliance with recommended COVID-19 prevention measures. Specifically, we found that across respondent segments, the probability of vaccine and booster uptake corresponded with both COVID-19 vaccine confidence and mask use and social distancing compliance; more compliant segments were more likely to get vaccinated or boosted than less compliant segments given similar levels of vaccine confidence. These findings help identify appropriate audiences for campaigns. Results highlight the use of a comprehensive list of attitudes, behaviors, and other individual-level characteristics that can serve as a basis for future segmentation efforts relevant to COVID-19 and other infectious diseases.

## Introduction

### Overview

As of January 2023, there have been over 100 million COVID-19 cases and over 1 million deaths in the United States [1]. Vaccination against COVID-19 is the most effective way to prevent severe illness, hospitalization, and death [2]. In early 2021, prior to the widespread availability of COVID-19 vaccines in the United States, the US Department of Health and Human Services (HHS) began quantitative research in support of a national HHS COVID-19 Public Education Campaign (the Campaign “We Can Do This”) to promote greater COVID-19 vaccine confidence and uptake among US adults. This research aimed to examine which beliefs, behaviors, and sociodemographic characteristics correspond with COVID-19 vaccine confidence among US adults, with a broader goal of identifying segments or subpopulations of the adult population that could benefit from messaging about vaccination.

Research has shown that COVID-19 vaccine hesitancy is highly complex and is influenced not only by the perceived risks and benefits of receiving a vaccine, but also by such factors as mistrust in health care, policy makers, and the pharmaceutical industry; perceived risk and severity of SARS-CoV-2 (the virus that causes COVID-19) infection; access to personally trusted sources for COVID-19 information; social norms related to preventive behaviors; and sociodemographic characteristics [3-8]. Market segmentation is a valuable tool for conducting formative research during the development of public health education messaging because it allows practitioners to identify key segments within a population who may particularly benefit from the messages (see [9,10] for a similar perspective). In the absence of published segmentation research relevant to COVID-19 vaccine-hesitancy prior to the campaign launch, we undertook formative market segmentation research to provide important insights for understanding the audience (ie, adults in the United States).

In this paper, we discuss the methodologies used to develop and subsequently evaluate a market segmentation approach to identify segments of the US adult population by their levels of COVID-19 vaccine confidence. Further, we discuss the conceptual value provided by the results from the development and validation of this market segmentation approach in the context of the broader market segmentation literature.

### Extant Market Segmentation Research on COVID-19 Attitudes, Beliefs, and Behaviors

Market segmentation is a tool commonly applied to understand the attitudes, beliefs, and behaviors of homogenous subpopulations [9], which facilitates the development and placement of messages. Additionally, many attitudes, beliefs, and behaviors tend to cluster within individuals to produce profiles, which are influenced by shared structural and societal conditions, as well as social norms within their subpopulation (eg, [11]). As such, a segmentation approach to assess vaccine hesitancy provides the opportunity to better understand the heterogeneity in vaccine attitudes, beliefs, and behaviors that may occur within individuals, and helps to identify homogenous groups of individuals who share certain characteristics and may be priority populations for tailored interventions.

This type of segmentation has recently been applied to better understand COVID-19 vaccine uptake, with results-producing segments that reflect high, moderate, and low levels of confidence in COVID-19 vaccination. For example, Schneider et al [12] used segmentation to identify COVID-19 vaccine hesitancy segments with data collected in November 2020 from US adults. Results indicated a 4-segment model, with 2 vaccine-confident segments (provaccine and development concerns), 1 vaccine-hesitant segment (anti-vaccine), and a vaccine-ambivalent segment (unsure or hesitant). A key finding from the study by Schneider et al [12] was that concerns over the speed of vaccine development played a key role in differentiating those who were in the provaccine segment from those who were in the development concerns segment.

Another segmentation analysis was conducted by Wagner et al [13] using data collected from April to May 2020 among US adults aged 55 years and older. The authors produced similar results to those found by Schneider et al [12], identifying a 3-segment model with vaccine confident (vaccine acceptors), vaccine ambivalent, and vaccine-hesitant (vaccine rejectors) segments. Wagner et al [13] also validated their segmentation results with data on COVID-19 prevention behaviors among study respondents, which were collected at a 1-year follow-up. The authors found that members of the vaccine-confident segment were more likely than those in other segments to have been vaccinated against COVID-19 and to have engaged in other COVID-19 prevention behaviors at follow-up.

Chen and Shiu [11] conducted a segmentation analysis with data from the US Census Bureau’s Household Pulse Survey, collected from April 2020 to March 2021, in a sample of adults who were at least moderately hesitant toward COVID-19 vaccines. Their analysis produced a 5-segment model based on respondents’ reasons for vaccine hesitancy or refusal (eg, concerns about vaccine side effects, efficacy, and cost and lack of trust in government). A total of 3 segments were characterized by nonspecific reasons, such as endorsing all reasons (general skepticism), few reasons (not quite sure), or a single nondescript reason (just wait and see). The other 2 segments were more clearly characterized as groups that reported institutional mistrust (science and government mistrust) and safety (safety and hesitancy).

Finally, a segmentation analysis led by Lee et al [14] with data collected from US adults between April and December 2020 resulted in a 6-segment model. Consistent with the aforementioned research, results from Lee et al [14] can be arranged into higher-level groupings that represent COVID-19 vaccine confident (affluent receptives and vigilant enthusiasts), vaccine ambivalent (affluent compliants and worried willings), and vaccine-hesitant (vulnerable hesitants and skeptical reluctants) segments. Segments within the vaccine-confident groupings tended to differ according to variations in financial concerns and perceived vulnerability to COVID-19. Lee et al [14] also conducted a follow-up analysis in May 2021 to evaluate the effect of segment membership on the subsequent likelihood of being vaccinated against COVID-19. As with Wagner et al [13], the authors found that individuals in the more vaccine-confident segments were more likely to have been vaccinated against COVID-19 at follow-up compared with members of vaccine-hesitant segments.

### Extending COVID-19 Market Segmentation Research

Missing from existing research is the inclusion of beliefs, intentions, and behaviors relevant to COVID-19 prevention other than vaccination, such as mask use and social distancing, which could be important given their role in COVID-19 prevention in the earlier stages of the pandemic. This research adds to the existing literature in several ways. This research extends prior segmentation models by incorporating a more comprehensive set of attitudes, beliefs, and behaviors relevant to COVID-19 and COVID-19 prevention, as well as sociodemographic variables, compared with other segmentation models. This is an important addition to segmentation research since the nature and interpretation of the segments identified through such models depend on the factors with which they are estimated. Hence, by including a more robust set of attitudinal, behavioral, and sociodemographic variables relevant to COVID-19, this approach can reveal segments of a population that might not have otherwise been identified because of limitations in the breadth of factors included. This study offers a more nuanced, comprehensive set of segments that can inform continued COVID-19 response efforts and future work relevant to the prevention of infectious disease transmission.

Furthermore, our study derives its data from a longitudinal panel survey that began collecting data in January 2021—prior to the widespread availability of COVID-19 vaccines starting in April 2021—and for which data collection continued every 4 months through early July 2022 (5 waves). Our data are unique in the context of segmentation research relevant to COVID-19 vaccine hesitancy because they support a strong scientific inference that validates the segmentation model. Compared with analyses conducted with cross-sectional data, for which inference is limited by the contemporaneous measurement of variables, the longitudinal nature of the data allows us to examine whether segment membership, as determined with data collected during the first wave of the survey, is associated with the timing of subsequent first-dose COVID-19 vaccination and COVID-19 booster uptake, as well as engagement in other COVID-19 prevention behaviors (eg, mask use and social distancing). This combination of metrics is a novel contribution to the COVID-19 segmentation literature, and to segmentation literature more broadly, because it provides information on both the uptake and discontinued practice of several different COVID-19 prevention behaviors, organizes them by segment, and tracks them over a follow-up period of more than 1 year.

## Methods

### Data

Data came from the longitudinal COVID-19 Attitudes and Beliefs Survey (CABS). Survey respondents were recruited through NORC at the University of Chicago’s national, probability-sampling–based AmeriSpeak research panel [15] and completed 5 surveys, fielded approximately every 4 months between January 2021 and July 2022. Wave-to-wave retention rates ranged from 90% to 94%, with the greatest attrition between wave 1 and wave 2. The survey measured COVID-19 vaccination; attitudes, beliefs, and behaviors relevant to COVID-19; trust in science and experts; sociodemographic characteristics; and other items. The analytic sample for this study included 4398 respondents who completed the wave 1 survey, with data collected between January and February 2021. Data from wave 2 through wave 5 were used for validation analysis. Data were weighted to be nationally representative and to account for unequal sampling and survey nonresponse in all waves after the first. See Section S1.1 in Multimedia Appendix 1 for additional details on survey weights.

Respondent demographics as reported in wave 1 are provided in Table S1 in Multimedia Appendix 1 [15-29]. Approximately 1 in 2 (49%) respondents were younger than 45 years of age, and just over 1 in 2 (52%) were female. A total of 3 in 5 (62%) were non-Hispanic White, with fewer Hispanic or Latino (1/5, 20%), and non-Hispanic Black (1/10, 11%) respondents. Most respondents reported some college (4/9, 44%) or having received a bachelor’s degree or higher (1/3, 35%), and nearly half of the respondents (4/9, 44%) reported an annual income of less than US $50,000. Respondents were evenly distributed across liberal (3/10, 30%), moderate (4/11, 36%), and conservative (1/3, 34%) political ideologies. A plurality of respondents (4/9, 45%) reported living in a large metropolitan area, and nearly 3 in 5 (57%) respondents reported being employed. One-quarter of the respondents (1/4, 26%) indicated that they lived with 1 or more essential workers, and most respondents (4/5, 83%) reported having a preexisting health condition. See Section S1.1 in Multimedia Appendix 1 for more details about demographic variables.

### Segmentation Measures

A total of 30 numeric and 13 categorical variables (43 variables in total) were used in the multivariate segmentation model. These variables reflected a range of attitudinal, behavioral, psychological, and sociodemographic factors relevant to COVID-19 preventive behavior. Values for each of these variables were derived from CABS wave 1.

A total of 11 variables assessed respondents’ beliefs and intentions relevant to COVID-19 vaccination—reported likelihood of receiving a COVID-19 vaccine when available, the benefits and importance of getting a COVID-19 vaccine, perceived social norms related to COVID-19 vaccination, ease of and personal agency over getting a COVID-19 vaccine, the likelihood of getting a COVID-19 vaccine, the likelihood of getting a COVID-19 vaccine at a doctor’s appointment, intention to get a COVID-19 vaccine, perceived risks of COVID-19 vaccination, general vaccination decision-making by benefits and risks, and general vaccine safety and effectiveness. A total of 11 variables assessed respondents’ beliefs, intentions, and behaviors related to mask use and social distancing, with a focus on 5 main variables—the frequency with which respondents reported engaging in these behaviors, ease and agency over performing both behaviors, intentions to continue performing both behaviors, corresponding social norms, and intentions to attend gatherings with 10 or more people. A total of 7 variables assessed general views about the COVID-19 pandemic—belief in misinformation, indifference to and anxiety related to COVID-19, information burnout, effectiveness of recommended COVID-19 prevention behaviors, hopefulness that the United States will get the COVID-19 pandemic under control, and whether respondents knew people who had been hospitalized for or had died from the COVID-19 pandemic. In total, 5 variables assessed broader psychological and physiological states or beliefs, including mental distress, preexisting health conditions, perceptions and receipt of other vaccines in the past (eg, influenza [flu] vaccine), and trust in science and experts. Finally, 9 sociodemographic variables measured age, gender, race and ethnicity, household income, rurality, political ideology, education, employment status, and essential workers in the household.

### Validation Measures

Four variables reflecting COVID-19 prevention behaviors, which were obtained from all 5 waves of CABS, were used to validate the segmentation model. Two variables were the dates on which each respondent reported having received their first dose of a COVID-19 vaccine (if applicable) and their first dose of a COVID-19 booster (if applicable). The other 2 variables indexed the frequency with which respondents reported the use of masks and social distancing.

### Analysis

We incorporated all 43 segmentation variables into a multivariate latent class cluster analysis (LCCA), a clustering method that assigns respondents to mutually exclusive groups based on their patterns of responses to key metrics. Each variable in the LCCA was modeled using different distribution types and link functions. The multi-item scales and 5-point Likert-type items were modeled using a normal or Gaussian distribution with an identity link and with a mean value that was allowed to vary across classes. Variables modeled using a normal distribution were required to have the same variance across classes. All other variables were modeled as a binomial distribution with either a logit link (for binary variables) or as a proportional odds logit link (for multicategory variables) with cut points or threshold values that were allowed to vary across classes.

The analysis was conducted in Stata (version 16.1; StataCorp) using the gsem command. Cluster starting values were obtained using the default factor analysis–based approach. A series of models extracted from 2 to 8 clusters from the data were used as candidate latent class solutions. In selecting a final set of latent class results, we used a combination of fit statistics extracted from the model, as well as descriptive statistics evaluating differences between the latent classes extracted and the conceptual value added from each additional cluster extracted from the data. Missing data were accommodated by the LCCA’s native expectation maximization approach. Full details on model selection and rates of missingness by variable are reported in Section S2 in Multimedia Appendix 1.

To validate the segmentation model, we conducted several analyses that used each respondent’s predicted likelihood of segment membership as a grouping variable for reported results. Specifically, we assessed the use of this segmentation model in predicting differences in subsequent uptake of COVID-19 prevention behaviors across segments. First, 2 survey-weighted cross-tabulations were produced to compare segments by their proportions of vaccinated and boosted respondents as reported by wave 5, using weighted Pearson chi-square tests. Then 2 bivariate survey-weighted ordinary least squares regressions measuring the relationships between segment membership and the timing of (1) vaccine uptake and (2) booster uptake were estimated. Finally, these results were further illustrated substantively by estimating COVID-19 vaccine and booster uptake trajectories using the Kaplan-Meier event history methodology. Mask use and social distancing trajectories were also obtained as mean values of both variables across all 5 waves of the CABS. Full details of variable recoding and the segmentation methodology are discussed in Section S2 in Multimedia Appendix 1.

### Ethical Considerations

This study was approved by the Biomedical Research Alliance of New York (protocol 20-077-821). After eligibility was determined via screener responses, eligible individuals read the consent language and those who consented were invited to participate in the web-based survey. While completing the survey, respondents were given the opportunity to skip items. Although this study posed minimal risk, the survey included links to mental health resources for respondents to access if they experienced any distress from participating in the study. Respondents who decided to participate were offered US $10 in the first wave of the CABS and US $18 for each subsequent wave of the survey. To protect privacy and confidentiality, data collection was conducted via a secure, password-protected site. The analytic data set was stored without personally identifiable information behind tightly controlled firewalls with password-protected access for trained researchers.

## Results

### LCCA Results

We considered LCCA models with segment numbers increasing from 2 to 8. We found the 6-segment model optimal, as it produced segments that were conceptually distinct from one another, easily interpretable, and actionable. See Section S2 in Multimedia Appendix 1 for results of all LCCA models tested, and Section S3 in Multimedia Appendix 1 for details about our rationale for model selection.

Tables 1 and 2 present descriptive statistics for each of the 6 selected segments. Table 1 presents results for all 30 numeric variables. To facilitate interpretation, each of the numeric variables was standardized to have a mean of 0 (SD 1). Table 2 presents the proportions from the 13 categorical variables for each segment and for the overall sample.

**Table 1:** Standardized means of numeric segmentation variables by segment.

Variable^a^		Nonintenders		Waiters		Confidents	
		Hardline (n=279)	Prevention-compliant (n=314)	Burned-out (n=688)	Anxious (n=580)	Skeptical (n=1010)	Ready (n=1527)
**COVID-19 vaccination**							
	Benefits of COVID-19 vaccination^b^	–1.8	–1.4	–0.5	–0.4	0.3	0.9
	COVID-19 vaccine importance^b^	–1.9	–1.5	–0.7	–0.4	0.4	0.9
	COVID-19 vaccine concerns and risks^b^	1.3	1.3	0.5	0.7	–0.2	–1.0
	Normative beliefs about COVID-19 vaccination^b^	–1.7	–1.3	–0.7	–0.5	0.3	1.0
	Ease of getting a COVID-19 vaccine	–0.3	–0.4	–0.3	–0.2	0.1	0.3
	Personal agency over COVID-19 vaccination	0.2	0.2	–0.5	–0.0	–0.1	0.2
	Likelihood of getting a COVID-19 vaccine^c^	–1.5	–1.4	–0.4	–0.5	0.5	0.7
	Likelihood of getting a COVID-19 vaccine at a doctor’s appointment	–1.7	–1.6	–0.5	–0.5	0.4	0.9
	Intention to get a COVID-19 vaccine	–1.7	–1.6	–0.5	–0.5	0.5	0.9
**Mask use**							
	Ease of using a mask	–1.4	–0.2	–0.8	0.3	0.0	0.5
	Personal agency over mask use	0.3	–0.1	–0.1	0.1	–0.0	0.0
	Normative beliefs about mask use^b^	–2.0	0.1	–1.1	0.4	0.0	0.7
	Frequency of mask use^d^	–2.2	0.2	–0.7	0.4	0.2	0.4
	Intention to wear a mask	–1.8	0.2	–0.9	0.4	0.2	0.5
**Social distancing**							
	Ease of social distancing	–0.6	0.1	–0.6	0.3	–0.0	0.3
	Personal agency over social distancing	0.1	–0.1	–0.3	0.2	0.1	0.0
	Normative beliefs about social distancing^b^	–1.8	0.2	–1.1	0.4	–0.0	0.7
	Frequency of social distancing^d^	–1.4	–0.0	–0.7	0.3	0.1	0.4
	Number of gatherings attended with 10 or more people^e^	0.6	0.0	0.5	–0.1	–0.1	–0.3
	Intention to practice social distancing	–1.7	0.1	–0.9	0.3	0.1	0.6
**Other beliefs relevant to COVID-19**							
	COVID-19 misinformation^b^	1.6	0.8	0.9	–0.0	–0.0	–0.9
	COVID-19 information burnout^b^	1.3	0.6	0.4	–0.1	0.1	–0.6
	COVID-19 indifference^b^	1.7	0.5	0.9	–0.2	0.1	–0.9
	Effectiveness of recommended COVID-19 prevention behaviors^b^	–2.2	–0.7	–0.9	0.1	0.3	0.8
	Hopefulness that the United States will get COVID-19 under control^f^	–0.5	–0.3	–0.2	–0.2	0.1	0.3
**General vaccination beliefs**							
	General vaccination decision-making informed by benefits and risks	–0.2	–0.0	–0.6	0.0	–0.0	0.3
	General vaccine safety and effectiveness^b^	–1.6	–1.2	–0.7	–0.5	0.1	1.0
**Psychological characteristics**							
	General psychological distress^b^	–0.3	0.2	0.1	0.1	–0.1	–0.0
	COVID-19 anxiety^b^	–1.3	–0.3	–0.4	0.2	0.0	0.4
**Institutional trust**							
	Trust in science and experts^b^	–1.5	–0.9	–0.7	–0.2	–0.0	0.9
Percentage of sample, n (%)		279 (6)	314 (7)	688 (16)	580 (13)	1010 (23)	1527 (35)

^a^Unless noted, items were measured using a 5-point scale: strongly disagree, disagree, neither agree nor disagree, agree, and strongly agree.

^b^Indicates a scale comprising more than 1 individual item. All items that comprise the scale are detailed in Section S1 in Multimedia Appendix 1.

^c^Item measured using a 5-point scale: very unlikely, somewhat unlikely, neither likely nor unlikely, somewhat likely, and very likely.

^d^Item measured using a 5-point scale: never, rarely, sometimes, very often, and always.

^e^Item measured using a 6-point scale, from 0 times to 5 or more times.

^f^Item measured using a 5-point scale: not hopeful at all, hardly hopeful, somewhat hopeful, hopeful, and very hopeful.

**Table 2:** Percentages of categorical segmentation variables by segment^a,b^.

		Nonintenders, %			Waiters, %			Confidents, %	
		Hardline (n=279)	Prevention-compliant (n=314)	Burned-out (n=688)		Anxious (n=580)	Skeptical (n=1010)		Ready (n=1527)
**How soon one will get a COVID-19 vaccine**									
	Will vaccinate as soon as they can	1	0	16		5	77		94
	Will wait to get vaccinated for 1 or more reasons	27	37	70		88	23		6
	Will never get vaccinated	72	63	15		7	0		0
**Age (years)**									
	18-24	6	21	20		10	10		8
	25-44	45	40	43		43	28		29
	45-64	35	30	28		35	35		31
	65 and older	14	9	9		13	26		33
**Sex**									
	Male	51	32	54		37	54		49
	Female	49	68	46		63	46		51
**Race or ethnicity**									
	Non-Hispanic White	83	65	57		41	67		67
	Non-Hispanic Black	3	16	13		28	9		8
	Hispanic or Latino	11	13	22		22	16		14
	Other race or ethnicity	3	6	8		9	9		11
**Education**									
	No college	48	52	50		47	34		25
	Some college	31	30	28		29	30		23
	Bachelor’s degree or higher	21	18	22		24	35		52
**Income (US $)**									
	Less than 50,000	42	56	56		64	41		35
	50,000 to less than 75,000	21	18	21		13	23		20
	75,000 to less than 100,000	22	8	9		10	12		15
	100,000 and greater	14	18	14		13	23		31
**Political ideology**									
	Liberal	3	15	9		24	22		50
	Moderate	25	40	40		49	37		34
	Conservative	73	45	51		27	41		16
**Rurality**									
	Large metropolitan area	27	33	34		51	45		48
	Small metropolitan area	39	47	43		36	37		38
	Non-metropolitan area	34	20	23		13	18		15
**Employment status**									
	Employed	60	60	61		55	51		50
	Unemployed	6	9	15		10	8		6
	Retired	15	9	10		13	27		31
	Not in labor force	19	22	15		22	14		13
**Essential worker in household**									
	Yes	29	32	26		28	21		21
	No	71	68	75		72	79		79
**Preexisting health condition**									
	Yes	78	82	81		82	86		84
	No	22	18	19		18	14		16
**Received a flu shot this season or last season**									
	No flu shot this season or last	79	73	47		54	23		12
	Flu shot this season or last	21	27	53		46	77		88
**Knows someone who was hospitalized for or died from COVID-19**									
	Yes	54	53	53		50	44		45
	No	46	47	47		50	56		55
Percentage of sample		6	7	16		13	23		35

^a^The variable proportions across all respondents can be found in Section S1 in Multimedia Appendix 1.

^b^We have opted not to report the absolute n values for each cell as we believe reporting these values will result in increased difficulty in comparing the segments’ results by category.

### Segment Results

#### Overview

The 6 segments were categorized into 3 broader groups based on their overall levels of COVID-19 vaccine confidence. The 3 broader groups included nonintenders, who were broadly opposed to getting a COVID-19 vaccine; waiters, who intended to wait to get vaccinated against COVID-19; and confidents, who intended to get a COVID-19 vaccine as soon as they were able to. The segments are ordered from most to least vaccine hesitant.

#### Hardline Nonintenders

The first segment (1/16, 6% of the population), the hardline nonintenders, was the least willing to engage in any form of COVID-19 protective behaviors compared with the other segments. Hardline nonintenders reported consistently negative attitudes, beliefs, and behaviors related to COVID-19 vaccines, as well as to other COVID-19 preventive behaviors. Compared with the other segments, respondents in this segment also reported the most skepticism of scientists and experts, the strongest beliefs in COVID-19 misinformation, and the greatest indifference to COVID-19 risks (Table 1). Hardline nonintenders had the strongest tendency among all segments to report no intention to get a COVID-19 vaccine and were the least inclined to report having received a flu shot in recent flu seasons. Additionally, this segment had the highest concentration of respondents from a non-metro area, identified as very politically conservative, and largely identified as non-Hispanic White (Table 2).

#### Prevention-Compliant Nonintenders

The next segment (1/15, 7% of the population), the prevention-compliant nonintenders, reported low intentions to get a COVID-19 vaccine but differed from the hardline nonintenders with respect to their reported mask use, social distancing, and sociodemographic profile. Compared to the hardline nonintenders, this segment had similar COVID-19 vaccine intentions and attitudes, skepticism about science, indifference to the pandemic, and beliefs in COVID-19 misinformation. However, prevention-compliant nonintenders reported greater intentions to engage in and frequently participate in mask use and social distancing and reported the highest level of general psychological distress compared with all other segments. Prevention-compliant nonintenders also reported a somewhat greater willingness to be vaccinated against COVID-19 compared with their hardline nonintender counterparts. Overall, prevention-compliant nonintenders were younger, more female, had lower incomes, were more racially and ethnically diverse, and were less politically conservative than hardline nonintenders.

#### Burned-Out Waiters

The third segment (2/13, 16% of the population), the burned-out waiters, comprised respondents who were more open to COVID-19 vaccination than either of the nonintender segments, with a plurality of respondents reporting that they would wait to receive a COVID-19 vaccine. Although this segment reported more favorable beliefs and intentions surrounding COVID-19 vaccination than the nonintenders, burned-out waiters reported generally unfavorable attitudes and behaviors relevant to mask use and social distancing. This segment also reported greater COVID-19 indifference, misinformation, and information burnout compared with the anxious waiters. Burned-out waiters tended to be younger, more male than female, and employed more often in contrast with anxious waiters. This segment was also more diverse in terms of race and ethnicity compared with the nonintender segments, leaned politically conservative, and tended to live in non-metro or smaller metro areas.

#### Anxious Waiters

The fourth segment (2/15, 13% of the population), the anxious waiters, were both more favorable toward COVID-19 vaccination than the nonintender segments and were more favorable toward mask use and social distancing than their burned-out waiter counterparts. Anxious waiters also reported higher levels of COVID-19 anxiety and greater concerns about COVID-19 vaccines compared with burned-out waiters and were comparatively less indifferent about the pandemic and more trusting of science. Anxious waiters tended to be female, have a lower income, most racially and ethnically diverse segment, and are most inclined to live in a large metro area. Additionally, compared with the burned-out waiters, the anxious waiters were older and less politically conservative.

#### Skeptical Confidents

The fifth segment (2/9, 23% of the population), the skeptical confidents, were generally favorable toward COVID-19 vaccination and other COVID-19 prevention behaviors, with most of them reporting that they intended to get a COVID-19 vaccine as soon as possible. However, skeptical confidents viewed mask use and social distancing less favorably than they viewed COVID-19 vaccination. Moreover, this segment reported notably greater levels of COVID-19 indifference and information burnout compared with the ready confidents and the anxious waiters. As shown in Table 2, skeptical confidents, in general, reported higher levels of education and higher incomes than most other segments. Skeptical confidents tended to be slightly more male than female and were more politically conservative than the ready confidents.

#### Ready Confidents

Respondents in the sixth and final segment (4/11, 35% of the population), the ready confidents, reported that they intended to get a COVID-19 vaccine as soon as possible. Ready confidents reported the most pro–COVID-19 prevention beliefs, attitudes, and behaviors of any segment, and reported the highest rates of flu shot receipt in previous years. Table 2 also shows that ready confidents tended to be the oldest adults, have the highest levels of education, have the highest incomes, and be the most politically liberal of all the segments.

### Validation Results

#### Overview

Results from bivariate survey-weighted cross-tabulations comparing COVID-19 vaccine and booster uptake across segments indicate statistically significant differences in these outcomes across the 6 segments (*P*<.001; see Tables S15 and S16 in Multimedia Appendix 1). Table S17 in Multimedia Appendix 1 illustrates the bivariate relationship between segment membership and the timing of the COVID-19 vaccine and booster uptake, with the ready confident segment as the reference category; coefficients reflect the relationship between the timing of each behavior and segment membership relative to the ready confidents. Results were statistically significant for each segment (*P*<.01 for booster uptake among burned-out waiters; *P*<.001 for all other coefficients), indicating that, on average, respondents in segments with lower intentions to vaccinate reported later receipt of COVID-19 vaccines and boosters relative to the timing of vaccine and booster uptake among ready confidents. Additional multivariate regressions assessing the relationships between segment membership and the frequency of mask use (Table S18 in Multimedia Appendix 1) and social distancing (Table S19 in Multimedia Appendix 1) are also included in Multimedia Appendix 1.

**Figure 1:**
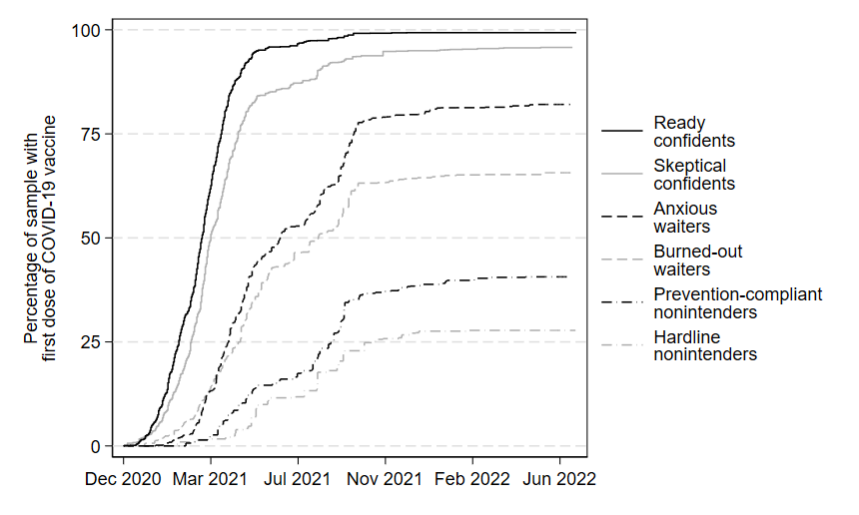
Trajectory of COVID-19 vaccine uptake over time by segment, December 2020-July 2022. COVID-19 vaccines became widely available to all adults in the United States in April 2021. See Tables S20-S25 in Multimedia Appendix 1 for survival tables for each segment.

**Figure 2:**
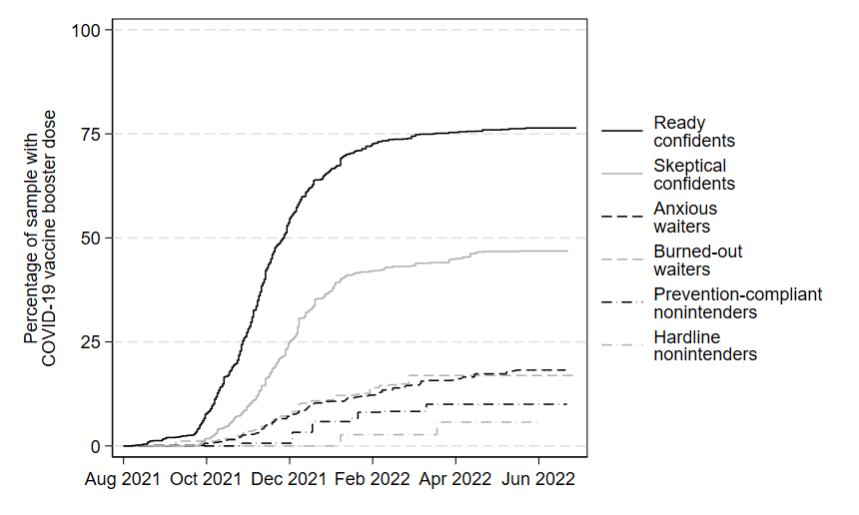
The trajectory of COVID-19 booster uptake over time by segment, August 2021-July 2022. COVID-19 boosters became widely available to all adults in the United States in November 2021. See Tables S26-S31 in Multimedia Appendix 1 for survival tables for each segment.

**Figure 3:**
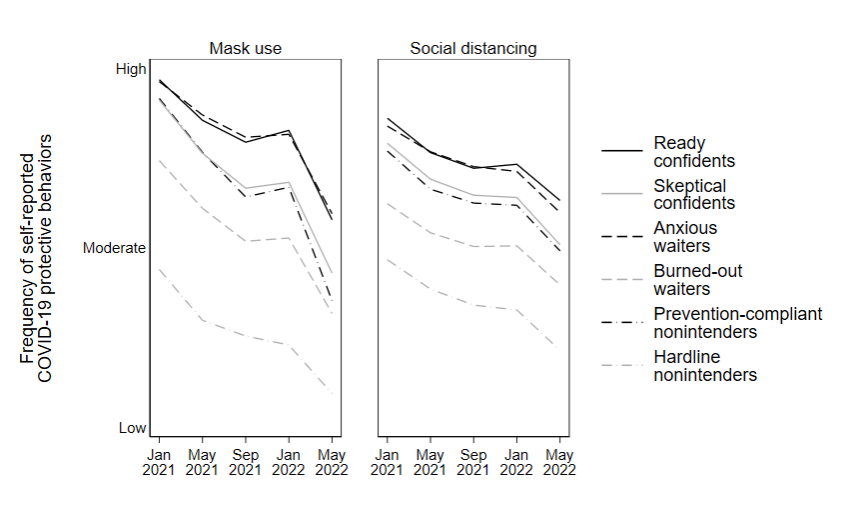
Trajectory of COVID-19 prevention behaviors over time by segment, January 2021-May 2022. Survey dates in this figure are based on the average survey completion date for each of the 5 COVID-19 Attitudes and Beliefs Survey waves between January 2021 and May 2022. Each line depicts the average frequency of mask use or social distancing by segment. Items assessing the frequency of mask use and social distancing were measured on 5-point Likert-type scales and are described in greater detail in Multimedia Appendix 1. The x-axis scales have been altered for a more concise display of the frequency scale.

To illustrate these significant differences more concretely, the trajectories of COVID-19 behaviors are charted. Figure 1 depicts the rates of COVID-19 vaccine uptake among respondents within each of the 6 segments between December 2020 and July 2022. Figure 2 depicts the rates of COVID-19 booster uptake among respondents within each of the 6 segments between August 2021 and July 2022. Figure 3 presents the self-reported frequency of respondent mask use and social distancing across all 5 waves of the CABS, with each wave represented by the fielding month during which most respondents completed the corresponding survey.

#### Hardline Nonintenders

In wave 1, the hardline nonintenders tended to report, more than any other segment, that they would never get a COVID-19 vaccine. This intention was supported by the validation results presented in Figure 1, which show that this segment had the slowest and lowest rate of vaccine uptake of all segments; by July 2022, hardline nonintenders reported only 25% (1/4) were vaccinated. Similarly, Figure 2 shows that, among hardline nonintenders who were vaccinated, this segment had the slowest uptake and lowest end rates of COVID-19 booster uptake, with less than 10% (1/10) of vaccinated segment members reporting that they had received a booster shot. Finally, Figure 3 shows that hardline nonintenders consistently reported the lowest frequency of mask use and social distancing across all 5 waves.

#### Prevention-Compliant Nonintenders

The prevention-compliant nonintenders reported intentions to never receive a COVID-19 vaccine but were willing to engage in other COVID-19 prevention behaviors. This willingness to engage in mask use and social distancing was consistent across survey waves and remained fairly high until early 2022. In fact, the reported frequency of mask use and social distancing for this segment mirrored that of the skeptical confidents segment. Despite a greater frequency of mask use and social distancing, prevention-compliant nonintenders were slow to get COVID-19 vaccines and had the second lowest rate of uptake overall, at about 40% (2/5; Figure 1). Similarly, COVID-19 booster uptake within this segment was low, and only about 15% (1/6) of vaccinated respondents reported receiving a booster shot (Figure 2).

#### Burned-Out Waiters

The burned-out waiters were likely to wait to get a COVID-19 vaccine, were relatively indifferent to COVID-19 risks, and reported COVID-19 information burnout. The results in Figure 1 show that the majority (65%, 2/3) of respondents within this segment were vaccinated by July 2022. In fact, by August 2021, 50% (1/2) of the burned-out waiters had received a COVID-19 vaccine, indicating a much faster rate of vaccine uptake compared with the rates of vaccine uptake for the 2 nonintender segments. Conversely, the rate of COVID-19 booster uptake among vaccinated burned-out waiters was only slightly greater than that same rate among nonintender groups, with approximately 20% (1/5) of all vaccinated burned-out waiter respondents reporting booster uptake. Burned-out waiters also reported the second-lowest frequencies of mask use and social distancing across all waves (Figure 3).

#### Anxious Waiters

The overwhelming majority of anxious waiters reported that they would wait to receive a COVID-19 vaccine and, on average, reported relatively high levels of general COVID-19 anxiety. Similar to the burned-out waiters, most anxious waiters were ultimately vaccinated against COVID-19; 50% (1/2) of respondents in this segment were vaccinated by June 2021 and nearly 80% (4/5) were vaccinated by July 2022 (Figure 1). COVID-19 booster uptake among vaccinated anxious waiters mirrored that of burned-out waiters, with approximately 20% (1/5) of all vaccinated anxious waiter respondents reporting booster uptake by July 2022 (Figure 2). According to Figure 3, frequencies of mask use and social distancing among anxious waiters were nearly identical to those reported by the ready confidents.

#### Skeptical Confidents

Skeptical confidents were characterized by their strong intentions to get vaccinated against COVID-19, paired with their relatively less-favorable beliefs about other COVID-19 preventive behaviors and their somewhat skeptical views of the risks of the pandemic. As shown in Figure 1, these strong vaccination intentions resulted in substantial vaccine uptake for this segment; skeptical confidents achieved a nearly 100% (1/1) vaccination rate, a rate far greater than what was achieved by the anxious waiters. However, their rate of vaccine uptake was slower compared with the ready confidents. The differences between skeptical confidents and ready confidents are more apparent in Figures 2 and 3. Skeptical confidents reported relatively high COVID-19 booster uptake (nearly 50% of, or 1 in 2, vaccinated respondents). However, this rate was lower than and was achieved more slowly in comparison to booster uptake among ready confidents. Skeptical confidents also reported notably lower frequencies of mask use and social distancing than ready confidents—the frequency with which they reported these behaviors were more closely aligned with prevention-compliant nonintenders.

#### Ready Confidents

Ready confidents were characterized by their overwhelming readiness to receive a COVID-19 vaccine, which effectively corresponded with a 100% (1/1) vaccination rate and the fastest trajectory of vaccine uptake of the segments (Figure 1). Similarly, ready confidents reported the highest rate of COVID-19 booster uptake, with nearly 75% (3/4) of vaccinated respondents within the segment having received a booster (Figure 2), and the highest frequencies of mask use and social distancing among the segments (Figure 3).

## Discussion

### Principal Findings

In this paper, we described the characteristics of 6 distinct segments of adults in the United States according to their beliefs, intentions, and behaviors relevant to COVID-19 prevention as reported in January-February 2021. We also validated the segmentation approach and described, by segment, the trajectory of their COVID-19 prevention behaviors from the widespread availability of initial vaccines (April 2021) to initial booster availability (September 2021) through the end of the study period (July 2022). The 6 segments were categorized into 3 broader groups according to their overall levels of intention to receive a COVID-19 vaccine—nonintenders, who were broadly opposed to getting a COVID-19 vaccine; waiters, who intended to wait to get a COVID-19 vaccine for 1 or more reasons; and confidents, who intended to get a COVID-19 vaccine as soon as they were able to. Taken together, results from this study suggest that the segmentation of an adult population according to their COVID-19 beliefs, intentions, and behaviors can facilitate a nuanced understanding of different perspectives on COVID-19 and predict subsequent compliance with recommended behaviors to prevent SARS-CoV-2 infection and transmission.

Consistent with other segmentation research relevant to COVID-19 vaccination, our results show a strong convergence between initial beliefs, intentions, and behaviors relevant to the COVID-19 pandemic and subsequent COVID-19 preventive behaviors across segments. In particular, our validation findings among COVID-19 vaccine confidence segments are consistent with those demonstrated by Wagner et al [13]. Thus, our research provides additional support for the segmentation of populations, using COVID-19 vaccination attitudes and behaviors, into broader groupings ordered by their levels of vaccine confidence (see also [9,12] for similar findings).

Importantly, our research extends the work by Wagner et al [13] through the inclusion of other COVID-19 prevention behaviors (specifically, mask use and social distancing) in the development of our model, which produced a larger and more nuanced set of segments. Our results indicate that such distinctions across COVID-19 vaccine confidence segments have important implications for predicting subsequent mask use and social distancing behavior, but that they also influence the likelihood and rates of subsequent COVID-19 vaccination and booster uptake. Consider, for example, the difference between the ready confident and skeptical confident segments. Nearly 100% of respondents in both groups received a COVID-19 vaccine by July 2022. If we were to evaluate these groups based solely on their rates of vaccine uptake, both groups would appear to be nearly identical. However, when considering COVID-19 booster uptake, the booster uptake rate among skeptical confidents was lower than the rate among ready confidents. These 2 segments also demonstrated distinct differences in their frequencies of mask use and social distancing over time. Our findings suggest that when aiming to capture a comprehensive set of segments within a market, segmentation research may benefit from incorporating a similarly comprehensive range of beliefs, intentions, and behaviors into segmentation models [30].

### Implications

Within each of the 6 segments, the patterns of responses to segmenting variables provide insights that may facilitate the development of targeted messaging for other COVID-19 preventive behaviors, such as the uptake of bivalent COVID-19 vaccines that became available in September 2022. For example, compared with the burned-out waiters, the anxious waiters reported greater anxiety about the COVID-19 disease and some concerns about the safety of COVID-19 vaccines, yet respondents in this segment also reported moderate trust of scientists and experts and did not generally endorse COVID-19 misinformation. Since many anxious waiters ultimately received a COVID-19 vaccine, efforts to bolster this segment’s compliance with recommended preventive behaviors (eg, getting a bivalent or updated COVID-19 vaccine) could include messaging and messaging strategies that integrate such segment-specific information—such as messages that emphasize the safety of the original and bivalent COVID-19 vaccines—delivered by trusted messengers such as scientists or other experts. Similarly, although the majority of prevention-compliant nonintenders did not receive a COVID-19 vaccine, many of the respondents in this segment continued to engage in mask use and social distancing throughout the study period. Efforts to increase bivalent COVID-19 vaccine uptake in this segment could include, for example, messaging that highlights the overlap between the prevention behaviors in which they consistently engaged and the behavior of interest. However, the use of such messaging approaches should be appropriately vetted and tested prior to implementation.

### Limitations and Future Directions

The segmentation model was developed for the purpose of campaign outreach, such that the approach taken by the research team to select variables for inclusion in the LCCA model—as well as the methodology used to select a final set of segments—was guided by practical needs, interpretability, and methodological rigor. Several variables that have guided similar segmentation approaches in past research, such as concerns about finances and isolation anxiety, were omitted from our model. Additionally, we did not include beliefs about COVID-19 boosters in the segmentation model; these were not collected in wave 1 of the survey because they had not yet been developed. This omission of variables may partially explain the differences between the segments identified in this study and the segments produced by other segmentation work in the context of COVID-19 vaccination. In a departure from other segmentation research, we also included variables relevant to mask use and social distancing, which may partially account for these distinctions. Such differences may also be explained by data collection periods, because the data used to develop our segmentation model were collected from January to February 2021, which was later than the data collection periods for all other segmentation models reviewed to date. However, as described earlier, there was considerable overlap between the segments in our model and those identified through similar segmentation research, despite differences in the variables included and the data collection periods. Given the timing of the data collection for our segmentation model and the population from which our survey sample was drawn, the results from this study may not be broadly applicable to COVID-19 prevention behaviors for other periods of the COVID-19 pandemic, nor to other populations of interest.

The criteria for selection of the 6-segment model on which we report included the interpretability of the segments and their use for audience identification. These criteria differ from the criteria for other published segmentation research in which researchers have tended to rely only on fit statistics and other tests as the basis for selecting the appropriate number of segments (eg, [12]). When considering model fit, we found that the inclusion of more than 6 segments did not improve the interpretability or targeting potential of the overall segmentation solution, despite providing a better fit to the data. Thus, the 6-segment model was the best fit for our needs, though we acknowledge that the inclusion of additional segments could have allowed us to differentiate between the segments more clearly with respect to the validation variables (eg, COVID-19 booster uptake).

The data collected in this study did not facilitate the evaluation of how customized messages elicit different COVID-19 prevention behavior outcomes by segment. In fact, to our knowledge, there are no published evaluations that demonstrate an association between exposure to qualitatively different COVID-19 messages tailored to suit the needs of different segments (as identified through a segmentation analysis) and COVID-19 prevention behaviors. Future research could fill this gap by assessing the extent to which targeted messages elicit different attitudinal and behavioral outcomes relevant to COVID-19 prevention across segments. Inherent in segmentation research is the assumption that differentiation between segments indicates the reasons for engaging in, or abstaining from, the behaviors on which they have been segmented. Confirmatory evidence demonstrating that messages that are customized to segment needs elicit differential effects on behavior by segment would help justify the use of segmentation for the purposes of understanding a population and facilitating audience outreach. Finally, validation analyses do not account for the likely influence of interventions (eg, public education campaigns) and environmental factors (eg, mandates and other policies) that occurred during the CABS survey fielding on respondents’ COVID-19 prevention behaviors; accounting for such influence is beyond the scope of this work.

### Conclusions

This study found that across segments of US adults, the timing of the COVID-19 vaccine and booster uptake and the uptake of other COVID-19 prevention behaviors corresponded with adults’ initial COVID-19 vaccination intentions. Study findings reveal the importance of examining a wide range of cognitions, sociodemographics, and other variables to identify which segments of a population are more or less likely to get vaccinated. As the threat of the COVID-19 pandemic continues to evolve over time, these findings can be used to inform COVID-19 response by public health organizations. This research also serves as a conceptual basis for future segmentation modeling to inform the response to yet-unknown infectious diseases, especially those diseases for which several preventive behaviors are recommended. The results from this and other relevant segmentation research can help researchers, practitioners, and policy makers identify and prioritize population segments for intervention according to the intended outcomes of interest. In so doing, this research can support the rapid development of effective outreach strategies for future public health crises to speed the uptake of preventive behaviors, decrease the severity of illness, and save lives.

## Appendix

Multimedia Appendix 1 Cluster analysis and validation.

## Abbreviations

CABS: COVID-19 Attitudes and Beliefs Survey
HHS: US Department of Health and Human Services
LCCA: latent class cluster analysis
