# Supplementary material for: Identifying Population Segments by Differing Levels of COVID-19 Vaccine Confidence and Evaluating Subsequent Uptake of COVID-19 Prevention Behaviors: Web-Based, Longitudinal, Probability-Based Panel Survey
Source: JMIR Public Health Surveill. 2024 Sep 10;10:e56044. doi: 10.2196/56044 (PMC11422724; doi:10.2196/56044)
Supplement: Multimedia Appendix 1 [file publichealth_v10i1e56044_app1.docx]

**S1. Methods**

*S1.1 Data*

All survey data used for the analysis were obtained from the first five waves of the COVID-19 Attitudes and Beliefs Survey (CABS). The CABS is a nationally representative, probability-sampled longitudinal survey of U.S. adults administered every 4 months starting in January 2021. CABS respondents were recruited from NORC at the University of Chicago’s AmeriSpeak panel.^1^ The CABS averages about 35 minutes to complete, is in a web-only format, and is administered in both English and Spanish. The content of the CABS focuses on respondent adherence to COVID-19 preventive behaviors, including COVID-19 vaccination and boosters; attitudes and beliefs relevant to COVID-19 and preventive behaviors; views on science, research, and medical professionals; recalled Campaign exposure; and sociodemographic characteristics [15].

A total of 4,398 respondents completed the Wave 1 survey (fielded from January through February 2021). Of those respondents, 3,962 completed the Wave 2 survey (fielded from May through June 2021), 3,642 completed the Wave 3 survey (fielded from September through November 2021), 3,398 completed the Wave 4 survey (fielded from January through March 2022), and 3,182 completed the Wave 5 survey (fielded from May to July 2022). The segmentation modeling was conducted on the Wave 1 data. The data from Wave 2 though Wave 5 were used for validation analysis. Table S1 provides respondent demographic information as reported in Wave 1.

*Table S1: Respondent Demographics as Reported in Wave 1 (January–February 2021)*

|  | Percentage | Sample Size |
| --- | --- | --- |
| Age |  |  |
| 18–24 | 6.0% | 264 |
| 25–44 | 43.3% | 1,906 |
| 45–64 | 29.3% | 1,287 |
| 65 and older | 21.4% | 941 |
| Gender |  |  |
| Male | 48.5% | 2,110 |
| Female | 51.5% | 2,244 |
| Race/Ethnicity |  |  |
| Non-Hispanic White | 61.6% | 2,708 |
| Non-Hispanic Black | 10.7% | 470 |
| Hispanic/Latino | 20.3% | 892 |
| Other race/ethnicity | 7.5% | 328 |
| Education |  |  |
| No college | 21.1% | 929 |
| Some college | 44.2% | 1,945 |
| Bachelor's degree or higher | 34.7% | 1,524 |
| Income |  |  |
| Less than $50,000 | 44.2% | 1,861 |
| $50,000 to less than $75,000 | 20.7% | 870 |
| $75,000 to less than $100,000 | 13.2% | 555 |
| $100,000 and greater | 21.9% | 922 |
| Political Ideology |  |  |
| Liberal | 30.0% | 1,309 |
| Moderate | 36.4% | 1,589 |
| Conservative | 33.6% | 1,467 |
| Rurality |  |  |
| Large metropolitan area | 45.2% | 1,989 |
| Small metropolitan area | 38.1% | 1,674 |
| Non-metropolitan area | 16.7% | 735 |
| Employment Status |  |  |
| Employed | 57.3% | 2,504 |
| Unemployed | 8.3% | 363 |
| Retired | 20.9% | 915 |
| Not in labor force | 13.5% | 588 |
| Essential Worker in Household |  |  |
| Yes | 26.1% | 1,143 |
| No | 73.9% | 3,244 |
| Pre-Existing Health Condition |  |  |
| Yes | 82.7% | 3,039 |
| No | 17.3% | 636 |

The CABS is a complex sampled survey that used sampling strata defined by age, race/ethnicity, education, and gender; the combinations of each resulted in 48 sampling strata that remained the same for all five waves. Survey weights were developed for each survey wave because the unweighted AmeriSpeak panel did not precisely represent the overall U.S. population with respect to its demographic distribution. As such, the survey weights developed for each wave were based on a series of adjustments that accounted for unequal sampling of the longitudinal study panel from the AmeriSpeak panel frame, as well as unknown eligibility and nonresponse from eligible households. Unequal sampling accounts for differences between the AmeriSpeak panel composition and the overall U.S. population, while also accounting for minimal oversampling of select subgroups (e.g., by age and race/ethnicity) in Wave 1 to ensure that retention targets for each subgroup were maintained over time.

The AmeriSpeak panel was sampled by household, not by person; and the household weight, developed specifically for this longitudinal study panel, was calibrated using the U.S. Census Bureau’s Current Population Survey (CPS) by sampling stratum to reflect the number of households per stratum. Subsequent adjustments to the survey weights accommodated person-level attributes, including nonresponse of adults within a household as well as raking calibration adjustments by age, gender, race/ethnicity, age by gender, age by race/ethnicity, U.S. Census Division, education, household tenure, and household phone status (i.e., cell phone, landline). An additional adjustment was applied to the survey weights to accommodate nonresponse across Wave 2 through Wave 5 to adjust for bias resulting from panel attrition. Starting in Wave 2, another adjustment to the survey weights was applied to correct for known COVID-19 vaccine uptake rates by geographic area, as provided by data from the Centers for Disease Control and Prevention (CDC). Starting in Wave 4, another adjustment to the survey weights was applied to correct for known COVID-19 booster uptake rates by geographic area, as provided by data from the CDC.

*S1.2 Measures Used in Segmentation*

The authors selected 43 variables for inclusion in the segmentation model, several of which were based on the results of psychometric analysis to produce multi-item scales. Nearly all of the attitude, belief, and opinion questions available on the CABS were used in the process of generating the multi-item scales. Most question sets were obtained from existing measures as is noted in the description of each scale. In cases where items may not have derived from a scale, a small team of subject matter experts grouped the items into an existing scale. The sections below detail the source, the questions selected for inclusion, and key psychometric results. Factor analyses were estimated using principal axis factoring and all had an eigenvalue of greater than 1. Note that all questions below were asked on the CABS unless noted as being obtained from the CABS survey vendor. Reverse-coded items are flagged with an asterisk. All questions, except where indicated, were responded to on a 5-point Likert-type scale: Strongly disagree; Disagree; Neither agree nor disagree; Agree; and Strongly agree.

*S1.2.1 COVID-19 Vaccine Importance*. Following research of both Pogue et al. [16], as well as Reiter, Pennel, and Katz [17], we developed a three-item scale to measure the perceived importance of COVID-19 vaccines. This scale showed high internal consistency ($\alpha_{Cronbach}$ = .95). The item wording and factor loadings are included in Table S2. Respondents were asked the extent to which they agreed with each prompt.

*Table S2*. Psychometric Results for COVID-19 Vaccine Importance Scale

| Survey Item | Factor Loading |
| --- | --- |
| It is important for me to get all of the recommended COVID-19 vaccines. | 0.93 |
| It is important for everyone to get all of the recommended COVID-19 vaccines. | 0.93 |
| Getting all of the recommended vaccines helps reduce the spread of COVID-19. | 0.89 |

*S1.2.2 COVID-19 Misinformation.* We assessed respondents’ beliefs in COVID-19 misinformation following research by Hornik et al. [18] creating a five-item scale. The retained five-item scale showed relatively high internal consistency ($\alpha_{Cronbach}$ = .80). The item wording and factor loadings are included in Table S3. Respondents were asked the extent to which they agreed with each prompt.

*Table S3*. Psychometric Results for COVID-19 Misinformation Scale

| Survey Item | Factor Loading |
| --- | --- |
| COVID-19 is probably a hoax. | 0.71 |
| Public health officials are exaggerating the seriousness of COVID-19. | 0.82 |
| There are people who want the pandemic to continue for their own personal gain. | 0.54 |
| COVID-19 was created in a lab. | 0.61 |
| COVID-19 is more dangerous than the flu.* | 0.66 |

*Note: Reverse-coded items are flagged with an asterisk.*

*S1.2.3 Effectiveness of Recommended COVID-19 Prevention Behaviors.* We assessed respondents’ attitudes about COVID-19 prevention measures’ effectiveness with three items developed from similar questions reported in the Understanding America Study [19]. The three items showed good internal consistency ($\alpha_{Cronbach}$ = .86). The item wording and factor loadings are included in Table S4. Respondents were asked the extent to which they agree or disagree that each of the following actions are effective at keeping them safe from COVID-19.

*Table S4*. Psychometric Results for Effectiveness of Recommended COVID-19 Prevention Behaviors Scale

| Survey Item | Factor Loading |
| --- | --- |
| Wearing a face mask. | 0.89 |
| Maintaining a social distance. | 0.88 |
| Receiving a COVID-19 vaccine. | 0.67 |

*S1.2.4 COVID-19 Vaccine Concerns and Risks.* Five items assessed respondents’ extent of worry about side effects of vaccination, as well as beliefs about COVID-19 vaccine health complications. These five items were informed by the work of Pogue et al. [16] and had good internal consistency ($\alpha_{Cronbach}$ = .87). The item wording and factor loadings are included in Table S5. Respondents were asked the extent to which they agreed with each prompt.

*Table S5*. Psychometric Results for COVID-19 Vaccine Concerns and Risks Scale

| Survey Item | Factor Loading |
| --- | --- |
| I am worried that a COVID-19 vaccine could give me COVID-19. | 0.70 |
| I would rather build immunity by exposure to an infected individual than receive a COVID-19 vaccine. | 0.71 |
| I am worried about side effects of a COVID-19 vaccine for myself. | 0.7 |
| I am worried that side effects of a COVID-19 vaccine could be worse than COVID-19 itself. | 0.87 |
| The benefits of a COVID-19 vaccine outweigh any risks associated with it.* | 0.74 |

*Note: Reverse-coded items are flagged with an asterisk.*

*S1.2.5 Benefits of COVID-19 Vaccination.* Five items were developed using content from Reiter, Pennel, and Katz [17] to assess respondents’ beliefs about the economic and personal benefits of receiving a COVID-19 vaccine. The five items showed evidence of high internal consistency ($\alpha_{Cronbach}$ = .93). The item wording and factor loadings are included in Table S6. Respondents were asked the extent to which they agreed with each prompt.

*Table S6*. Psychometric Results for Benefits of COVID-19 Vaccination Scale

| Survey Item | Factor Loading |
| --- | --- |
| I would get a COVID-19 vaccine if it would help life return to normal more quickly. | 0.75 |
| A COVID-19 vaccine will allow me to spend more time with my loved ones. | 0.89 |
| A COVID-19 vaccine will allow me to return to normal day-to-day activities. | 0.87 |
| A COVID-19 vaccine will improve the economy. | 0.85 |
| A COVID-19 vaccine will allow schools and businesses to reopen. | 0.89 |

*S1.2.6 General Vaccine Safety and Effectiveness.* We assessed respondents’ beliefs about the safety and efficacy of vaccines generally (not just COVID-19 vaccines) with seven items generated using survey content in Kwok et al. [20]. The six-item scale showed evidence of good internal consistency ($\alpha_{Cronbach}$ = .86). The item wording and factor loadings are included in Table S7. Respondents were asked the extent to which they agreed with each prompt.

*Table S7*. Psychometric Results for General Vaccine Safety and Effectiveness Scale

| Survey Item | Factor Loading |
| --- | --- |
| I am completely confident that vaccines are safe. | 0.80 |
| Vaccinations are effective. | 0.80 |
| I am confident that public authorities decide to approve vaccines when it is in the best interest of the community. | 0.72 |
| Diseases that have a vaccine (e.g., shingles, pneumonia) are not so bad that I need to get a vaccine for them.* | 0.51 |
| When everyone is vaccinated, I do not have to get vaccinated too.* | 0.66 |
| I get vaccinated because it protects people with a weaker immune system. | 0.73 |

*Note: Reverse-coded items are flagged with an asterisk.*

*S1.2.7. COVID-19 Indifference.* We assessed respondents’ beliefs regarding the seriousness of the COVID-19 pandemic using four items obtained from Howard [21]. These four items produced a single factor (loadings range = .71–.89) and showed rather high internal consistency ($\alpha_{Cronbach}$ = .90). The item wording and factor loadings are included in Table S8. Respondents were asked the extent to which they agreed with each prompt.

*Table S8*. Psychometric Results for COVID-19 Indifference Scale

| Survey Item | Factor Loading |
| --- | --- |
| Concerns regarding COVID-19 are overblown. | 0.89 |
| There is currently too much panic around COVID-19. | 0.81 |
| COVID-19 is not as dangerous as the media claims it to be. | 0.88 |
| People should not be worried about COVID-19. | 0.71 |

*S1.2.8 Normative Beliefs about COVID-19 Vaccination, Mask Use, and Social Distancing.* Nine items were developed based on the research of Knotek II et al. [22] to assess respondents’ perception of other’s beliefs that the respondent would practice prevention behavior to control the spread of COVID-19. We developed three three-item scales, each pertaining to social distancing, mask use, and COVID-19 vaccination. Each three-item scale showed good internal consistency ($\alpha_{Cronbach}$vaccinaton = .88; $\alpha_{Cronbach}$mask use = .88; $\alpha_{Cronbach}$social distancing = .86). The item wording and factor loadings are included in Table S9 by scale behavior. Respondents were asked the extent to which they agreed with each prompt.

*Table S9*. Psychometric Results for Normative Belief about COVID-19 Vaccination, Mask Use, and Social Distancing Scales

| Survey Item | Factor Loading |
| --- | --- |
| Normative Beliefs about COVID-19 Vaccination | |
| People who are important to me will receive a COVID-19 vaccine when it is available. | 0.83 |
| People who are important to me believe that I should receive a COVID-19 vaccine when it is available. | 0.87 |
| Getting all of the recommended vaccines is the right thing to do. | 0.78 |
| Normative Beliefs about Masking | |
| People who are important to me wear a face mask when they are out in public. | 0.81 |
| People who are important to me believe that I should wear a face mask when I am out in public. | 0.87 |
| Wearing a face mask is the right thing to do. | 0.78 |
| Normative Beliefs about Social Distancing | |
| People who are important to me maintain a social distance from individuals outside of their household. | 0.77 |
| People who are important to me believe that I should maintain a social distance from individuals outside of my household. | 0.86 |
| Social distancing is the right thing to do. | 0.77 |

*S1.2.9 COVID-19 Information Burnout.* We derived four items from related research by So and Popova [23] to assess respondents’ attitudes regarding hearing too much about the COVID-19 pandemic. The four items showed acceptable internal consistency ($\alpha_{Cronbach}$ = .79). The item wording and factor loadings are included in Table S10. Respondents were asked the extent to which they agreed with each prompt.

*Table S10*. Psychometric Results for COVID-19 Information Burnout Scale

| Survey Item | Factor Loading |
| --- | --- |
| I have heard enough about how important it is to follow the COVID-19 health guidelines. | 0.66 |
| After hearing them for months, messages about COVID-19 seem repetitive. | 0.77 |
| I am burned out from hearing that COVID-19 is a serious problem. | 0.80 |
| I want more information regarding COVID-19.* | 0.50 |

*Note: Reverse-coded items are flagged with an asterisk.*

*S1.2.10 Trust in Science and Experts.* We assessed respondents’ beliefs regarding the trustworthiness of scientists, experts, and public health organizations using thirteen items developed from research published by Nadelson et al. [24]. The thirteen items had high internal consistency ($\alpha_{Cronbach}$ = .94). The item wording and factor loadings are included in Table S11. Respondents were asked the extent to which they agreed with each prompt.

*Table S11*. Psychometric Results for Trust in Science and Experts Scale

| Survey Item | Factor Loading |
| --- | --- |
| When scientists change their minds about a scientific idea, it diminishes my trust in their work.* | 0.65 |
| Scientists ignore evidence that contradicts their work.* | 0.78 |
| We can trust scientists to share their discoveries even if they do not like their findings. | 0.71 |
| We should trust the work of scientists. | 0.78 |
| We cannot trust scientists because they are biased in their perspectives.* | 0.79 |
| Today’s scientists will sacrifice the well-being of others to advance their research. | 0.70 |
| I trust the information I receive from government public health experts. | 0.81 |
| Government public health experts have their own agenda.* | 0.74 |
| Government public health experts have my best interests in mind. | 0.79 |
| Information provided by government public health experts changes too often for me.* | 0.71 |
| Information provided by governmental public health experts has been helpful to me in the past. | 0.72 |
| I have been misled by government public health experts in the past.* | 0.66 |
| How often, if ever, do you think governmental public health organizations (HHS, CDC, FDA, etc.) get the facts right about the COVID-19 outbreak? | 0.69 |

*Note: Reverse-coded items are flagged with an asterisk.*

*S1.2.11 General Psychological Distress.* We included the Psychological Health Questionnaire version-4 [25], along with an additional item related to general mental health, to assess respondents’ frequency of experiencing different negative emotions and mood states. The five items showed good internal consistency ($\alpha_{Cronbach}$ = .89). The item wording and factor loadings are included in Table S12. Respondents were asked the extent to which they agreed with each prompt.

*Table S12*. Psychometric Results for General Psychological Distress Scale

| Survey Item | Factor Loading |
| --- | --- |
| Feeling nervous, anxious, or on edge | 0.84 |
| Not being able to stop or control worrying | 0.84 |
| Feeling down, depressed, or hopeless | 0.87 |
| Little interest or pleasure in doing things | 0.83 |
| In general, how would you rate your mental or emotional health?* | 0.60 |

*Note: Reverse-coded items are flagged with an asterisk.*

*S1.2.12 COVID-19 Anxiety.* Guided by Ahorsu et al.’s [26] COVID-19 anxiety scale, we developed four items to assess respondents’ experiences of anxiety for different reasons, and specifically owing to the COVID-19 pandemic. All four items showed good internal consistency ($\alpha_{Cronbach}$ = .86). The item wording and factor loadings are included in Table S13. Respondents were asked the extent to which they agreed with each prompt.

*Table S13*. Psychometric Results for COVID-19 Anxiety Scale

| Survey Item | Factor Loading |
| --- | --- |
| I worry a lot about COVID-19. | 0.85 |
| I am afraid of dying because of COVID-19. | 0.77 |
| I am afraid of losing my family members because of COVID-19. | 0.76 |
| When watching news and stories about COVID-19 on social media, I become nervous or anxious. | 0.69 |

Several questions were included individually in the segmentation analysis instead of as a multi-item scale. These items are discussed below.

*S1.2.12. Likelihood of Getting a COVID-19 Vaccine.* Respondents were asked to answer, “What is the likelihood that you will get a COVID-19 vaccine?” on a 5-point scale from “Very unlikely” to “Very likely.”

*S1.2.13. How Soon One Will Get a COVID-19 Vaccine.* Respondents were asked to answer, “A Food and Drug Administration (FDA)-authorized vaccine to prevent COVID-19 is now available at no cost. How soon will you get vaccinated? For this question, assume there is enough vaccine so that everyone who wants it can get it.” Respondents could report that they: “Will get a vaccine as soon as they can,” “Will wait to get vaccinated for one or more reasons,” or “Will never get vaccinated.”

*S1.2.14 Personal Agency Over COVID-19 Vaccination.* Respondents were asked to answer, “In the next year, it is mostly up to me whether I get a COVID-19 vaccine or not” on a 5-point scale from “Strongly agree” to “Strongly disagree.”

*S1.2.15 Ease of Getting a COVID-19 Vaccine.* Respondents were asked to answer, “In the next year, it would be easy for me to get a COVID-19 vaccine” on a 5-point scale from “Strongly agree” to “Strongly disagree.”

*S1.2.16 Intention to Get a COVID-19 Vaccine.* Respondents were asked to answer, “In the next year, I intend to get a COVID-19 vaccine” on a 5-point scale from “Strongly agree” to “Strongly disagree.”

*S1.2.17 Personal Agency Over Mask Use.* Respondents were asked to answer, “It is mostly up to me whether I wear a face mask or not” on a 5-point scale from “Strongly agree” to “Strongly disagree.”

*S1.2.18 Ease of Using a Mask.* Respondents were asked to answer, “It is easy for me to wear a face mask” on a 5-point scale from “Strongly agree” to “Strongly disagree.”

*S1.2.19 Intention to Wear a Mask* Respondents were asked to answer*,* “In the next week, I intend to wear a face mask” on a 5-point scale from “Strongly agree” to “Strongly disagree.”

*S1.2.20 Number of Gatherings Attended with 10 or More People.* Respondents were asked to answer, “In the last 7 days, how many times were you in a room with a group of more than 10 people? Exclude your household and your visits to the supermarket or pharmacy to get essential food or medicine” on a 6-point scale from “0 times” to “5 or more times.”

*S1.2.21 Personal Agency Over Social Distancing.* Respondents were asked to answer, “It is mostly up to me whether I maintain social distancing or not” on a 5-point scale from “Strongly agree” to “Strongly disagree.”

*S1.2.22 Ease of Social Distancing.* Respondents were asked to answer, “It is easy for me to maintain social distancing” on a 5-point scale from “Strongly agree” to “Strongly disagree.”

*S1.2.23 Intention to Practice Social Distancing.* Respondents were asked to answer, “In the next week, I intend to practice social distancing” on a 5-point scale from “Strongly agree” to “Strongly disagree.”

*S1.2.24 Likelihood of Getting a COVID-19 Vaccine at Doctor’s Appointment.* Respondents were asked to answer, “I would accept a COVID-19 vaccine offered during a regularly scheduled appointment with my health care provider” on a 5-point scale from “Strongly agree” to “Strongly disagree.”

*S1.2.25 General Vaccination Decision-Making Informed by Benefits and Risks.* Respondents were asked to answer, “When I think about getting vaccinated, I weigh the benefits and risks to make the best decision possible” on a 5-point scale from “Strongly agree” to “Strongly disagree.”

*S1.2.26 Hopefulness That United States Will Get COVID-19 Under Control.* Respondents were asked to answer, “How hopeful are you that the U.S. will get COVID-19 under control in the next 6 months?” on a 5-point scale from “Very hopeful” to “Not hopeful at all.”

*S1.2.27 Age*. The four age categories were constructed from an integer-valued age variable obtained from the survey vendor. These categories included “18–24,” “25–44,” “45–64,” and “65 and older.”

*S1.2.28 Gender.* Respondent gender identity was based on two questions obtained from the survey vendor. The first question respondents were asked to answer about the sex they were assigned at birth on their original birth certificate. The second question respondents were asked to answer about how they describe their gender identity. Respondents were classified as “male” if they were assigned at birth as and continue to identify as male. Respondents were classified as “female” if they were assigned at birth as and continue to identify as female. Respondents with other patterns of responses were classified as “gender minority.” Several observations were missing data on one or both variables and were classified as missing.

*S1.2.29 Race/Ethnicity.* Racial/ethnic identity was constructed from two frame variables obtained from the survey vendor. The first was called “combined race/ethnicity” and categorized all respondents into the following identities: “White, non-Hispanic;” “Black, non-Hispanic;” “Other, non-Hispanic;” “Hispanic;” “2+, non-Hispanic;” and “Asian, non-Hispanic.” The other was categorized by: “White Only,” “Black or African American Only,” “American Indian or Alaska Native,” “Asian Indian Only,” “Chinese Only,” “Filipino Only,” “Japanese Only,” “Korean Only,” “Vietnamese Only,” “Other Asian,” “Native Hawaiian Only,” “Samoan Only,” “Other Pacific Islander,” “Some other race,” and “Multirace.”

Respondents were classified as “Non-Hispanic White” if they were “White, non-Hispanic” on the first and “White Only” on the second variable. Respondents were classified as “Non-Hispanic Black” if they were “Black, non-Hispanic” on the first and “Black or African American Only” on the second variable. Respondents were classified as “Hispanic/Latino” if they were “Hispanic” on the first and were not in an “American Indian or Alaska Native” identity on the second. Respondents were classified as “Other Racial/Ethnicity” for all other combinations of the two variables. The collapsed categories in Table II in the main manuscript are, “Non-Hispanic White,” “Non-Hispanic Black,” “Hispanic/Latino,” and “Other Race/Ethnicity.”

*S1.2.30 Education*. The three education categories were recoded from a variable obtained from the survey vendor including the following categories: “Less than high school,” “High school graduate or equivalent,” “Vocational/tech school/some college/associate degree,” “Bachelor’s degree,” and “Post-grad study/professional degree.” Respondents were classified as “No college” when they were in “Less than high school” and “High school graduate or equivalent” categories. Respondents were classified as “Some college” when they were in the “Vocational/tech school/some college/associate degree” category. Respondents were classified as “Bachelor’s degree or higher” when they were in the “Bachelor’s degree” and “Post-grad study/professional degree” categories. The collapsed categories in Table II in the main manuscript are, “No college,” “Some college,” and “Bachelor’s degree or higher.”

*S1.2.31 Income.* The four-category income variable was measured with a question asking respondents, “Last year, what was your total household income from all sources, before taxes?” Respondents could endorse one of nine options: “Less than $15,000;” “$15,000 to $24,999;” “$25,000 to $34,999;” and “$35,000 to $49,999” were coded as “Less than $50,000.” “$50,000 to $74,999” was coded as “$50,000 to less than $75,000.” “$75,000 to $99,999” was coded as “$75,000 to less than $100,000.” “$100,000 to $149,999;” “$150,000 to $199,999;” and “$200,000 and over” were coded as “$100,000 and greater.” Several respondents refused to answer this question and were coded as missing. The collapsed categories in Table II in the main manuscript are: “Less than $50,000,” “$50,000 to less than $75,000,” “$75,000 to less than $100,000,” and “$100,000 and greater.”

*S1.2.32* *Political Ideology.* The three political ideology categories were generated from a variable obtained from the survey vendor where respondents indicated their political ideology. “Very liberal” and “Somewhat liberal” were coded as “Liberal.” Those who responded with “Moderate” remained “Moderate.” “Somewhat conservative” and “Very conservative” were coded as “Conservative.” Several respondents refused to answer this question and were classified as missing. A total of 865 respondents responded to an alternative ideology metric that asked only if they were “Liberal,” “Moderate,” or “Conservative,” which was used to update the original metric.

*S1.2.33 Knows Someone Who Was Hospitalized or Died From COVID-19.* Constructed by the analysis team from two survey questions, this Respondents were asked to answer: “Do you know anyone who has been hospitalized for COVID-19?” and “Of the people you know who have had COVID-19, have any of them died as a result of COVID-19?” Respondents indicated “Yes” or “No” to both. If a respondent indicated “Yes” on either question, they were coded as “Knows someone.” If a respondent indicated “No” to both, they were coded as “Does not know someone.” All other patterns were flagged as missing.

*S1.2.34 Received a Flu Shot This Season or Last Season.* Constructed by the analysis team from two survey questions, respondents were asked to answer: “For the following years, did you receive a flu vaccine?” to which a respondent could respond “Yes” or “No” to the years 2020/2021 and/or 2019/2020. If a respondent indicated receiving a flu shot in either year, they were coded as “Flu shot this season or last.” If a respondent indicted that they did not receive a shot for both years, they were coded as “No flu shot this season or last.” All other patterns were flagged as missing.

*S1.2.35 Rurality.* The three-category rurality variable was determined for each respondent using their 2013 Rural-Urban Continuum Codes (RUCC) available from the U.S. Department of Agriculture [27] linked to each respondent’s U.S. county of residence obtained from the survey vendor. The three categories reported were formed from the nine RUCCs. “Large metropolitan area” was coded as those respondents living in “counties in metro areas of 1 million population or more.” “Small metropolitan area” was coded as those respondents living in “counties in metro areas of 250,000 to 1 million population” or in “counties in metro areas of fewer than 250,000 population.” All other RUCCs were classified as “Non-metropolitan area.”

*S1.2.36 Employment Status.* This was measured using a survey question asking respondents: “Which statement best describes your current employment status?” Respondents were coded as “Employed” if they responded as “Working – as a paid employee” or “Working – self-employed.” Respondents were coded as “Unemployed” if they responded as “Not working – on temporary layoff from a job” or “Not working – looking for work.” Respondents were coded as “Retired” if they responded as “Not working – retired.” Finally, respondents were coded as “Not in labor force” if they responded as “Not working – disabled” or “Not working – other.” Respondents who refused to answer this question were coded as missing.

*S1.2.37 Essential Worker in Household.* Among respondents who endorsed working in the prior question, respondents were asked: “Does where you work (e.g., state/territory) designate your occupation as providing ‘essential’ services? ‘Essential’ may vary depending on where you live but may include those who provide:” where the respondent was given examples of specific occupations and duties commonly classified as essential workers. All respondents endorsed either “Yes” or “No.” Respondents ineligible for this question due to not working were coded as “No.”

In addition, respondents were asked, “How many people in your household, excluding yourself, work in occupations that are designated as providing ‘essential’ services?” Respondents then indicated an integer value for the number of household members that qualify. The analysis team combined these responses so that, if a respondent indicated being an essential worker or having more than 0 essential workers in their household, they were coded as “One or more essential workers.” If a respondent responded that they were not an essential worker and that there were no essential workers in their household, they coded as “No essential workers.” All other patterns were flagged as missing.

*S1.2.38* *Pre-Existing Health Condition.* Preexisting health condition status was constructed by the analysis team from a series of questions asking respondents whether they had the following conditions: hypertension, diabetes, cancer, lung disease, ever experienced a heart attack, Alzheimer’s disease, dementia, asthma, cerebrovascular disease, chronic kidney disease, sickle cell disease, liver disease, compromised immune system, and/or were overweight/obese. In addition, respondents who indicated being a smoker or pregnant were flagged as having a pre-existing condition. Respondents who reported that they do not have any of these conditions or who refused to provide a response for a given condition were categorized as not having a preexisting health condition. Respondents who refused to respond to all items assessing these conditions were coded as missing.

*S1.3 Measures used in Segmentation and Validation*

Two variables were used in both the segmentation modeling as well as for the follow-up validation analyses. These variables were available in all five waves of the CABS.

*S1.3.1 Frequency of Mask Use.* Respondents were asked to answer, “How often do you wear a mask when you are outside of your home and in public?” Respondents responded on a 5-point scale ranging from “Always” to “Never.”

*S1.3.2 Frequency of Social Distancing.* Respondents were asked to answer, “How often do you maintain a social distance of at least 6 feet from others when outside your home in public? Exclude members from your household when responding.” Respondents responded on a 5-point scale ranging from “Always” to “Never.”

*S1.4 Measures Used in Validation*

Two variables were used only in the validation analyses. The waves in which these variables were available is noted in their description.

*S1.4.1 Vaccine Uptake and Timing.* Starting in Wave 3 of the CABS, all respondents who reported receiving at least one dose of a COVID-19 vaccine were asked about the day, month, and year of their first dose. Respondents who reported being vaccinated in Wave 3 through Wave 5 were asked about their first vaccine dose date in each wave.

Only vaccine dates that passed a quality assurance check were used in the analysis. Specifically, a respondent’s vaccine date must have occurred between two CABS waves in which the respondent went from having no shots of a COVID-19 vaccine to at least one shot. For respondents who reported being vaccinated at Wave 1 of the CABS, they must have had one reported vaccine date between December 11, 2020 and the date of their response on Wave 1. December 11 was chosen because this is when the first COVID-19 vaccine was approved for Americans 16 years of age and up [28

]. A total of 159 respondents were eliminated at this stage for reporting vaccine dates that do not correspond with their reported uptake across waves. An additional 94 respondents were also eliminated because they either reported a vaccine date that was consistent with their pattern of uptake responses but changed their responses across waves to report that they were no longer vaccinated, or had interrupted data provided by the data vendor (i.e., missed a wave but remained in the panel). In addition, 756 respondents dropped out of the CABS sample prior to Wave 3 when the vaccine date questions began. Finally, 135 respondents reported being vaccinated but refused to provide or provided an incomplete vaccination date. A total of 2,659 respondents had a confirmed vaccination date. The remaining 595 respondents were confirmed unvaccinated and reported being not vaccinated in each wave in which they were a respondent.

*S1.4.2 Booster Uptake and Timing.* Starting in Wave 5 of the CABS, all respondents who reported a pattern of COVID-19 vaccine uptake that indicated receiving a booster (i.e., three shots with a two-shot series; two shots with a one-shot series) were asked about the day, month, and year of their booster dose.

As with vaccine dates, only booster dates that passed a series of quality assurance checks were used in the analysis. First, only the 2,659 respondents with a valid first-dose vaccine date in the previous steps were eligible to have a valid booster date. Booster dates had to occur after a respondent’s vaccination date and the analysis team set that a minimum of 4 months of time had to pass to allow the booster date to be deemed eligible. This eliminated 129 respondents (34 reported booster dates earlier than vaccination; 95 reported a date after vaccination but less than 4 months). Similar to the vaccine procedure, a respondent’s booster date must have occurred between two CABS waves in which the respondent went from not being boosted to one in which they were boosted. An additional 228 respondents were eliminated due to failure in reporting a date consistent with their pattern of responding. Finally, 598 respondents either dropped out of the CABS sample between the time of their vaccine date and Wave 5 (263 respondents) or indicated being boosted at Wave 5 but either refused to provide a date or provided an incomplete date (335 respondents). A total of 1,167 respondents had a confirmed booster date. The remaining 526 respondents were confirmed unboosted and reported not having been vaccinated in each wave in which they were a respondent. An additional 11 respondents were vaccinated and reported being unboosted but had a vaccination date of less than 4 months prior and were considered ineligible for boosting and were not counted among the confirmed unboosted group.

**S2. Analyses**

***S2.1 Latent Class Cluster Analysis***

All study variables except for vaccine uptake and timing were analyzed using a latent class cluster analysis (LCCA). Each variable in the LCCA was modeled using different distribution types and link functions. The multi-item scales and 5-point Likert type items were modeled using a normal/Gaussian distribution with an identity link and with a mean value that was allowed to vary across classes. Variables modeled using a normal distribution were required to have the same variance across classes. All the other variables were modeled as a binomial distribution with either a logit link (for binary variables) or as a proportional odds logit link (for multi-category variables) with cut-points or threshold values that were allowed to vary across classes.

The analysis was conducted in Stata version 16.1 using the *gsem* command. Cluster starting values were obtained using the default factor analysis-based approach. A series of models that extracted from two to eight clusters from the data were used as candidate latent class solutions. In selecting a final set of latent class results, we used a combination of fit statistics extracted from the model, as well as descriptive statistics evaluating differences between the latent classes extracted and the conceptual value added from each additional cluster extracted from the data, as is discussed below. Missing data were accommodated by the LCCA’s native expectation maximization approach. Rates of missingness, as well as sample sizes, by variable are reported below in Table S14.

*Table S14: Missing Data Rates by Segmentation Variable*

| Benefits of COVID-19 Vaccination | 1.0% | 44 |
| --- | --- | --- |
| COVID-19 Vaccine Importance | 0.3% | 13 |
| COVID-19 Vaccine Concerns and Risks | 0.9% | 40 |
| Normative Beliefs about COVID-19 Vaccination | 0.9% | 40 |
| Ease of Getting a COVID-19 Vaccine | 2.4% | 106 |
| Personal Agency Over COVID-19 Vaccination | 2.3% | 101 |
| Likelihood of Getting a COVID-19 Vaccine | 2.0% | 88 |
| Likelihood of Getting a COVID-19 Vaccine at a Doctor’s Appointment | 0.3% | 13 |
| Intention to Get a COVID-19 Vaccine | 2.3% | 101 |
| Ease of Using a Mask | 0.1% | 4 |
| Personal Agency Over Mask Use | 0.1% | 4 |
| Normative Beliefs about Mask Use | 0.8% | 35 |
| Frequency of Mask Use | 0.0% | 0 |
| Intention to Wear a Mask | 0.2% | 9 |
| Ease of Social Distancing | 0.2% | 9 |
| Personal Agency Over Social Distancing | 0.3% | 13 |
| Normative Beliefs about Social Distancing | 1.1% | 48 |
| Frequency of Social Distancing | 0.0% | 0 |
| Number of Gatherings Attended with 10 or More People | 0.3% | 13 |
| Intention to Practice Social Distancing | 0.3% | 13 |
| COVID-19 Misinformation | 1.2% | 53 |
| COVID-19 Information Burnout | 0.8% | 35 |
| COVID-19 Indifference | 0.8% | 35 |
| Effectiveness of Recommended COVID-19 Prevention Behaviors | 0.4% | 18 |
| Hopefulness That U.S. Will Get COVID-19 Under Control | 0.6% | 26 |
| General Vaccination Decision-Making Informed by Benefits and Risks | 0.3% | 13 |
| General Vaccine Safety and Effectiveness | 1.3% | 57 |
| General Psychological Distress | 1.5% | 66 |
| COVID-19 Anxiety | 1.2% | 53 |
| Trust in Science and Experts | 2.1% | 92 |
| How Soon One Will Get a COVID-19 Vaccine | 10.3% | 453 |
| Age | 0.0% | 0 |
| Gender | 1.0% | 44 |
| Race/Ethnicity | 0.0% | 0 |
| Education | 0.0% | 0 |
| Income | 4.3% | 189 |
| Political Ideology | 0.8% | 35 |
| Rurality | 0.0% | 0 |
| Employment Status | 0.6% | 26 |
| Essential Worker in Household | 0.3% | 13 |
| Pre-Existing Health Condition | 16.4% | 721 |
| Received a Flu Shot This Season or Last Season | 2.9% | 128 |
| Knows Someone Who Was Hospitalized or Died From COVID-19 | 3.2% | 141 |

***S2.2 Kaplan-Meier Failure Curves***

Once the final set of latent classes were extracted, all respondents to the CABS were classified into their most-likely latent class. The 3,337 respondents with valid dates or who were confirmed as not vaccinated were *set* as survival analysis data using December 1, 2020, as the date of initial exposure. Respondents who were not vaccinated by Wave 5 were denoted as right censored on the date of their Wave 4 response; respondents who were not vaccinated as of Wave 3 and dropped out of the CABS at Wave 3 were denoted as right censored on the date of their Wave 3 response.

The uptake trajectory, by most likely latent class membership, was estimated using smoothed Kaplan Meier Failure curves [29] over the study period.

**S3. Results**

**S3.1 Latent Class Cluster Analysis Results**

The two-segment model (AIC_2_ = 427,710; BIC_2_ = 428,610) showed substantial improvement in fit to the data relative to a model with a single latent class (AIC_1_ = 475,854; BIC_1_ = 476,397), describing all the data at once with a ~10% improvement to the information criteria with little loss in classification accuracy (entropy = .996). The substantive results from the two-segment model showed that the respondents were separated most strongly on COVID-19 prevention behavior and attitudes, with 59% of respondents forming a more compliant and 41% of respondents forming a less compliant class.

The three-segment model (AIC_3_ = 412,661; BIC_3_ = 413,920) resulted in a further ~4% decrease to the information criteria and little loss in classification accuracy (entropy = .993). The substantive results for the three-segment model showed that the more COVID-19 prevention behavior compliant class was largely reproduced with 52% of respondents falling into this class. The three-segment model’s contribution was to split the previously lower COVID-19 prevention behavior compliant class into a moderately compliant class (35%) and a low compliance class (13%).

The four-segment model (AIC_4_ = 404,650; BIC_4_ = 406,266) resulted in a further ~2% decrease to the information criteria and, again, little loss in classification accuracy (entropy = .988). The four-segment model’s substantive results showed that the moderate (27%) and low (9%) compliance groups were obtained from the data again. The change with four classes was to split the more compliant class into a very highly compliant class (37%) and a highly compliant class (27%).

At five latent classes, the fit metrics (AIC_5_ = 397,695; BIC_5_ = 399,669) continued to obtain a consistent rate of improvement to fit with a ~2% decrease to the information criteria. Classification accuracy also remained consistent with an entropy value of .988. The substantive results for the five-cluster model deviated from the mostly linear pattern observed to this point among the classes. As in the previous models, most of the classes were reproduced with the low compliance class (7%), the highly compliant class (27%) and the very highly compliant class (35%) all being reproduced. The five-cluster model extended on past results by splitting the moderately compliant group into two harder-to-order groups. The moderately compliant class had statistics that were more similar to the previous moderately compliant group (16%) in that they showed consistently moderate compliance across types of protective behaviors. The second moderately compliant class (14%) showed a differentiation across protective behaviors such that they were very compliant in terms of mask wearing and social distancing, but tended to be opposed to vaccination against COVID-19.

The six-segment model’s fit metrics (AIC_6_ = 394,295; BIC_6_ = 396,646) also continued to improve fit to the data with a ~1% decrease to the information criteria without a noteworthy decrease to classification accuracy (entropy = .986). The substantive results across the latent classes resulted in reproducing four of the latent classes from the previous model including the low compliance class (6% unweighted), the across-behavior consistent moderately compliant class (16% unweighted), the highly compliant class (23% unweighted), and very highly compliant class (35% unweighted). The six-segment model subdivided into two groups from the masking-and-distancing but not vaccination class obtained from the previous model. Specifically, the split formed a masking-and-distancing with stronger opposition to vaccination class (7% unweighted) as well as a masking-and-distancing with moderate opposition to vaccination class (13% unweighted).

Model fit metrics for the seven-segment (AIC_7_ = 391,621; BIC_7_ = 394,349) showed stable improvement—they also obtained a ~1% decrease to the information criteria with a negligible decrease to classification accuracy (entropy = .982). Continuing the trend in previous models, five of the classes from the previous model were reproduced again to form the low compliance class (6%), the masking-and-distancing with stronger opposition to vaccination class (7%), masking-and-distancing with moderate opposition to vaccination class (13%), the across-behavior consistent moderately compliant class (13%), and the very highly compliant class (30%). The seven-segment model produced a split among the previously highly compliant group into a somewhat highly compliant group (10%) and a highly compliant group (21%).

Our team did not find that the split produced by the seven-segment model, which divided up two highly compliant groups, was sufficiently useful enough to consider keeping that model, because the additional clusters had an increasingly narrowing value in terms of model fit. As such, we stopped evaluating the latent class models at the seven-segment model and retained the six-segment model as our preferred model.

**S3.2 Statistical Testing Results**

Results reported below in Tables S15, S16, and S17 are described in the main manuscript. Note that no family-wise Type I error corrections to the hypothesis tests were applied.

*Table S15: Survey-Weighted COVID-19 Vaccination Behavior by Cluster*

|  | Unvaccinated (%) | Vaccinated^a^ (%) | Chi-Square Test Results^b^ |
| --- | --- | --- | --- |
| Hardline Non-Intenders | 83.40 | 16.60 |  |
| Prevention-Compliant Non-Intenders | 64.99 | 35.01 |  |
| Burned-Out Waiters | 42.04 | 57.96 | *F* (3.56, 171.11) = 144.1548   *P <* 0.001 |
| Anxious Waiters | 23.07 | 76.93 |  |
| Skeptical Confidents | 4.21 | 95.79 |  |
| Ready Confidents | 0.85 | 99.15 |  |
| Total Population^c^ | 19.61 | 80.39 |  |

^a^ Percentages represent the proportion of respondents in the segment who received a first-dose COVID-19 vaccine.

^b^ The weighted Pearson chi-square test produced a design-adjusted F-statistic.

^c^ *N* = 3,254.

*Table S16: Survey-Weighted COVID-19 Booster Behavior by Cluster*

|  | Not Boosted (%) | Boosted^a^ (%) | Chi-Square Test Results^b^ |
| --- | --- | --- | --- |
| Hardline Non-Intenders | 86.23 | 13.77 |  |
| Prevention-Compliant Non-Intenders | 81.60 | 18.40 |  |
| Burned-Out Waiters | 69.00 | 31.00 | *F* (4.28, 205.55) = 56.357  *P <* 0.001 |
| Anxious Waiters | 62.17 | 37.83 |  |
| Skeptical Confidents | 33.63 | 66.37 |  |
| Ready Confidents | 11.29 | 88.71 |  |
| Total Population^c^ | 30.62 | 69.38 |  |

^a^ Percentages represent the proportion of respondents in the segment who received a booster dose among those in the segment who received a first-dose vaccine. The sample size is smaller because it reflects respondents who completed initial vaccination and for whom their date of first-dose vaccination was validated, as discussed in section S1.4.2.

^b^ The weighted Pearson chi-square test produced a design-adjusted F-statistic.

^c^ *N* = 1,694.

| *Table S17: Survey-Weighted Regression of Wave 1 Clusters on Vaccine/Booster Dates* | | |  |
| --- | --- | --- | --- |
|  | Vaccine Dates (Std. Err.) | Booster Dates^a^ (Std. Err.) | |
| Hardline Non-Intenders^b^ | 113.54*** | 89.24*** | |
|  | (15.53) | (25.05) | |
| Prevention-Compliant Non-Intenders | 134.88*** | 54.96*** | |
|  | (10.23) | (11.77) | |
| Burned-Out Waiters | 75.10*** | 21.86** | |
|  | (10.16) | (7.98) | |
| Anxious Waiters | 77.41*** | 42.94*** | |
|  | (4.59) | (8.03) | |
| Skeptical Confidents | 20.37*** | 17.11*** | |
|  | (3.52) | (4.23) | |
| Intercept | 22349.53*** | 22599.30*** | |
|  | (1.52) | (1.85) | |
| N | 2,736 | 1,188 | |
| R-Squared | 0.24 | 0.09 | |
|  | | |  |

*** *P* < .001, ** *P* < .01, * *P* < .05.

^a^ The r-squared value for the booster timing model was much lower than the r-squared value for the vaccine timing model, indicating that these six segments did not explain the variation in the timing of booster uptake as well as they explained the variation in the timing of vaccine uptake.

^b^ Ready Confidents serve as the reference group for this analysis. The sample size is smaller for the Booster Date model because it reflects respondents who received a booster and for whom their date of booster receipt was validated, as discussed in S1.4.2**.**

In addition to the validation Tables S15, S16, and S17 provide for the LCCA procedure, Tables S18 and S19 further illustrate the bivariate relationships between segment membership and the frequency of mask use and social distancing across all five CABS survey waves. Results show that four out of five segments reported less frequent mask use and social distancing compared with the Ready Confident reference group (*P <* .01 for social distancing among Prevention-Compliant Non-Intenders during Wave 5, *P <* .001 for all other groups’ coefficients). On average, Anxious Waiter respondents did not differ significantly from Ready Confident respondents with respect to these behaviors (except for in Wave 1, during which Anxious Waiters reported significantly less social distancing compared with Ready Confidents at *P <* .05). Additionally, while a greater proportion of Burned-Out Waiters reported COVID-19 vaccination compared to the Prevention-Compliant Non-Intenders, the Prevention-Compliant Non-Intenders reported a greater frequency of mask use and social distancing compared with the Burned-Out Waiters.

| *Table S18: Survey-Weighted Regression of Wave 1 Clusters on Mask Use by Wave* | | | | | |
| --- | --- | --- | --- | --- | --- |
|  | Wave 1 (Std. Err.) | Wave 2 (Std. Err.) | Wave 3 (Std. Err.) | Wave 4 (Std. Err.) | Wave 5 (Std. Err.) |
| Hardline Non-Intenders^a^ | -2.11*** | -2.22*** | -2.22*** | -2.45*** | -1.94*** |
|  | (0.07) | (0.09) | (0.09) | (0.10) | (0.11) |
| Prevention-Compliant Non-Intenders | -0.21*** | -0.36*** | -0.61*** | -0.67*** | -0.82*** |
|  | (0.04) | (0.09) | (0.11) | (0.10) | (0.13) |
| Burned-Out Waiters | -0.90*** | -1.01*** | -1.20*** | -1.32*** | -1.13*** |
|  | (0.06) | (0.06) | (0.09) | (0.09) | (0.08) |
| Anxious Waiters | -0.02 | 0.05 | 0.04 | -0.09 | 0.06 |
|  | (0.02) | (0.06) | (0.06) | (0.06) | (0.10) |
| Skeptical Confidents | -0.23*** | -0.40*** | -0.54*** | -0.57*** | -0.58*** |
|  | (0.02) | (0.05) | (0.05) | (0.06) | (0.06) |
| Intercept | 4.87*** | 4.40*** | 4.15*** | 4.31*** | 3.30*** |
|  | (0.01) | (0.02) | (0.04) | (0.04) | (0.06) |
| N | 4396 | 3960 | 3641 | 3394 | 3255 |
| R-Squared | 0.47 | 0.28 | 0.26 | 0.31 | 0.19 |

*** *P* < .001, ** *P* < .01, * *P* < .05

^a^ Ready Confidents serve as the reference group for this analysis. Sample size is reduced across models due to panel attrition across waves.

| *Table S19: Survey-Weighted Regression of Wave 1 Clusters on Social Distancing by Wave* | | | | | |
| --- | --- | --- | --- | --- | --- |
|  | Wave 1 (Std. Err.) | Wave 2 (Std. Err.) | Wave 3 (Std. Err.) | Wave 4 (Std. Err.) | Wave 5 (Std. Err.) |
| Hardline Non-Intenders^a^ | -1.58*** | -1.54*** | -1.61*** | -1.70*** | -1.65*** |
|  | (0.07) | (0.10) | (0.10) | (0.13) | (0.09) |
| Prevention-Compliant Non-Intenders | -0.37*** | -0.41*** | -0.40*** | -0.41*** | -0.50** |
|  | (0.09) | (0.10) | (0.10) | (0.09) | (0.14) |
| Burned-Out Waiters | -0.95*** | -0.89*** | -0.95*** | -0.90*** | -1.00*** |
|  | (0.06) | (0.05) | (0.06) | (0.07) | (0.07) |
| Anxious Waiters | -0.09* | <-0.01 | <-0.01 | -0.06 | -0.08 |
|  | (0.03) | (0.05) | (0.06) | (0.07) | (0.08) |
| Skeptical Confidents | -0.28*** | -0.32*** | -0.34*** | -0.38*** | -0.48*** |
|  | (0.03) | (0.04) | (0.05) | (0.06) | (0.07) |
| Intercept | 4.44*** | 4.05*** | 3.89*** | 3.95*** | 3.54*** |
|  | (0.02) | (0.02) | (0.03) | (0.03) | (0.04) |
| N | 4396 | 3960 | 3640 | 3392 | 3258 |
| R-Squared | 0.26 | 0.19 | 0.19 | 0.18 | 0.16 |
| *** *P* < .001, ** *P* < .01, * *P* < .05  ^a^ Ready Confidents serve as the reference group for this analysis. Sample size is reduced across models due to panel attrition across waves. | | | | | |

**S2.3 Survival Tables**

This section reports on tables of respondents at risk, failures, net lost counts, and the failure function graphed in Figures 1 and 2 in the manuscript. Each table is separated by outcome (i.e., vaccine uptake and booster uptake) and by Wave 1 Cluster.

| *Table S20. Hardline Non-Intender Vaccine Uptake Survival Table* | | | | |
| --- | --- | --- | --- | --- |
| Time | At risk | Fail | Net lost | Failure function |
| 12/1/2020 | 0.00 | 0.00 | -187.01 | 0.000 |
| 2/4/2021 | 187.01 | 0.79 | 0.00 | 0.004 |
| 2/10/2021 | 186.22 | 1.01 | 0.00 | 0.010 |
| 3/5/2021 | 185.21 | 0.98 | 0.00 | 0.015 |
| 3/20/2021 | 184.23 | 0.38 | 0.00 | 0.017 |
| 4/11/2021 | 183.85 | 0.80 | 0.00 | 0.021 |
| 4/20/2021 | 183.05 | 0.44 | 0.00 | 0.023 |
| 4/23/2021 | 182.61 | 1.19 | 0.00 | 0.030 |
| 4/24/2021 | 181.42 | 1.72 | 0.00 | 0.039 |
| 5/8/2021 | 179.69 | 0.57 | 0.00 | 0.042 |
| 5/10/2021 | 179.13 | 1.00 | 0.00 | 0.047 |
| 5/14/2021 | 178.13 | 4.22 | 0.00 | 0.070 |
| 5/20/2021 | 173.91 | 3.65 | 0.00 | 0.090 |
| 5/21/2021 | 170.25 | 1.58 | 0.00 | 0.098 |
| 5/26/2021 | 168.68 | 0.30 | 0.00 | 0.100 |
| 6/4/2021 | 168.37 | 1.55 | 0.00 | 0.108 |
| 6/5/2021 | 166.83 | 0.29 | 0.00 | 0.109 |
| 6/8/2021 | 166.54 | 1.16 | 0.00 | 0.116 |
| 7/10/2021 | 165.38 | 0.46 | 0.00 | 0.118 |
| 7/21/2021 | 164.93 | 2.75 | 0.00 | 0.133 |
| 8/6/2021 | 162.18 | 0.70 | 0.00 | 0.136 |
| 8/7/2021 | 161.48 | 7.59 | 0.00 | 0.177 |
| 8/21/2021 | 153.89 | 0.73 | 0.00 | 0.181 |
| 9/1/2021 | 153.16 | 1.39 | 0.00 | 0.188 |
| 9/3/2021 | 151.77 | 2.91 | 0.00 | 0.204 |
| 9/9/2021 | 148.86 | 3.77 | 0.00 | 0.224 |
| 9/11/2021 | 145.09 | 0.85 | 0.00 | 0.229 |
| 10/6/2021 | 144.24 | 3.66 | 0.00 | 0.248 |
| 10/20/2021 | 140.58 | 0.68 | 0.00 | 0.252 |
| 10/23/2021 | 139.90 | 0.78 | 0.00 | 0.256 |
| 10/30/2021 | 139.12 | 0.34 | 0.00 | 0.258 |
| 11/19/2021 | 138.78 | 0.39 | 0.00 | 0.260 |
| 11/23/2021 | 138.39 | 1.29 | 0.00 | 0.267 |
| 12/3/2021 | 137.10 | 0.77 | 0.00 | 0.271 |
| 12/11/2021 | 136.32 | 0.19 | 0.00 | 0.272 |
| 12/15/2021 | 136.14 | 0.71 | 0.00 | 0.276 |
| 2/18/2022 | 135.43 | 0.35 | 0.00 | 0.278 |
| 5/26/2022 | 135.08 | 0.00 | 5.64 | 0.278 |
| 5/27/2022 | 129.44 | 0.00 | 43.52 | 0.278 |
| 5/28/2022 | 85.92 | 0.00 | 29.92 | 0.278 |
| 5/29/2022 | 55.99 | 0.00 | 13.49 | 0.278 |
| 5/30/2022 | 42.51 | 0.00 | 5.72 | 0.278 |
| 5/31/2022 | 36.78 | 0.00 | 7.31 | 0.278 |
| 6/1/2022 | 29.47 | 0.00 | 5.52 | 0.278 |
| 6/2/2022 | 23.95 | 0.00 | 6.00 | 0.278 |
| 6/3/2022 | 17.95 | 0.00 | 1.94 | 0.278 |
| 6/4/2022 | 16.02 | 0.00 | 0.49 | 0.278 |
| 6/5/2022 | 15.53 | 0.00 | 0.82 | 0.278 |
| 6/6/2022 | 14.70 | 0.00 | 0.67 | 0.278 |
| 6/7/2022 | 14.03 | 0.00 | 1.18 | 0.278 |
| 6/8/2022 | 12.85 | 0.00 | 0.53 | 0.278 |
| 6/9/2022 | 12.32 | 0.00 | 0.70 | 0.278 |
| 6/10/2022 | 11.62 | 0.00 | 0.76 | 0.278 |
| 6/12/2022 | 10.86 | 0.00 | 2.64 | 0.278 |
| 6/13/2022 | 8.22 | 0.00 | 1.01 | 0.278 |
| 6/14/2022 | 7.21 | 0.00 | 0.81 | 0.278 |
| 6/16/2022 | 6.40 | 0.00 | 0.82 | 0.278 |
| 6/22/2022 | 5.58 | 0.00 | 0.41 | 0.278 |
| 6/23/2022 | 5.17 | 0.00 | 1.11 | 0.278 |
| 6/26/2022 | 4.07 | 0.00 | 2.87 | 0.278 |
| 6/27/2022 | 1.20 | 0.00 | 0.22 | 0.278 |
| 7/1/2022 | 0.98 | 0.00 | 0.35 | 0.278 |

| *Table S21. Prevention-Compliant Non-Intender Vaccine Uptake Survival Table* | | | | |
| --- | --- | --- | --- | --- |
| Time | At risk | Fail | Net lost | Failure function |
| 12/1/2020 | 0.00 | 0.00 | -209.54 | 0.000 |
| 2/19/2021 | 209.54 | 1.44 | 0.00 | 0.007 |
| 3/2/2021 | 208.10 | 0.66 | 0.00 | 0.010 |
| 3/6/2021 | 207.44 | 0.47 | 0.00 | 0.012 |
| 3/7/2021 | 206.96 | 0.41 | 0.00 | 0.014 |
| 3/19/2021 | 206.55 | 0.35 | 0.00 | 0.016 |
| 3/20/2021 | 206.20 | 0.51 | 0.00 | 0.018 |
| 3/22/2021 | 205.69 | 1.12 | 0.00 | 0.024 |
| 3/23/2021 | 204.56 | 0.56 | 0.00 | 0.026 |
| 4/1/2021 | 204.00 | 3.90 | 0.00 | 0.045 |
| 4/3/2021 | 200.10 | 1.43 | 0.00 | 0.052 |
| 4/8/2021 | 198.67 | 1.69 | 0.00 | 0.060 |
| 4/10/2021 | 196.98 | 0.37 | 0.00 | 0.062 |
| 4/14/2021 | 196.61 | 2.49 | 0.00 | 0.074 |
| 4/15/2021 | 194.12 | 0.75 | 0.00 | 0.077 |
| 4/19/2021 | 193.37 | 0.58 | 0.00 | 0.080 |
| 4/21/2021 | 192.79 | 1.18 | 0.00 | 0.086 |
| 4/27/2021 | 191.60 | 2.44 | 0.00 | 0.097 |
| 5/1/2021 | 189.16 | 0.89 | 0.00 | 0.101 |
| 5/2/2021 | 188.28 | 0.39 | 0.00 | 0.103 |
| 5/7/2021 | 187.88 | 2.07 | 0.00 | 0.113 |
| 5/10/2021 | 185.81 | 1.82 | 0.00 | 0.122 |
| 5/15/2021 | 184.00 | 1.98 | 0.00 | 0.131 |
| 5/16/2021 | 182.02 | 1.03 | 0.00 | 0.136 |
| 5/18/2021 | 181.00 | 0.47 | 0.00 | 0.138 |
| 5/20/2021 | 180.53 | 1.55 | 0.00 | 0.146 |
| 6/10/2021 | 178.98 | 1.07 | 0.00 | 0.151 |
| 6/18/2021 | 177.91 | 0.98 | 0.00 | 0.156 |
| 6/19/2021 | 176.93 | 0.28 | 0.00 | 0.157 |
| 6/20/2021 | 176.64 | 0.75 | 0.00 | 0.161 |
| 7/7/2021 | 175.89 | 1.05 | 0.00 | 0.166 |
| 7/12/2021 | 174.84 | 1.86 | 0.00 | 0.174 |
| 7/17/2021 | 172.99 | 0.48 | 0.00 | 0.177 |
| 7/20/2021 | 172.51 | 0.85 | 0.00 | 0.181 |
| 7/30/2021 | 171.66 | 0.19 | 0.00 | 0.182 |
| 7/31/2021 | 171.47 | 0.44 | 0.00 | 0.184 |
| 8/1/2021 | 171.03 | 4.15 | 0.00 | 0.204 |
| 8/6/2021 | 166.88 | 0.41 | 0.00 | 0.206 |
| 8/7/2021 | 166.46 | 1.72 | 0.00 | 0.214 |
| 8/8/2021 | 164.74 | 1.51 | 0.00 | 0.221 |
| 8/10/2021 | 163.23 | 1.56 | 0.00 | 0.228 |
| 8/12/2021 | 161.67 | 1.28 | 0.00 | 0.235 |
| 8/21/2021 | 160.39 | 2.17 | 0.00 | 0.245 |
| 8/24/2021 | 158.21 | 0.73 | 0.00 | 0.248 |
| 8/28/2021 | 157.49 | 4.78 | 0.00 | 0.271 |
| 9/1/2021 | 152.71 | 0.34 | 0.00 | 0.273 |
| 9/3/2021 | 152.37 | 0.85 | 0.00 | 0.277 |
| 9/4/2021 | 151.52 | 1.90 | 0.00 | 0.286 |
| 9/6/2021 | 149.62 | 2.78 | 0.00 | 0.299 |
| 9/7/2021 | 146.84 | 0.64 | 0.00 | 0.302 |
| 9/9/2021 | 146.20 | 0.77 | 0.00 | 0.306 |
| 9/10/2021 | 145.43 | 5.97 | 0.00 | 0.334 |
| 9/11/2021 | 139.46 | 2.15 | 0.00 | 0.345 |
| 9/16/2021 | 137.31 | 0.87 | 0.00 | 0.349 |
| 9/22/2021 | 136.44 | 0.62 | 0.00 | 0.352 |
| 9/29/2021 | 135.82 | 1.04 | 0.00 | 0.357 |
| 9/30/2021 | 134.78 | 1.22 | 0.00 | 0.363 |
| 10/4/2021 | 133.56 | 0.80 | 0.00 | 0.366 |
| 10/17/2021 | 132.76 | 0.11 | 0.00 | 0.367 |
| 10/20/2021 | 132.65 | 0.42 | 0.00 | 0.369 |
| 10/29/2021 | 132.23 | 0.52 | 0.00 | 0.371 |
| 11/9/2021 | 131.71 | 0.36 | 0.00 | 0.373 |
| 11/19/2021 | 131.35 | 1.08 | 0.00 | 0.378 |
| 11/22/2021 | 130.27 | 0.39 | 0.00 | 0.380 |
| 11/30/2021 | 129.87 | 0.60 | 0.00 | 0.383 |
| 12/4/2021 | 129.27 | 0.21 | 0.00 | 0.384 |
| 12/10/2021 | 129.06 | 0.22 | 0.00 | 0.385 |
| 12/19/2021 | 128.84 | 0.67 | 0.00 | 0.388 |
| 1/15/2022 | 128.17 | 0.55 | 0.00 | 0.391 |
| 1/18/2022 | 127.62 | 1.00 | 0.00 | 0.396 |
| 1/20/2022 | 126.62 | 0.46 | 0.00 | 0.398 |
| 2/24/2022 | 126.16 | 1.05 | 0.00 | 0.403 |
| 3/18/2022 | 125.11 | 0.45 | 0.00 | 0.405 |
| 5/1/2022 | 124.65 | 0.26 | 0.00 | 0.406 |
| 5/26/2022 | 124.40 | 0.00 | 9.90 | 0.406 |
| 5/27/2022 | 114.49 | 0.00 | 46.60 | 0.406 |
| 5/28/2022 | 67.89 | 0.00 | 28.41 | 0.406 |
| 5/29/2022 | 39.48 | 0.00 | 6.62 | 0.406 |
| 5/30/2022 | 32.86 | 0.00 | 0.41 | 0.406 |
| 5/31/2022 | 32.44 | 0.00 | 0.13 | 0.406 |
| 6/1/2022 | 32.31 | 0.00 | 5.08 | 0.406 |
| 6/2/2022 | 27.23 | 0.00 | 2.36 | 0.406 |
| 6/3/2022 | 24.87 | 0.00 | 3.28 | 0.406 |
| 6/4/2022 | 21.59 | 0.00 | 4.80 | 0.406 |
| 6/5/2022 | 16.79 | 0.00 | 1.43 | 0.406 |
| 6/7/2022 | 15.36 | 0.00 | 6.93 | 0.406 |
| 6/8/2022 | 8.43 | 0.00 | 0.87 | 0.406 |
| 6/12/2022 | 7.56 | 0.00 | 1.22 | 0.406 |
| 6/13/2022 | 6.34 | 0.00 | 1.75 | 0.406 |
| 6/14/2022 | 4.59 | 0.00 | 0.88 | 0.406 |
| 6/15/2022 | 3.71 | 0.00 | 0.74 | 0.406 |
| 6/16/2022 | 2.97 | 0.00 | 0.64 | 0.406 |
| 6/17/2022 | 2.32 | 0.00 | 1.14 | 0.406 |
| 6/19/2022 | 1.18 | 0.00 | 0.15 | 0.406 |
| 6/20/2022 | 1.03 | 0.00 | 0.26 | 0.406 |
| 6/24/2022 | 0.77 | 0.00 | 0.14 | 0.406 |

| *Table S22. Burned Out Waiter Vaccine Uptake Survival Table* | | | | |
| --- | --- | --- | --- | --- |
| Time | At risk | Fail | Net lost | Failure function |
| 12/1/2020 | 0.00 | 0.00 | -439.72 | 0.000 |
| 12/28/2020 | 439.72 | 2.37 | 0.00 | 0.005 |
| 12/29/2020 | 437.34 | 0.50 | 0.00 | 0.007 |
| 1/10/2021 | 436.85 | 2.57 | 0.00 | 0.012 |
| 1/11/2021 | 434.28 | 0.33 | 0.00 | 0.013 |
| 1/14/2021 | 433.95 | 0.52 | 0.00 | 0.014 |
| 1/15/2021 | 433.43 | 0.86 | 0.00 | 0.016 |
| 1/17/2021 | 432.57 | 0.60 | 0.00 | 0.018 |
| 1/20/2021 | 431.97 | 0.59 | 0.00 | 0.019 |
| 1/21/2021 | 431.38 | 0.65 | 0.00 | 0.020 |
| 1/22/2021 | 430.73 | 0.39 | 0.00 | 0.021 |
| 1/23/2021 | 430.34 | 1.10 | 0.00 | 0.024 |
| 1/28/2021 | 429.24 | 0.72 | 0.00 | 0.025 |
| 2/2/2021 | 428.52 | 0.36 | 0.00 | 0.026 |
| 2/4/2021 | 428.16 | 4.74 | 0.00 | 0.037 |
| 2/8/2021 | 423.42 | 0.34 | 0.00 | 0.038 |
| 2/10/2021 | 423.08 | 1.10 | 0.00 | 0.040 |
| 2/11/2021 | 421.99 | 0.56 | 0.00 | 0.042 |
| 2/12/2021 | 421.43 | 1.96 | 0.00 | 0.046 |
| 2/14/2021 | 419.46 | 0.37 | 0.00 | 0.047 |
| 2/15/2021 | 419.09 | 1.34 | 0.00 | 0.050 |
| 2/16/2021 | 417.74 | 1.71 | 0.00 | 0.054 |
| 2/17/2021 | 416.04 | 1.39 | 0.00 | 0.057 |
| 2/19/2021 | 414.64 | 0.61 | 0.00 | 0.058 |
| 2/23/2021 | 414.03 | 0.69 | 0.00 | 0.060 |
| 2/24/2021 | 413.34 | 1.64 | 0.00 | 0.064 |
| 2/26/2021 | 411.70 | 0.38 | 0.00 | 0.065 |
| 3/1/2021 | 411.32 | 1.76 | 0.00 | 0.069 |
| 3/2/2021 | 409.56 | 1.45 | 0.00 | 0.072 |
| 3/3/2021 | 408.11 | 2.39 | 0.00 | 0.077 |
| 3/4/2021 | 405.72 | 1.50 | 0.00 | 0.081 |
| 3/5/2021 | 404.21 | 1.01 | 0.00 | 0.083 |
| 3/6/2021 | 403.20 | 0.71 | 0.00 | 0.085 |
| 3/7/2021 | 402.49 | 3.60 | 0.00 | 0.093 |
| 3/8/2021 | 398.89 | 1.94 | 0.00 | 0.097 |
| 3/9/2021 | 396.95 | 1.16 | 0.00 | 0.100 |
| 3/10/2021 | 395.78 | 1.14 | 0.00 | 0.103 |
| 3/11/2021 | 394.64 | 0.35 | 0.00 | 0.103 |
| 3/12/2021 | 394.29 | 2.78 | 0.00 | 0.110 |
| 3/14/2021 | 391.51 | 1.11 | 0.00 | 0.112 |
| 3/15/2021 | 390.39 | 2.70 | 0.00 | 0.118 |
| 3/17/2021 | 387.70 | 1.79 | 0.00 | 0.122 |
| 3/18/2021 | 385.91 | 3.03 | 0.00 | 0.129 |
| 3/19/2021 | 382.88 | 0.30 | 0.00 | 0.130 |
| 3/20/2021 | 382.58 | 0.71 | 0.00 | 0.132 |
| 3/21/2021 | 381.87 | 0.44 | 0.00 | 0.133 |
| 3/22/2021 | 381.43 | 2.14 | 0.00 | 0.137 |
| 3/23/2021 | 379.29 | 2.60 | 0.00 | 0.143 |
| 3/24/2021 | 376.69 | 0.90 | 0.00 | 0.145 |
| 3/25/2021 | 375.79 | 2.17 | 0.00 | 0.150 |
| 3/27/2021 | 373.62 | 4.65 | 0.00 | 0.161 |
| 3/31/2021 | 368.96 | 3.09 | 0.00 | 0.168 |
| 4/1/2021 | 365.87 | 3.25 | 0.00 | 0.175 |
| 4/2/2021 | 362.62 | 0.58 | 0.00 | 0.177 |
| 4/3/2021 | 362.04 | 0.93 | 0.00 | 0.179 |
| 4/5/2021 | 361.11 | 2.55 | 0.00 | 0.185 |
| 4/6/2021 | 358.57 | 0.88 | 0.00 | 0.187 |
| 4/7/2021 | 357.68 | 2.98 | 0.00 | 0.193 |
| 4/8/2021 | 354.71 | 2.52 | 0.00 | 0.199 |
| 4/9/2021 | 352.19 | 0.45 | 0.00 | 0.200 |
| 4/10/2021 | 351.73 | 3.41 | 0.00 | 0.208 |
| 4/12/2021 | 348.32 | 2.18 | 0.00 | 0.213 |
| 4/13/2021 | 346.15 | 1.45 | 0.00 | 0.216 |
| 4/15/2021 | 344.70 | 2.70 | 0.00 | 0.222 |
| 4/19/2021 | 342.00 | 0.85 | 0.00 | 0.224 |
| 4/20/2021 | 341.15 | 4.84 | 0.00 | 0.235 |
| 4/23/2021 | 336.31 | 1.22 | 0.00 | 0.238 |
| 4/24/2021 | 335.09 | 0.98 | 0.00 | 0.240 |
| 4/26/2021 | 334.11 | 0.66 | 0.00 | 0.242 |
| 4/27/2021 | 333.45 | 3.48 | 0.00 | 0.250 |
| 4/30/2021 | 329.97 | 3.44 | 0.00 | 0.257 |
| 5/1/2021 | 326.53 | 12.80 | 0.00 | 0.287 |
| 5/2/2021 | 313.74 | 0.64 | 0.00 | 0.288 |
| 5/4/2021 | 313.09 | 1.93 | 0.00 | 0.292 |
| 5/5/2021 | 311.16 | 2.85 | 0.00 | 0.299 |
| 5/6/2021 | 308.31 | 1.35 | 0.00 | 0.302 |
| 5/7/2021 | 306.96 | 1.15 | 0.00 | 0.305 |
| 5/8/2021 | 305.80 | 5.98 | 0.00 | 0.318 |
| 5/9/2021 | 299.82 | 0.45 | 0.00 | 0.319 |
| 5/10/2021 | 299.37 | 1.51 | 0.00 | 0.323 |
| 5/12/2021 | 297.86 | 3.79 | 0.00 | 0.331 |
| 5/14/2021 | 294.08 | 4.48 | 0.00 | 0.341 |
| 5/15/2021 | 289.59 | 4.26 | 0.00 | 0.351 |
| 5/19/2021 | 285.34 | 3.49 | 0.00 | 0.359 |
| 5/23/2021 | 281.85 | 5.84 | 0.00 | 0.372 |
| 5/25/2021 | 276.01 | 1.40 | 0.00 | 0.375 |
| 5/27/2021 | 274.61 | 7.33 | 0.00 | 0.392 |
| 5/30/2021 | 267.28 | 1.28 | 0.00 | 0.395 |
| 6/1/2021 | 266.00 | 0.73 | 0.00 | 0.397 |
| 6/3/2021 | 265.27 | 1.55 | 0.00 | 0.400 |
| 6/4/2021 | 263.72 | 3.52 | 0.00 | 0.408 |
| 6/5/2021 | 260.20 | 2.76 | 0.00 | 0.415 |
| 6/7/2021 | 257.44 | 1.96 | 0.00 | 0.419 |
| 6/8/2021 | 255.48 | 1.16 | 0.00 | 0.422 |
| 6/9/2021 | 254.32 | 2.94 | 0.00 | 0.428 |
| 6/12/2021 | 251.38 | 0.68 | 0.00 | 0.430 |
| 6/14/2021 | 250.71 | 0.26 | 0.00 | 0.430 |
| 6/22/2021 | 250.44 | 0.66 | 0.00 | 0.432 |
| 6/26/2021 | 249.78 | 2.40 | 0.00 | 0.437 |
| 6/30/2021 | 247.38 | 0.94 | 0.00 | 0.440 |
| 7/2/2021 | 246.45 | 2.03 | 0.00 | 0.444 |
| 7/3/2021 | 244.41 | 0.62 | 0.00 | 0.446 |
| 7/7/2021 | 243.79 | 0.54 | 0.00 | 0.447 |
| 7/8/2021 | 243.24 | 7.80 | 0.00 | 0.465 |
| 7/18/2021 | 235.44 | 0.58 | 0.00 | 0.466 |
| 7/27/2021 | 234.86 | 7.45 | 0.00 | 0.483 |
| 7/28/2021 | 227.41 | 3.64 | 0.00 | 0.491 |
| 7/30/2021 | 223.76 | 0.46 | 0.00 | 0.492 |
| 8/4/2021 | 223.30 | 2.00 | 0.00 | 0.497 |
| 8/6/2021 | 221.30 | 0.51 | 0.00 | 0.498 |
| 8/8/2021 | 220.79 | 2.88 | 0.00 | 0.504 |
| 8/9/2021 | 217.92 | 2.01 | 0.00 | 0.509 |
| 8/10/2021 | 215.91 | 0.42 | 0.00 | 0.510 |
| 8/13/2021 | 215.49 | 0.95 | 0.00 | 0.512 |
| 8/15/2021 | 214.54 | 0.68 | 0.00 | 0.514 |
| 8/20/2021 | 213.86 | 1.43 | 0.00 | 0.517 |
| 8/27/2021 | 212.43 | 3.71 | 0.00 | 0.525 |
| 8/30/2021 | 208.71 | 0.88 | 0.00 | 0.527 |
| 9/2/2021 | 207.83 | 1.70 | 0.00 | 0.531 |
| 9/3/2021 | 206.13 | 5.66 | 0.00 | 0.544 |
| 9/6/2021 | 200.47 | 0.58 | 0.00 | 0.545 |
| 9/7/2021 | 199.89 | 2.53 | 0.00 | 0.551 |
| 9/8/2021 | 197.36 | 3.44 | 0.00 | 0.559 |
| 9/9/2021 | 193.91 | 5.47 | 0.00 | 0.571 |
| 9/12/2021 | 188.44 | 1.14 | 0.00 | 0.574 |
| 9/16/2021 | 187.31 | 10.27 | 0.00 | 0.597 |
| 9/19/2021 | 177.03 | 6.86 | 0.00 | 0.613 |
| 9/24/2021 | 170.17 | 5.03 | 0.00 | 0.624 |
| 9/28/2021 | 165.14 | 3.16 | 0.00 | 0.632 |
| 10/23/2021 | 161.98 | 0.48 | 0.00 | 0.633 |
| 11/3/2021 | 161.50 | 0.37 | 0.00 | 0.634 |
| 11/7/2021 | 161.13 | 1.40 | 0.00 | 0.637 |
| 11/17/2021 | 159.74 | 0.48 | 0.00 | 0.638 |
| 11/19/2021 | 159.25 | 1.18 | 0.00 | 0.641 |
| 11/26/2021 | 158.08 | 0.54 | 0.00 | 0.642 |
| 12/5/2021 | 157.54 | 0.55 | 0.00 | 0.643 |
| 12/13/2021 | 156.99 | 0.70 | 0.00 | 0.645 |
| 12/31/2021 | 156.28 | 0.45 | 0.00 | 0.646 |
| 1/1/2022 | 155.83 | 0.57 | 0.00 | 0.647 |
| 1/5/2022 | 155.26 | 0.59 | 0.00 | 0.648 |
| 1/17/2022 | 154.67 | 0.78 | 0.00 | 0.650 |
| 2/1/2022 | 153.90 | 0.60 | 0.00 | 0.651 |
| 3/10/2022 | 153.30 | 0.38 | 0.00 | 0.652 |
| 5/21/2022 | 152.92 | 1.88 | 0.00 | 0.657 |
| 5/26/2022 | 151.04 | 0.00 | 14.11 | 0.657 |
| 5/27/2022 | 136.93 | 0.00 | 45.68 | 0.657 |
| 5/28/2022 | 91.25 | 0.00 | 20.45 | 0.657 |
| 5/29/2022 | 70.80 | 0.00 | 14.98 | 0.657 |
| 5/30/2022 | 55.82 | 0.00 | 6.90 | 0.657 |
| 5/31/2022 | 48.92 | 0.00 | 4.09 | 0.657 |
| 6/1/2022 | 44.83 | 0.00 | 7.80 | 0.657 |
| 6/2/2022 | 37.02 | 0.00 | 8.60 | 0.657 |
| 6/4/2022 | 28.43 | 0.00 | 2.35 | 0.657 |
| 6/5/2022 | 26.07 | 0.00 | 5.74 | 0.657 |
| 6/6/2022 | 20.33 | 0.00 | 1.75 | 0.657 |
| 6/7/2022 | 18.59 | 0.00 | 2.55 | 0.657 |
| 6/8/2022 | 16.03 | 0.00 | 0.58 | 0.657 |
| 6/9/2022 | 15.45 | 0.00 | 2.44 | 0.657 |
| 6/10/2022 | 13.01 | 0.00 | 1.33 | 0.657 |
| 6/11/2022 | 11.68 | 0.00 | 1.25 | 0.657 |
| 6/12/2022 | 10.43 | 0.00 | 3.60 | 0.657 |
| 6/13/2022 | 6.83 | 0.00 | 0.70 | 0.657 |
| 6/14/2022 | 6.13 | 0.00 | 0.28 | 0.657 |
| 6/18/2022 | 5.85 | 0.00 | 0.79 | 0.657 |
| 6/20/2022 | 5.06 | 0.00 | 1.62 | 0.657 |
| 6/22/2022 | 3.45 | 0.00 | 0.70 | 0.657 |
| 6/23/2022 | 2.75 | 0.00 | 1.27 | 0.657 |
| 6/24/2022 | 1.47 | 0.00 | 0.83 | 0.657 |

| *Table S23. Anxious Waiter Vaccine Uptake Survival Table* | | | | |
| --- | --- | --- | --- | --- |
| Time | At risk | Fail | Net lost | Failure function |
| 12/1/2020 | 0.00 | 0.00 | -441.57 | 0.000 |
| 1/11/2021 | 441.57 | 0.77 | 0.00 | 0.002 |
| 1/21/2021 | 440.80 | 2.32 | 0.00 | 0.007 |
| 1/28/2021 | 438.48 | 0.83 | 0.00 | 0.009 |
| 2/1/2021 | 437.65 | 0.60 | 0.00 | 0.010 |
| 2/2/2021 | 437.04 | 1.51 | 0.00 | 0.014 |
| 2/6/2021 | 435.54 | 0.31 | 0.00 | 0.014 |
| 2/7/2021 | 435.23 | 0.96 | 0.00 | 0.017 |
| 2/9/2021 | 434.27 | 1.30 | 0.00 | 0.019 |
| 2/12/2021 | 432.97 | 0.94 | 0.00 | 0.022 |
| 2/15/2021 | 432.03 | 1.65 | 0.00 | 0.025 |
| 2/17/2021 | 430.39 | 0.38 | 0.00 | 0.026 |
| 2/20/2021 | 430.01 | 0.18 | 0.00 | 0.027 |
| 2/21/2021 | 429.83 | 0.15 | 0.00 | 0.027 |
| 2/24/2021 | 429.68 | 0.68 | 0.00 | 0.028 |
| 2/25/2021 | 429.01 | 0.32 | 0.00 | 0.029 |
| 2/28/2021 | 428.68 | 2.23 | 0.00 | 0.034 |
| 3/1/2021 | 426.45 | 1.79 | 0.00 | 0.038 |
| 3/2/2021 | 424.66 | 0.72 | 0.00 | 0.040 |
| 3/3/2021 | 423.94 | 1.62 | 0.00 | 0.044 |
| 3/4/2021 | 422.32 | 3.47 | 0.00 | 0.051 |
| 3/5/2021 | 418.85 | 1.65 | 0.00 | 0.055 |
| 3/8/2021 | 417.20 | 3.55 | 0.00 | 0.063 |
| 3/9/2021 | 413.65 | 0.84 | 0.00 | 0.065 |
| 3/10/2021 | 412.81 | 2.04 | 0.00 | 0.070 |
| 3/11/2021 | 410.77 | 5.30 | 0.00 | 0.082 |
| 3/12/2021 | 405.48 | 1.86 | 0.00 | 0.086 |
| 3/13/2021 | 403.61 | 2.65 | 0.00 | 0.092 |
| 3/14/2021 | 400.96 | 3.98 | 0.00 | 0.101 |
| 3/15/2021 | 396.98 | 1.27 | 0.00 | 0.104 |
| 3/16/2021 | 395.71 | 2.90 | 0.00 | 0.110 |
| 3/17/2021 | 392.81 | 4.33 | 0.00 | 0.120 |
| 3/18/2021 | 388.48 | 0.83 | 0.00 | 0.122 |
| 3/19/2021 | 387.64 | 0.41 | 0.00 | 0.123 |
| 3/20/2021 | 387.23 | 2.16 | 0.00 | 0.128 |
| 3/21/2021 | 385.08 | 1.54 | 0.00 | 0.131 |
| 3/23/2021 | 383.54 | 0.50 | 0.00 | 0.133 |
| 3/25/2021 | 383.04 | 0.69 | 0.00 | 0.134 |
| 3/26/2021 | 382.35 | 0.45 | 0.00 | 0.135 |
| 3/27/2021 | 381.90 | 1.29 | 0.00 | 0.138 |
| 3/29/2021 | 380.61 | 5.52 | 0.00 | 0.151 |
| 3/30/2021 | 375.09 | 8.64 | 0.00 | 0.170 |
| 3/31/2021 | 366.45 | 1.01 | 0.00 | 0.172 |
| 4/1/2021 | 365.44 | 3.03 | 0.00 | 0.179 |
| 4/2/2021 | 362.40 | 4.02 | 0.00 | 0.188 |
| 4/3/2021 | 358.38 | 0.97 | 0.00 | 0.191 |
| 4/4/2021 | 357.41 | 0.87 | 0.00 | 0.193 |
| 4/5/2021 | 356.54 | 4.09 | 0.00 | 0.202 |
| 4/6/2021 | 352.45 | 4.95 | 0.00 | 0.213 |
| 4/7/2021 | 347.50 | 1.14 | 0.00 | 0.216 |
| 4/8/2021 | 346.35 | 2.49 | 0.00 | 0.221 |
| 4/9/2021 | 343.86 | 2.29 | 0.00 | 0.226 |
| 4/10/2021 | 341.57 | 3.68 | 0.00 | 0.235 |
| 4/11/2021 | 337.89 | 1.46 | 0.00 | 0.238 |
| 4/12/2021 | 336.43 | 0.79 | 0.00 | 0.240 |
| 4/13/2021 | 335.65 | 2.65 | 0.00 | 0.246 |
| 4/14/2021 | 333.00 | 1.25 | 0.00 | 0.249 |
| 4/15/2021 | 331.76 | 6.93 | 0.00 | 0.264 |
| 4/16/2021 | 324.82 | 1.42 | 0.00 | 0.268 |
| 4/17/2021 | 323.40 | 6.66 | 0.00 | 0.283 |
| 4/18/2021 | 316.75 | 0.11 | 0.00 | 0.283 |
| 4/19/2021 | 316.64 | 2.30 | 0.00 | 0.288 |
| 4/20/2021 | 314.33 | 3.43 | 0.00 | 0.296 |
| 4/22/2021 | 310.91 | 0.39 | 0.00 | 0.297 |
| 4/23/2021 | 310.52 | 2.14 | 0.00 | 0.302 |
| 4/24/2021 | 308.38 | 3.77 | 0.00 | 0.310 |
| 4/25/2021 | 304.61 | 0.93 | 0.00 | 0.312 |
| 4/27/2021 | 303.68 | 0.94 | 0.00 | 0.314 |
| 4/28/2021 | 302.74 | 2.73 | 0.00 | 0.321 |
| 4/30/2021 | 300.01 | 2.07 | 0.00 | 0.325 |
| 5/1/2021 | 297.94 | 5.53 | 0.00 | 0.338 |
| 5/2/2021 | 292.41 | 3.26 | 0.00 | 0.345 |
| 5/3/2021 | 289.15 | 2.38 | 0.00 | 0.351 |
| 5/4/2021 | 286.77 | 1.56 | 0.00 | 0.354 |
| 5/5/2021 | 285.22 | 4.42 | 0.00 | 0.364 |
| 5/6/2021 | 280.80 | 0.99 | 0.00 | 0.366 |
| 5/7/2021 | 279.81 | 2.68 | 0.00 | 0.372 |
| 5/8/2021 | 277.14 | 4.40 | 0.00 | 0.382 |
| 5/10/2021 | 272.73 | 9.54 | 0.00 | 0.404 |
| 5/11/2021 | 263.19 | 1.50 | 0.00 | 0.407 |
| 5/14/2021 | 261.69 | 0.74 | 0.00 | 0.409 |
| 5/15/2021 | 260.96 | 8.12 | 0.00 | 0.427 |
| 5/17/2021 | 252.84 | 1.62 | 0.00 | 0.431 |
| 5/18/2021 | 251.22 | 1.56 | 0.00 | 0.435 |
| 5/20/2021 | 249.66 | 0.80 | 0.00 | 0.436 |
| 5/21/2021 | 248.85 | 2.28 | 0.00 | 0.442 |
| 5/22/2021 | 246.57 | 1.01 | 0.00 | 0.444 |
| 5/24/2021 | 245.56 | 1.37 | 0.00 | 0.447 |
| 5/27/2021 | 244.18 | 2.23 | 0.00 | 0.452 |
| 5/28/2021 | 241.95 | 1.61 | 0.00 | 0.456 |
| 5/31/2021 | 240.35 | 1.53 | 0.00 | 0.459 |
| 6/1/2021 | 238.81 | 0.91 | 0.00 | 0.461 |
| 6/4/2021 | 237.91 | 2.36 | 0.00 | 0.467 |
| 6/8/2021 | 235.55 | 0.30 | 0.00 | 0.467 |
| 6/9/2021 | 235.25 | 4.49 | 0.00 | 0.477 |
| 6/13/2021 | 230.76 | 1.24 | 0.00 | 0.480 |
| 6/14/2021 | 229.52 | 1.26 | 0.00 | 0.483 |
| 6/15/2021 | 228.25 | 0.50 | 0.00 | 0.484 |
| 6/16/2021 | 227.75 | 0.37 | 0.00 | 0.485 |
| 6/17/2021 | 227.38 | 1.01 | 0.00 | 0.487 |
| 6/18/2021 | 226.36 | 2.86 | 0.00 | 0.494 |
| 6/19/2021 | 223.50 | 1.46 | 0.00 | 0.497 |
| 6/20/2021 | 222.04 | 2.57 | 0.00 | 0.503 |
| 6/21/2021 | 219.47 | 1.77 | 0.00 | 0.507 |
| 6/22/2021 | 217.70 | 3.02 | 0.00 | 0.514 |
| 6/23/2021 | 214.69 | 0.65 | 0.00 | 0.515 |
| 6/26/2021 | 214.03 | 2.79 | 0.00 | 0.522 |
| 7/2/2021 | 211.24 | 1.28 | 0.00 | 0.525 |
| 7/3/2021 | 209.96 | 1.05 | 0.00 | 0.527 |
| 7/10/2021 | 208.91 | 0.69 | 0.00 | 0.528 |
| 7/15/2021 | 208.22 | 0.79 | 0.00 | 0.530 |
| 7/21/2021 | 207.42 | 1.45 | 0.00 | 0.534 |
| 7/22/2021 | 205.97 | 2.67 | 0.00 | 0.540 |
| 7/23/2021 | 203.30 | 1.68 | 0.00 | 0.543 |
| 7/25/2021 | 201.62 | 3.10 | 0.00 | 0.550 |
| 7/27/2021 | 198.51 | 1.59 | 0.00 | 0.554 |
| 7/28/2021 | 196.92 | 1.15 | 0.00 | 0.557 |
| 7/29/2021 | 195.77 | 0.58 | 0.00 | 0.558 |
| 7/30/2021 | 195.19 | 0.33 | 0.00 | 0.559 |
| 8/2/2021 | 194.87 | 0.63 | 0.00 | 0.560 |
| 8/3/2021 | 194.23 | 1.67 | 0.00 | 0.564 |
| 8/5/2021 | 192.56 | 3.11 | 0.00 | 0.571 |
| 8/6/2021 | 189.46 | 2.28 | 0.00 | 0.576 |
| 8/7/2021 | 187.18 | 1.40 | 0.00 | 0.579 |
| 8/9/2021 | 185.77 | 0.32 | 0.00 | 0.580 |
| 8/10/2021 | 185.45 | 9.80 | 0.00 | 0.602 |
| 8/11/2021 | 175.65 | 1.12 | 0.00 | 0.605 |
| 8/13/2021 | 174.54 | 0.66 | 0.00 | 0.606 |
| 8/15/2021 | 173.88 | 3.90 | 0.00 | 0.615 |
| 8/16/2021 | 169.98 | 1.64 | 0.00 | 0.619 |
| 8/20/2021 | 168.35 | 1.99 | 0.00 | 0.623 |
| 8/24/2021 | 166.36 | 1.60 | 0.00 | 0.627 |
| 8/27/2021 | 164.75 | 0.49 | 0.00 | 0.628 |
| 8/30/2021 | 164.26 | 0.33 | 0.00 | 0.629 |
| 9/1/2021 | 163.93 | 1.88 | 0.00 | 0.633 |
| 9/2/2021 | 162.05 | 0.76 | 0.00 | 0.635 |
| 9/3/2021 | 161.29 | 6.19 | 0.00 | 0.649 |
| 9/6/2021 | 155.10 | 9.81 | 0.00 | 0.671 |
| 9/8/2021 | 145.30 | 0.37 | 0.00 | 0.672 |
| 9/9/2021 | 144.92 | 3.02 | 0.00 | 0.679 |
| 9/10/2021 | 141.90 | 3.90 | 0.00 | 0.687 |
| 9/11/2021 | 138.00 | 4.34 | 0.00 | 0.697 |
| 9/12/2021 | 133.66 | 2.94 | 0.00 | 0.704 |
| 9/15/2021 | 130.72 | 1.71 | 0.00 | 0.708 |
| 9/16/2021 | 129.02 | 5.66 | 0.00 | 0.721 |
| 9/17/2021 | 123.35 | 3.47 | 0.00 | 0.729 |
| 9/20/2021 | 119.88 | 8.18 | 0.00 | 0.747 |
| 9/22/2021 | 111.71 | 0.71 | 0.00 | 0.749 |
| 9/23/2021 | 111.00 | 3.20 | 0.00 | 0.756 |
| 9/25/2021 | 107.80 | 3.62 | 0.00 | 0.764 |
| 9/27/2021 | 104.18 | 1.42 | 0.00 | 0.767 |
| 9/28/2021 | 102.77 | 4.42 | 0.00 | 0.777 |
| 10/4/2021 | 98.35 | 1.38 | 0.00 | 0.780 |
| 10/7/2021 | 96.96 | 1.23 | 0.00 | 0.783 |
| 10/11/2021 | 95.74 | 0.28 | 0.00 | 0.784 |
| 10/14/2021 | 95.46 | 0.41 | 0.00 | 0.785 |
| 10/15/2021 | 95.05 | 0.99 | 0.00 | 0.787 |
| 10/16/2021 | 94.06 | 0.42 | 0.00 | 0.788 |
| 10/21/2021 | 93.64 | 0.18 | 0.00 | 0.788 |
| 10/25/2021 | 93.46 | 0.32 | 0.00 | 0.789 |
| 10/29/2021 | 93.15 | 0.49 | 0.00 | 0.790 |
| 11/3/2021 | 92.66 | 0.30 | 0.00 | 0.791 |
| 11/7/2021 | 92.35 | 0.18 | 0.00 | 0.791 |
| 11/12/2021 | 92.18 | 0.20 | 0.00 | 0.792 |
| 11/13/2021 | 91.98 | 0.22 | 0.00 | 0.792 |
| 11/14/2021 | 91.76 | 1.00 | 0.00 | 0.794 |
| 11/15/2021 | 90.76 | 0.31 | 0.00 | 0.795 |
| 12/3/2021 | 90.45 | 0.55 | 0.00 | 0.796 |
| 12/14/2021 | 89.90 | 0.17 | 0.00 | 0.797 |
| 12/15/2021 | 89.74 | 0.23 | 0.00 | 0.797 |
| 12/20/2021 | 89.51 | 0.23 | 0.00 | 0.798 |
| 12/22/2021 | 89.27 | 0.45 | 0.00 | 0.799 |
| 12/23/2021 | 88.82 | 2.16 | 0.00 | 0.804 |
| 12/29/2021 | 86.66 | 1.48 | 0.00 | 0.807 |
| 12/30/2021 | 85.18 | 0.24 | 0.00 | 0.808 |
| 1/3/2022 | 84.93 | 0.81 | 0.00 | 0.809 |
| 1/7/2022 | 84.13 | 1.22 | 0.00 | 0.812 |
| 1/12/2022 | 82.91 | 0.20 | 0.00 | 0.813 |
| 2/1/2022 | 82.71 | 0.09 | 0.00 | 0.813 |
| 3/10/2022 | 82.62 | 0.49 | 0.00 | 0.814 |
| 4/5/2022 | 82.13 | 0.44 | 0.00 | 0.815 |
| 4/10/2022 | 81.69 | 0.34 | 0.00 | 0.816 |
| 4/14/2022 | 81.35 | 0.67 | 0.00 | 0.817 |
| 5/6/2022 | 80.68 | 1.18 | 0.00 | 0.820 |
| 5/16/2022 | 79.50 | 0.33 | 0.00 | 0.821 |
| 5/27/2022 | 79.17 | 0.00 | 28.50 | 0.821 |
| 5/28/2022 | 50.66 | 0.00 | 15.95 | 0.821 |
| 5/29/2022 | 34.71 | 0.00 | 6.03 | 0.821 |
| 5/30/2022 | 28.68 | 0.00 | 4.25 | 0.821 |
| 5/31/2022 | 24.44 | 0.00 | 2.24 | 0.821 |
| 6/1/2022 | 22.19 | 0.00 | 1.70 | 0.821 |
| 6/2/2022 | 20.49 | 0.00 | 5.54 | 0.821 |
| 6/3/2022 | 14.95 | 0.00 | 2.03 | 0.821 |
| 6/4/2022 | 12.92 | 0.00 | 2.38 | 0.821 |
| 6/5/2022 | 10.54 | 0.00 | 0.24 | 0.821 |
| 6/7/2022 | 10.30 | 0.00 | 0.66 | 0.821 |
| 6/8/2022 | 9.64 | 0.00 | 0.37 | 0.821 |
| 6/10/2022 | 9.28 | 0.00 | 1.78 | 0.821 |
| 6/11/2022 | 7.49 | 0.00 | 3.25 | 0.821 |
| 6/12/2022 | 4.24 | 0.00 | 0.70 | 0.821 |
| 6/14/2022 | 3.54 | 0.00 | 0.14 | 0.821 |
| 6/15/2022 | 3.40 | 0.00 | 0.94 | 0.821 |
| 6/17/2022 | 2.46 | 0.00 | 0.65 | 0.821 |

| *Table S24. Skeptical Confidents Vaccine Uptake Survival Table* | | | | |
| --- | --- | --- | --- | --- |
| Time | At risk | Fail | Net lost | Failure function |
| 12/1/2020 | 0.00 | 0.00 | -726.39 | 0.000 |
| 12/6/2020 | 726.39 | 4.75 | 0.00 | 0.007 |
| 12/13/2020 | 721.64 | 1.16 | 0.00 | 0.008 |
| 12/18/2020 | 720.48 | 0.58 | 0.00 | 0.009 |
| 12/19/2020 | 719.90 | 0.48 | 0.00 | 0.010 |
| 12/21/2020 | 719.42 | 4.98 | 0.00 | 0.016 |
| 12/22/2020 | 714.43 | 0.62 | 0.00 | 0.017 |
| 12/23/2020 | 713.81 | 0.30 | 0.00 | 0.018 |
| 12/24/2020 | 713.51 | 0.67 | 0.00 | 0.019 |
| 12/26/2020 | 712.83 | 1.32 | 0.00 | 0.020 |
| 12/28/2020 | 711.51 | 0.85 | 0.00 | 0.022 |
| 12/30/2020 | 710.66 | 0.24 | 0.00 | 0.022 |
| 12/31/2020 | 710.42 | 2.21 | 0.00 | 0.025 |
| 1/2/2021 | 708.22 | 1.55 | 0.00 | 0.027 |
| 1/4/2021 | 706.67 | 0.65 | 0.00 | 0.028 |
| 1/5/2021 | 706.02 | 0.58 | 0.00 | 0.029 |
| 1/6/2021 | 705.44 | 2.38 | 0.00 | 0.032 |
| 1/7/2021 | 703.07 | 2.40 | 0.00 | 0.035 |
| 1/8/2021 | 700.67 | 0.43 | 0.00 | 0.036 |
| 1/10/2021 | 700.23 | 1.01 | 0.00 | 0.037 |
| 1/11/2021 | 699.23 | 2.57 | 0.00 | 0.041 |
| 1/12/2021 | 696.66 | 3.36 | 0.00 | 0.046 |
| 1/13/2021 | 693.30 | 1.39 | 0.00 | 0.047 |
| 1/14/2021 | 691.91 | 0.33 | 0.00 | 0.048 |
| 1/15/2021 | 691.58 | 3.64 | 0.00 | 0.053 |
| 1/16/2021 | 687.94 | 2.13 | 0.00 | 0.056 |
| 1/17/2021 | 685.81 | 1.34 | 0.00 | 0.058 |
| 1/19/2021 | 684.46 | 2.28 | 0.00 | 0.061 |
| 1/20/2021 | 682.19 | 4.68 | 0.00 | 0.067 |
| 1/21/2021 | 677.51 | 5.03 | 0.00 | 0.074 |
| 1/22/2021 | 672.48 | 3.21 | 0.00 | 0.079 |
| 1/23/2021 | 669.26 | 1.11 | 0.00 | 0.080 |
| 1/24/2021 | 668.16 | 1.36 | 0.00 | 0.082 |
| 1/25/2021 | 666.79 | 1.26 | 0.00 | 0.084 |
| 1/26/2021 | 665.53 | 1.73 | 0.00 | 0.086 |
| 1/27/2021 | 663.80 | 8.13 | 0.00 | 0.097 |
| 1/28/2021 | 655.67 | 4.81 | 0.00 | 0.104 |
| 1/29/2021 | 650.86 | 3.05 | 0.00 | 0.108 |
| 1/30/2021 | 647.81 | 2.15 | 0.00 | 0.111 |
| 2/1/2021 | 645.66 | 2.50 | 0.00 | 0.115 |
| 2/2/2021 | 643.16 | 5.63 | 0.00 | 0.122 |
| 2/3/2021 | 637.52 | 3.99 | 0.00 | 0.128 |
| 2/4/2021 | 633.54 | 1.27 | 0.00 | 0.130 |
| 2/5/2021 | 632.27 | 1.08 | 0.00 | 0.131 |
| 2/6/2021 | 631.19 | 3.80 | 0.00 | 0.136 |
| 2/7/2021 | 627.40 | 3.17 | 0.00 | 0.141 |
| 2/8/2021 | 624.22 | 3.32 | 0.00 | 0.145 |
| 2/9/2021 | 620.91 | 2.78 | 0.00 | 0.149 |
| 2/10/2021 | 618.12 | 5.59 | 0.00 | 0.157 |
| 2/11/2021 | 612.53 | 4.90 | 0.00 | 0.163 |
| 2/12/2021 | 607.63 | 5.82 | 0.00 | 0.172 |
| 2/13/2021 | 601.81 | 1.50 | 0.00 | 0.174 |
| 2/15/2021 | 600.31 | 5.45 | 0.00 | 0.181 |
| 2/16/2021 | 594.86 | 2.83 | 0.00 | 0.185 |
| 2/17/2021 | 592.04 | 4.61 | 0.00 | 0.191 |
| 2/18/2021 | 587.42 | 4.94 | 0.00 | 0.198 |
| 2/19/2021 | 582.49 | 4.87 | 0.00 | 0.205 |
| 2/20/2021 | 577.62 | 3.17 | 0.00 | 0.209 |
| 2/21/2021 | 574.45 | 2.33 | 0.00 | 0.212 |
| 2/22/2021 | 572.12 | 2.35 | 0.00 | 0.216 |
| 2/23/2021 | 569.77 | 4.04 | 0.00 | 0.221 |
| 2/24/2021 | 565.73 | 5.44 | 0.00 | 0.229 |
| 2/25/2021 | 560.29 | 13.97 | 0.00 | 0.248 |
| 2/26/2021 | 546.32 | 5.59 | 0.00 | 0.256 |
| 2/27/2021 | 540.73 | 5.45 | 0.00 | 0.263 |
| 2/28/2021 | 535.28 | 3.19 | 0.00 | 0.267 |
| 3/1/2021 | 532.09 | 5.01 | 0.00 | 0.274 |
| 3/2/2021 | 527.08 | 9.30 | 0.00 | 0.287 |
| 3/3/2021 | 517.78 | 4.38 | 0.00 | 0.293 |
| 3/4/2021 | 513.40 | 2.34 | 0.00 | 0.296 |
| 3/5/2021 | 511.06 | 9.71 | 0.00 | 0.310 |
| 3/6/2021 | 501.35 | 5.06 | 0.00 | 0.317 |
| 3/7/2021 | 496.29 | 2.83 | 0.00 | 0.321 |
| 3/8/2021 | 493.46 | 1.95 | 0.00 | 0.323 |
| 3/9/2021 | 491.51 | 3.76 | 0.00 | 0.329 |
| 3/10/2021 | 487.75 | 12.22 | 0.00 | 0.345 |
| 3/11/2021 | 475.54 | 10.86 | 0.00 | 0.360 |
| 3/12/2021 | 464.68 | 13.41 | 0.00 | 0.379 |
| 3/13/2021 | 451.27 | 7.37 | 0.00 | 0.389 |
| 3/14/2021 | 443.90 | 5.85 | 0.00 | 0.397 |
| 3/15/2021 | 438.06 | 11.55 | 0.00 | 0.413 |
| 3/16/2021 | 426.51 | 13.03 | 0.00 | 0.431 |
| 3/17/2021 | 413.48 | 4.90 | 0.00 | 0.438 |
| 3/18/2021 | 408.57 | 11.85 | 0.00 | 0.454 |
| 3/19/2021 | 396.72 | 11.97 | 0.00 | 0.470 |
| 3/20/2021 | 384.75 | 7.34 | 0.00 | 0.480 |
| 3/21/2021 | 377.41 | 6.55 | 0.00 | 0.489 |
| 3/22/2021 | 370.86 | 2.94 | 0.00 | 0.494 |
| 3/23/2021 | 367.92 | 11.06 | 0.00 | 0.509 |
| 3/24/2021 | 356.86 | 4.72 | 0.00 | 0.515 |
| 3/25/2021 | 352.13 | 3.39 | 0.00 | 0.520 |
| 3/26/2021 | 348.74 | 4.01 | 0.00 | 0.525 |
| 3/27/2021 | 344.73 | 2.58 | 0.00 | 0.529 |
| 3/28/2021 | 342.15 | 1.87 | 0.00 | 0.532 |
| 3/29/2021 | 340.28 | 4.52 | 0.00 | 0.538 |
| 3/30/2021 | 335.76 | 2.76 | 0.00 | 0.542 |
| 3/31/2021 | 333.00 | 2.86 | 0.00 | 0.545 |
| 4/1/2021 | 330.15 | 15.27 | 0.00 | 0.567 |
| 4/2/2021 | 314.88 | 3.75 | 0.00 | 0.572 |
| 4/3/2021 | 311.13 | 0.46 | 0.00 | 0.572 |
| 4/4/2021 | 310.67 | 9.17 | 0.00 | 0.585 |
| 4/5/2021 | 301.50 | 6.26 | 0.00 | 0.594 |
| 4/6/2021 | 295.25 | 4.75 | 0.00 | 0.600 |
| 4/7/2021 | 290.50 | 7.75 | 0.00 | 0.611 |
| 4/8/2021 | 282.75 | 5.78 | 0.00 | 0.619 |
| 4/9/2021 | 276.97 | 6.85 | 0.00 | 0.628 |
| 4/10/2021 | 270.11 | 8.37 | 0.00 | 0.640 |
| 4/11/2021 | 261.74 | 1.51 | 0.00 | 0.642 |
| 4/12/2021 | 260.22 | 2.44 | 0.00 | 0.645 |
| 4/13/2021 | 257.79 | 10.39 | 0.00 | 0.659 |
| 4/14/2021 | 247.40 | 4.05 | 0.00 | 0.665 |
| 4/15/2021 | 243.35 | 10.34 | 0.00 | 0.679 |
| 4/16/2021 | 233.01 | 6.81 | 0.00 | 0.689 |
| 4/17/2021 | 226.21 | 2.17 | 0.00 | 0.692 |
| 4/18/2021 | 224.03 | 2.27 | 0.00 | 0.695 |
| 4/19/2021 | 221.76 | 0.58 | 0.00 | 0.696 |
| 4/20/2021 | 221.18 | 9.91 | 0.00 | 0.709 |
| 4/21/2021 | 211.26 | 0.65 | 0.00 | 0.710 |
| 4/22/2021 | 210.62 | 2.75 | 0.00 | 0.714 |
| 4/23/2021 | 207.87 | 9.11 | 0.00 | 0.726 |
| 4/24/2021 | 198.76 | 2.41 | 0.00 | 0.730 |
| 4/25/2021 | 196.35 | 2.29 | 0.00 | 0.733 |
| 4/26/2021 | 194.06 | 4.69 | 0.00 | 0.739 |
| 4/27/2021 | 189.36 | 11.30 | 0.00 | 0.755 |
| 4/28/2021 | 178.06 | 1.01 | 0.00 | 0.756 |
| 4/29/2021 | 177.05 | 1.77 | 0.00 | 0.759 |
| 4/30/2021 | 175.28 | 8.41 | 0.00 | 0.770 |
| 5/1/2021 | 166.87 | 1.51 | 0.00 | 0.772 |
| 5/2/2021 | 165.36 | 1.82 | 0.00 | 0.775 |
| 5/3/2021 | 163.54 | 4.08 | 0.00 | 0.780 |
| 5/4/2021 | 159.46 | 1.19 | 0.00 | 0.782 |
| 5/5/2021 | 158.27 | 5.29 | 0.00 | 0.789 |
| 5/6/2021 | 152.98 | 2.65 | 0.00 | 0.793 |
| 5/7/2021 | 150.32 | 6.74 | 0.00 | 0.802 |
| 5/8/2021 | 143.59 | 1.36 | 0.00 | 0.804 |
| 5/10/2021 | 142.23 | 4.68 | 0.00 | 0.811 |
| 5/11/2021 | 137.55 | 2.41 | 0.00 | 0.814 |
| 5/12/2021 | 135.14 | 2.65 | 0.00 | 0.818 |
| 5/14/2021 | 132.49 | 1.24 | 0.00 | 0.819 |
| 5/15/2021 | 131.25 | 3.03 | 0.00 | 0.823 |
| 5/16/2021 | 128.22 | 1.18 | 0.00 | 0.825 |
| 5/18/2021 | 127.04 | 1.70 | 0.00 | 0.827 |
| 5/19/2021 | 125.35 | 2.38 | 0.00 | 0.831 |
| 5/20/2021 | 122.96 | 2.43 | 0.00 | 0.834 |
| 5/21/2021 | 120.53 | 4.24 | 0.00 | 0.840 |
| 5/22/2021 | 116.29 | 1.22 | 0.00 | 0.842 |
| 5/24/2021 | 115.07 | 0.31 | 0.00 | 0.842 |
| 5/25/2021 | 114.76 | 0.43 | 0.00 | 0.843 |
| 6/1/2021 | 114.33 | 3.35 | 0.00 | 0.847 |
| 6/3/2021 | 110.98 | 0.26 | 0.00 | 0.848 |
| 6/5/2021 | 110.72 | 1.51 | 0.00 | 0.850 |
| 6/7/2021 | 109.20 | 0.91 | 0.00 | 0.851 |
| 6/10/2021 | 108.29 | 0.16 | 0.00 | 0.851 |
| 6/14/2021 | 108.14 | 1.00 | 0.00 | 0.853 |
| 6/15/2021 | 107.14 | 2.53 | 0.00 | 0.856 |
| 6/18/2021 | 104.61 | 0.66 | 0.00 | 0.857 |
| 6/21/2021 | 103.95 | 0.53 | 0.00 | 0.858 |
| 6/22/2021 | 103.42 | 0.94 | 0.00 | 0.859 |
| 7/1/2021 | 102.49 | 1.30 | 0.00 | 0.861 |
| 7/2/2021 | 101.19 | 1.00 | 0.00 | 0.862 |
| 7/3/2021 | 100.19 | 3.19 | 0.00 | 0.866 |
| 7/4/2021 | 97.00 | 1.42 | 0.00 | 0.868 |
| 7/6/2021 | 95.57 | 0.82 | 0.00 | 0.870 |
| 7/7/2021 | 94.75 | 1.72 | 0.00 | 0.872 |
| 7/17/2021 | 93.03 | 0.58 | 0.00 | 0.873 |
| 7/18/2021 | 92.45 | 0.48 | 0.00 | 0.873 |
| 7/19/2021 | 91.97 | 3.33 | 0.00 | 0.878 |
| 7/28/2021 | 88.64 | 1.69 | 0.00 | 0.880 |
| 7/29/2021 | 86.95 | 0.53 | 0.00 | 0.881 |
| 8/4/2021 | 86.42 | 1.35 | 0.00 | 0.883 |
| 8/6/2021 | 85.06 | 4.89 | 0.00 | 0.890 |
| 8/7/2021 | 80.17 | 9.13 | 0.00 | 0.902 |
| 8/8/2021 | 71.03 | 0.47 | 0.00 | 0.903 |
| 8/10/2021 | 70.57 | 0.76 | 0.00 | 0.904 |
| 8/11/2021 | 69.80 | 1.96 | 0.00 | 0.907 |
| 8/13/2021 | 67.84 | 3.20 | 0.00 | 0.911 |
| 8/14/2021 | 64.64 | 1.42 | 0.00 | 0.913 |
| 8/19/2021 | 63.22 | 0.81 | 0.00 | 0.914 |
| 8/21/2021 | 62.41 | 3.07 | 0.00 | 0.918 |
| 8/23/2021 | 59.33 | 1.94 | 0.00 | 0.921 |
| 8/25/2021 | 57.39 | 0.34 | 0.00 | 0.921 |
| 9/3/2021 | 57.06 | 0.54 | 0.00 | 0.922 |
| 9/7/2021 | 56.51 | 1.27 | 0.00 | 0.924 |
| 9/10/2021 | 55.24 | 2.48 | 0.00 | 0.927 |
| 9/12/2021 | 52.76 | 2.07 | 0.00 | 0.930 |
| 9/21/2021 | 50.69 | 3.81 | 0.00 | 0.935 |
| 9/25/2021 | 46.88 | 0.34 | 0.00 | 0.936 |
| 10/2/2021 | 46.55 | 1.25 | 0.00 | 0.938 |
| 10/30/2021 | 45.30 | 7.54 | 0.00 | 0.948 |
| 11/16/2021 | 37.76 | 0.47 | 0.00 | 0.949 |
| 12/1/2021 | 37.28 | 0.85 | 0.00 | 0.950 |
| 1/7/2022 | 36.44 | 0.24 | 0.00 | 0.950 |
| 1/11/2022 | 36.19 | 0.95 | 0.00 | 0.951 |
| 1/17/2022 | 35.25 | 0.18 | 0.00 | 0.952 |
| 1/21/2022 | 35.06 | 0.38 | 0.00 | 0.952 |
| 1/24/2022 | 34.68 | 0.39 | 0.00 | 0.953 |
| 2/9/2022 | 34.29 | 0.46 | 0.00 | 0.953 |
| 2/28/2022 | 33.83 | 1.12 | 0.00 | 0.955 |
| 3/28/2022 | 32.71 | 0.61 | 0.00 | 0.956 |
| 4/3/2022 | 32.10 | 0.62 | 0.00 | 0.957 |
| 5/20/2022 | 31.48 | 0.71 | 0.00 | 0.958 |
| 5/27/2022 | 30.77 | 0.00 | 6.57 | 0.958 |
| 5/28/2022 | 24.20 | 0.00 | 5.29 | 0.958 |
| 5/29/2022 | 18.91 | 0.00 | 1.23 | 0.958 |
| 5/30/2022 | 17.68 | 0.00 | 6.15 | 0.958 |
| 5/31/2022 | 11.53 | 0.00 | 1.85 | 0.958 |
| 6/1/2022 | 9.68 | 0.00 | 5.44 | 0.958 |
| 6/3/2022 | 4.24 | 0.00 | 0.96 | 0.958 |
| 6/21/2022 | 3.28 | 0.00 | 1.24 | 0.958 |
| 6/25/2022 | 2.04 | 0.00 | 0.37 | 0.958 |

| *Table S25. Ready Confidents Vaccine Uptake Survival Table* | | | | |
| --- | --- | --- | --- | --- |
| Time | At risk | Fail | Net lost | Failure function |
| 12/1/2020 | 0.00 | 0.00 | -1152.40 | 0.000 |
| 12/2/2020 | 1152.40 | 1.21 | 0.00 | 0.001 |
| 12/10/2020 | 1151.19 | 0.47 | 0.00 | 0.001 |
| 12/17/2020 | 1150.71 | 2.28 | 0.00 | 0.003 |
| 12/18/2020 | 1148.43 | 1.18 | 0.00 | 0.004 |
| 12/19/2020 | 1147.25 | 1.23 | 0.00 | 0.006 |
| 12/20/2020 | 1146.02 | 1.80 | 0.00 | 0.007 |
| 12/21/2020 | 1144.22 | 3.15 | 0.00 | 0.010 |
| 12/22/2020 | 1141.07 | 2.02 | 0.00 | 0.012 |
| 12/23/2020 | 1139.05 | 2.78 | 0.00 | 0.014 |
| 12/24/2020 | 1136.27 | 1.31 | 0.00 | 0.015 |
| 12/27/2020 | 1134.96 | 1.82 | 0.00 | 0.017 |
| 12/28/2020 | 1133.14 | 1.33 | 0.00 | 0.018 |
| 12/29/2020 | 1131.81 | 7.10 | 0.00 | 0.024 |
| 12/30/2020 | 1124.71 | 0.98 | 0.00 | 0.025 |
| 12/31/2020 | 1123.73 | 1.72 | 0.00 | 0.026 |
| 1/1/2021 | 1122.00 | 0.58 | 0.00 | 0.027 |
| 1/2/2021 | 1121.42 | 2.48 | 0.00 | 0.029 |
| 1/3/2021 | 1118.94 | 3.90 | 0.00 | 0.032 |
| 1/4/2021 | 1115.04 | 5.85 | 0.00 | 0.037 |
| 1/5/2021 | 1109.19 | 8.16 | 0.00 | 0.045 |
| 1/6/2021 | 1101.03 | 4.83 | 0.00 | 0.049 |
| 1/7/2021 | 1096.19 | 2.68 | 0.00 | 0.051 |
| 1/8/2021 | 1093.51 | 2.74 | 0.00 | 0.053 |
| 1/9/2021 | 1090.77 | 3.74 | 0.00 | 0.057 |
| 1/10/2021 | 1087.03 | 0.84 | 0.00 | 0.057 |
| 1/11/2021 | 1086.20 | 3.90 | 0.00 | 0.061 |
| 1/12/2021 | 1082.29 | 3.91 | 0.00 | 0.064 |
| 1/13/2021 | 1078.38 | 5.40 | 0.00 | 0.069 |
| 1/14/2021 | 1072.98 | 6.63 | 0.00 | 0.075 |
| 1/15/2021 | 1066.35 | 11.83 | 0.00 | 0.085 |
| 1/16/2021 | 1054.52 | 5.69 | 0.00 | 0.090 |
| 1/17/2021 | 1048.83 | 3.09 | 0.00 | 0.093 |
| 1/18/2021 | 1045.74 | 1.51 | 0.00 | 0.094 |
| 1/19/2021 | 1044.23 | 5.04 | 0.00 | 0.098 |
| 1/20/2021 | 1039.20 | 12.14 | 0.00 | 0.109 |
| 1/21/2021 | 1027.06 | 6.70 | 0.00 | 0.115 |
| 1/22/2021 | 1020.36 | 4.62 | 0.00 | 0.119 |
| 1/23/2021 | 1015.75 | 5.63 | 0.00 | 0.123 |
| 1/24/2021 | 1010.12 | 3.23 | 0.00 | 0.126 |
| 1/25/2021 | 1006.88 | 1.97 | 0.00 | 0.128 |
| 1/26/2021 | 1004.91 | 8.85 | 0.00 | 0.136 |
| 1/27/2021 | 996.06 | 18.66 | 0.00 | 0.152 |
| 1/28/2021 | 977.40 | 12.26 | 0.00 | 0.162 |
| 1/29/2021 | 965.14 | 11.67 | 0.00 | 0.173 |
| 1/30/2021 | 953.47 | 4.62 | 0.00 | 0.177 |
| 1/31/2021 | 948.85 | 1.42 | 0.00 | 0.178 |
| 2/1/2021 | 947.43 | 6.98 | 0.00 | 0.184 |
| 2/2/2021 | 940.45 | 10.93 | 0.00 | 0.193 |
| 2/3/2021 | 929.52 | 9.47 | 0.00 | 0.202 |
| 2/4/2021 | 920.04 | 11.84 | 0.00 | 0.212 |
| 2/5/2021 | 908.20 | 10.67 | 0.00 | 0.221 |
| 2/6/2021 | 897.54 | 6.63 | 0.00 | 0.227 |
| 2/7/2021 | 890.91 | 4.29 | 0.00 | 0.231 |
| 2/8/2021 | 886.62 | 9.13 | 0.00 | 0.239 |
| 2/9/2021 | 877.49 | 3.80 | 0.00 | 0.242 |
| 2/10/2021 | 873.69 | 13.75 | 0.00 | 0.254 |
| 2/11/2021 | 859.94 | 12.46 | 0.00 | 0.265 |
| 2/12/2021 | 847.47 | 8.42 | 0.00 | 0.272 |
| 2/13/2021 | 839.06 | 9.43 | 0.00 | 0.280 |
| 2/14/2021 | 829.63 | 7.92 | 0.00 | 0.287 |
| 2/15/2021 | 821.70 | 5.19 | 0.00 | 0.291 |
| 2/16/2021 | 816.51 | 10.41 | 0.00 | 0.300 |
| 2/17/2021 | 806.10 | 9.11 | 0.00 | 0.308 |
| 2/18/2021 | 796.99 | 6.06 | 0.00 | 0.314 |
| 2/19/2021 | 790.92 | 3.59 | 0.00 | 0.317 |
| 2/20/2021 | 787.33 | 0.78 | 0.00 | 0.317 |
| 2/21/2021 | 786.55 | 5.01 | 0.00 | 0.322 |
| 2/22/2021 | 781.55 | 3.53 | 0.00 | 0.325 |
| 2/23/2021 | 778.02 | 7.59 | 0.00 | 0.331 |
| 2/24/2021 | 770.43 | 3.75 | 0.00 | 0.335 |
| 2/25/2021 | 766.68 | 15.46 | 0.00 | 0.348 |
| 2/26/2021 | 751.22 | 11.32 | 0.00 | 0.358 |
| 2/27/2021 | 739.90 | 6.78 | 0.00 | 0.364 |
| 2/28/2021 | 733.13 | 5.75 | 0.00 | 0.369 |
| 3/1/2021 | 727.38 | 13.98 | 0.00 | 0.381 |
| 3/2/2021 | 713.41 | 3.72 | 0.00 | 0.384 |
| 3/3/2021 | 709.68 | 16.87 | 0.00 | 0.399 |
| 3/4/2021 | 692.81 | 15.95 | 0.00 | 0.413 |
| 3/5/2021 | 676.87 | 15.04 | 0.00 | 0.426 |
| 3/6/2021 | 661.82 | 14.76 | 0.00 | 0.439 |
| 3/7/2021 | 647.06 | 18.47 | 0.00 | 0.455 |
| 3/8/2021 | 628.59 | 11.53 | 0.00 | 0.465 |
| 3/9/2021 | 617.06 | 9.50 | 0.00 | 0.473 |
| 3/10/2021 | 607.56 | 17.95 | 0.00 | 0.488 |
| 3/11/2021 | 589.61 | 11.27 | 0.00 | 0.498 |
| 3/12/2021 | 578.34 | 10.83 | 0.00 | 0.508 |
| 3/13/2021 | 567.51 | 7.36 | 0.00 | 0.514 |
| 3/14/2021 | 560.15 | 20.87 | 0.00 | 0.532 |
| 3/15/2021 | 539.29 | 21.72 | 0.00 | 0.551 |
| 3/16/2021 | 517.57 | 8.80 | 0.00 | 0.559 |
| 3/17/2021 | 508.77 | 10.41 | 0.00 | 0.568 |
| 3/18/2021 | 498.36 | 13.75 | 0.00 | 0.579 |
| 3/19/2021 | 484.61 | 14.73 | 0.00 | 0.592 |
| 3/20/2021 | 469.88 | 9.30 | 0.00 | 0.600 |
| 3/21/2021 | 460.58 | 9.14 | 0.00 | 0.608 |
| 3/22/2021 | 451.44 | 10.60 | 0.00 | 0.617 |
| 3/23/2021 | 440.84 | 9.76 | 0.00 | 0.626 |
| 3/24/2021 | 431.08 | 16.38 | 0.00 | 0.640 |
| 3/25/2021 | 414.70 | 15.55 | 0.00 | 0.654 |
| 3/26/2021 | 399.15 | 7.59 | 0.00 | 0.660 |
| 3/27/2021 | 391.55 | 14.69 | 0.00 | 0.673 |
| 3/28/2021 | 376.86 | 2.50 | 0.00 | 0.675 |
| 3/29/2021 | 374.36 | 9.30 | 0.00 | 0.683 |
| 3/30/2021 | 365.06 | 12.29 | 0.00 | 0.694 |
| 3/31/2021 | 352.76 | 12.42 | 0.00 | 0.705 |
| 4/1/2021 | 340.35 | 17.13 | 0.00 | 0.720 |
| 4/2/2021 | 323.22 | 9.56 | 0.00 | 0.728 |
| 4/3/2021 | 313.66 | 5.19 | 0.00 | 0.732 |
| 4/4/2021 | 308.46 | 7.99 | 0.00 | 0.739 |
| 4/5/2021 | 300.47 | 11.65 | 0.00 | 0.749 |
| 4/6/2021 | 288.82 | 11.51 | 0.00 | 0.759 |
| 4/7/2021 | 277.32 | 15.24 | 0.00 | 0.773 |
| 4/8/2021 | 262.08 | 10.99 | 0.00 | 0.782 |
| 4/9/2021 | 251.09 | 8.28 | 0.00 | 0.789 |
| 4/10/2021 | 242.81 | 17.10 | 0.00 | 0.804 |
| 4/11/2021 | 225.71 | 3.52 | 0.00 | 0.807 |
| 4/12/2021 | 222.19 | 8.07 | 0.00 | 0.814 |
| 4/13/2021 | 214.12 | 2.64 | 0.00 | 0.816 |
| 4/14/2021 | 211.47 | 14.94 | 0.00 | 0.829 |
| 4/15/2021 | 196.53 | 13.92 | 0.00 | 0.842 |
| 4/16/2021 | 182.62 | 5.88 | 0.00 | 0.847 |
| 4/17/2021 | 176.74 | 2.62 | 0.00 | 0.849 |
| 4/18/2021 | 174.12 | 5.75 | 0.00 | 0.854 |
| 4/19/2021 | 168.37 | 2.96 | 0.00 | 0.856 |
| 4/20/2021 | 165.41 | 9.55 | 0.00 | 0.865 |
| 4/21/2021 | 155.86 | 3.15 | 0.00 | 0.867 |
| 4/22/2021 | 152.71 | 2.58 | 0.00 | 0.870 |
| 4/23/2021 | 150.13 | 9.30 | 0.00 | 0.878 |
| 4/24/2021 | 140.83 | 1.28 | 0.00 | 0.879 |
| 4/25/2021 | 139.55 | 2.29 | 0.00 | 0.881 |
| 4/26/2021 | 137.26 | 2.18 | 0.00 | 0.883 |
| 4/27/2021 | 135.08 | 1.04 | 0.00 | 0.884 |
| 4/28/2021 | 134.04 | 2.71 | 0.00 | 0.886 |
| 4/29/2021 | 131.33 | 1.81 | 0.00 | 0.888 |
| 4/30/2021 | 129.52 | 9.59 | 0.00 | 0.896 |
| 5/1/2021 | 119.93 | 8.62 | 0.00 | 0.903 |
| 5/2/2021 | 111.32 | 3.21 | 0.00 | 0.906 |
| 5/3/2021 | 108.11 | 0.75 | 0.00 | 0.907 |
| 5/4/2021 | 107.36 | 1.12 | 0.00 | 0.908 |
| 5/5/2021 | 106.24 | 7.92 | 0.00 | 0.915 |
| 5/6/2021 | 98.32 | 5.26 | 0.00 | 0.919 |
| 5/7/2021 | 93.06 | 2.18 | 0.00 | 0.921 |
| 5/8/2021 | 90.88 | 0.74 | 0.00 | 0.922 |
| 5/9/2021 | 90.14 | 0.85 | 0.00 | 0.923 |
| 5/10/2021 | 89.29 | 3.27 | 0.00 | 0.925 |
| 5/11/2021 | 86.02 | 4.74 | 0.00 | 0.929 |
| 5/12/2021 | 81.28 | 0.52 | 0.00 | 0.930 |
| 5/13/2021 | 80.76 | 0.41 | 0.00 | 0.930 |
| 5/14/2021 | 80.35 | 3.34 | 0.00 | 0.933 |
| 5/15/2021 | 77.01 | 9.10 | 0.00 | 0.941 |
| 5/16/2021 | 67.91 | 3.34 | 0.00 | 0.944 |
| 5/17/2021 | 64.57 | 0.83 | 0.00 | 0.945 |
| 5/18/2021 | 63.74 | 2.34 | 0.00 | 0.947 |
| 5/19/2021 | 61.41 | 0.21 | 0.00 | 0.947 |
| 5/20/2021 | 61.19 | 2.26 | 0.00 | 0.949 |
| 5/21/2021 | 58.93 | 0.47 | 0.00 | 0.949 |
| 5/22/2021 | 58.46 | 0.23 | 0.00 | 0.949 |
| 5/23/2021 | 58.23 | 2.06 | 0.00 | 0.951 |
| 6/1/2021 | 56.17 | 4.25 | 0.00 | 0.955 |
| 6/3/2021 | 51.92 | 1.00 | 0.00 | 0.956 |
| 6/4/2021 | 50.92 | 0.95 | 0.00 | 0.957 |
| 6/5/2021 | 49.97 | 1.95 | 0.00 | 0.958 |
| 6/6/2021 | 48.02 | 0.41 | 0.00 | 0.959 |
| 6/20/2021 | 47.61 | 0.72 | 0.00 | 0.959 |
| 7/2/2021 | 46.89 | 1.42 | 0.00 | 0.961 |
| 7/3/2021 | 45.47 | 1.19 | 0.00 | 0.962 |
| 7/12/2021 | 44.28 | 5.50 | 0.00 | 0.966 |
| 7/14/2021 | 38.79 | 2.15 | 0.00 | 0.968 |
| 7/18/2021 | 36.64 | 0.25 | 0.00 | 0.968 |
| 7/20/2021 | 36.39 | 1.83 | 0.00 | 0.970 |
| 7/21/2021 | 34.56 | 1.09 | 0.00 | 0.971 |
| 7/23/2021 | 33.47 | 1.10 | 0.00 | 0.972 |
| 7/24/2021 | 32.37 | 1.02 | 0.00 | 0.973 |
| 7/26/2021 | 31.35 | 0.52 | 0.00 | 0.973 |
| 7/28/2021 | 30.83 | 0.83 | 0.00 | 0.974 |
| 8/6/2021 | 30.01 | 0.33 | 0.00 | 0.974 |
| 8/21/2021 | 29.67 | 5.05 | 0.00 | 0.979 |
| 8/25/2021 | 24.62 | 0.42 | 0.00 | 0.979 |
| 9/1/2021 | 24.20 | 1.97 | 0.00 | 0.981 |
| 9/2/2021 | 22.23 | 0.76 | 0.00 | 0.981 |
| 9/10/2021 | 21.47 | 4.14 | 0.00 | 0.985 |
| 9/15/2021 | 17.32 | 2.85 | 0.00 | 0.987 |
| 9/22/2021 | 14.47 | 4.73 | 0.00 | 0.992 |
| 10/14/2021 | 9.74 | 0.34 | 0.00 | 0.992 |
| 11/10/2021 | 9.40 | 1.07 | 0.00 | 0.993 |
| 11/29/2021 | 8.33 | 0.36 | 0.00 | 0.993 |
| 5/26/2022 | 7.97 | 0.00 | 2.79 | 0.993 |
| 5/27/2022 | 5.17 | 0.00 | 2.12 | 0.993 |
| 5/28/2022 | 3.05 | 0.00 | 1.53 | 0.993 |

| *Table S26. Hardline Non-Intender Booster Uptake Survival Table* | | | | |
| --- | --- | --- | --- | --- |
| Time | At risk | Fail | Net lost | Failure function |
| 8/1/2021 | 0.00 | 0.00 | -32.31 | 0.000 |
| 1/10/2022 | 32.31 | 0.87 | 0.00 | 0.027 |
| 3/23/2022 | 31.44 | 0.98 | 0.00 | 0.057 |
| 5/26/2022 | 30.46 | 0.00 | 0.75 | 0.057 |
| 5/27/2022 | 29.72 | 0.00 | 6.13 | 0.057 |
| 5/28/2022 | 23.58 | 0.00 | 3.41 | 0.057 |
| 5/29/2022 | 20.18 | 0.00 | 6.50 | 0.057 |
| 5/30/2022 | 13.68 | 0.00 | 1.29 | 0.057 |
| 5/31/2022 | 12.39 | 0.00 | 5.58 | 0.057 |
| 6/1/2022 | 6.81 | 0.00 | 5.75 | 0.057 |
| 6/7/2022 | 1.06 | 0.00 | 1.06 | 0.057 |

| *Table S27. Prevention-Compliant Non-Intender Booster Uptake Survival Table* | | | | |
| --- | --- | --- | --- | --- |
| Time | At risk | Fail | Net lost | Failure function |
| 8/1/2021 | 0.00 | 0.00 | -65.28 | 0.000 |
| 10/28/2021 | 65.28 | 0.43 | 0.00 | 0.007 |
| 12/5/2021 | 64.85 | 1.71 | 0.00 | 0.033 |
| 12/20/2021 | 63.14 | 1.68 | 0.00 | 0.059 |
| 1/18/2022 | 61.46 | 0.50 | 0.00 | 0.066 |
| 1/23/2022 | 60.96 | 0.97 | 0.00 | 0.081 |
| 2/9/2022 | 60.00 | 0.14 | 0.00 | 0.083 |
| 3/15/2022 | 59.86 | 1.12 | 0.00 | 0.100 |
| 5/27/2022 | 58.74 | 0.00 | 6.32 | 0.100 |
| 5/28/2022 | 52.42 | 0.00 | 13.21 | 0.100 |
| 5/29/2022 | 39.22 | 0.00 | 2.21 | 0.100 |
| 5/30/2022 | 37.01 | 0.00 | 1.19 | 0.100 |
| 5/31/2022 | 35.82 | 0.00 | 4.16 | 0.100 |
| 6/1/2022 | 31.65 | 0.00 | 3.36 | 0.100 |
| 6/2/2022 | 28.30 | 0.00 | 1.87 | 0.100 |
| 6/4/2022 | 26.43 | 0.00 | 2.08 | 0.100 |
| 6/7/2022 | 24.35 | 0.00 | 7.90 | 0.100 |
| 6/8/2022 | 16.45 | 0.00 | 0.92 | 0.100 |
| 6/9/2022 | 15.53 | 0.00 | 0.63 | 0.100 |
| 6/11/2022 | 14.90 | 0.00 | 6.28 | 0.100 |
| 6/17/2022 | 8.62 | 0.00 | 2.16 | 0.100 |
| 6/20/2022 | 6.46 | 0.00 | 1.07 | 0.100 |
| 6/23/2022 | 5.39 | 0.00 | 3.18 | 0.100 |
| 6/24/2022 | 2.21 | 0.00 | 1.68 | 0.100 |
| 6/28/2022 | 0.53 | 0.00 | 0.53 | 0.100 |

| *Table S28. Burned Out Waiter Booster Uptake Survival Table* | | | | |
| --- | --- | --- | --- | --- |
| Time | At risk | Fail | Net lost | Failure function |
| 8/1/2021 | 0.00 | 0.00 | -166.36 | 0.000 |
| 9/9/2021 | 166.36 | 1.93 | 0.00 | 0.012 |
| 10/10/2021 | 164.43 | 0.49 | 0.00 | 0.015 |
| 10/15/2021 | 163.93 | 0.47 | 0.00 | 0.017 |
| 10/25/2021 | 163.46 | 1.24 | 0.00 | 0.025 |
| 10/29/2021 | 162.22 | 1.14 | 0.00 | 0.032 |
| 11/1/2021 | 161.08 | 0.59 | 0.00 | 0.035 |
| 11/6/2021 | 160.49 | 0.53 | 0.00 | 0.038 |
| 11/8/2021 | 159.96 | 0.12 | 0.00 | 0.039 |
| 11/11/2021 | 159.84 | 0.44 | 0.00 | 0.042 |
| 11/12/2021 | 159.40 | 0.81 | 0.00 | 0.047 |
| 11/15/2021 | 158.59 | 0.36 | 0.00 | 0.049 |
| 11/17/2021 | 158.24 | 0.88 | 0.00 | 0.054 |
| 11/20/2021 | 157.36 | 0.83 | 0.00 | 0.059 |
| 11/21/2021 | 156.53 | 0.80 | 0.00 | 0.064 |
| 11/23/2021 | 155.73 | 1.00 | 0.00 | 0.070 |
| 11/26/2021 | 154.73 | 0.32 | 0.00 | 0.072 |
| 12/5/2021 | 154.41 | 1.79 | 0.00 | 0.083 |
| 12/9/2021 | 152.62 | 0.19 | 0.00 | 0.084 |
| 12/10/2021 | 152.43 | 2.83 | 0.00 | 0.101 |
| 12/11/2021 | 149.59 | 0.30 | 0.00 | 0.103 |
| 12/19/2021 | 149.30 | 0.95 | 0.00 | 0.108 |
| 12/31/2021 | 148.34 | 0.37 | 0.00 | 0.111 |
| 1/4/2022 | 147.97 | 0.29 | 0.00 | 0.112 |
| 1/5/2022 | 147.68 | 0.67 | 0.00 | 0.116 |
| 1/7/2022 | 147.01 | 0.84 | 0.00 | 0.121 |
| 1/21/2022 | 146.17 | 0.47 | 0.00 | 0.124 |
| 1/24/2022 | 145.70 | 0.30 | 0.00 | 0.126 |
| 2/1/2022 | 145.40 | 1.79 | 0.00 | 0.137 |
| 2/4/2022 | 143.60 | 0.54 | 0.00 | 0.140 |
| 2/8/2022 | 143.07 | 0.82 | 0.00 | 0.145 |
| 2/15/2022 | 142.24 | 0.32 | 0.00 | 0.147 |
| 2/24/2022 | 141.92 | 0.31 | 0.00 | 0.149 |
| 3/2/2022 | 141.61 | 3.44 | 0.00 | 0.169 |
| 5/26/2022 | 138.17 | 0.00 | 3.92 | 0.169 |
| 5/27/2022 | 134.25 | 0.00 | 58.79 | 0.169 |
| 5/28/2022 | 75.47 | 0.00 | 27.87 | 0.169 |
| 5/29/2022 | 47.60 | 0.00 | 3.07 | 0.169 |
| 5/30/2022 | 44.53 | 0.00 | 7.57 | 0.169 |
| 5/31/2022 | 36.95 | 0.00 | 3.53 | 0.169 |
| 6/1/2022 | 33.42 | 0.00 | 4.70 | 0.169 |
| 6/2/2022 | 28.73 | 0.00 | 6.49 | 0.169 |
| 6/3/2022 | 22.23 | 0.00 | 1.23 | 0.169 |
| 6/6/2022 | 21.00 | 0.00 | 6.22 | 0.169 |
| 6/12/2022 | 14.78 | 0.00 | 0.88 | 0.169 |
| 6/17/2022 | 13.90 | 0.00 | 8.02 | 0.169 |
| 6/18/2022 | 5.88 | 0.00 | 0.66 | 0.169 |
| 6/21/2022 | 5.22 | 0.00 | 1.65 | 0.169 |
| 6/22/2022 | 3.58 | 0.00 | 1.93 | 0.169 |
| 6/24/2022 | 1.64 | 0.00 | 0.68 | 0.169 |
| 6/27/2022 | 0.96 | 0.00 | 0.76 | 0.169 |
| 7/5/2022 | 0.20 | 0.00 | 0.20 | 0.169 |

| *Table S29. Anxious Waiter Booster Uptake Survival Table* | | | | |
| --- | --- | --- | --- | --- |
| Time | At risk | Fail | Net lost | Failure function |
| 8/1/2021 | 0.00 | 0.00 | -290.12 | 0.000 |
| 8/20/2021 | 290.12 | 0.34 | 0.00 | 0.001 |
| 9/30/2021 | 289.77 | 1.45 | 0.00 | 0.006 |
| 10/6/2021 | 288.32 | 0.35 | 0.00 | 0.007 |
| 10/8/2021 | 287.98 | 0.33 | 0.00 | 0.009 |
| 10/11/2021 | 287.65 | 0.44 | 0.00 | 0.010 |
| 10/14/2021 | 287.21 | 0.59 | 0.00 | 0.012 |
| 10/15/2021 | 286.62 | 0.51 | 0.00 | 0.014 |
| 10/18/2021 | 286.11 | 0.40 | 0.00 | 0.015 |
| 10/25/2021 | 285.71 | 0.96 | 0.00 | 0.018 |
| 10/26/2021 | 284.75 | 0.73 | 0.00 | 0.021 |
| 10/28/2021 | 284.03 | 1.16 | 0.00 | 0.025 |
| 10/29/2021 | 282.87 | 0.37 | 0.00 | 0.026 |
| 11/1/2021 | 282.49 | 0.47 | 0.00 | 0.028 |
| 11/7/2021 | 282.03 | 0.48 | 0.00 | 0.030 |
| 11/8/2021 | 281.55 | 0.47 | 0.00 | 0.031 |
| 11/10/2021 | 281.08 | 0.89 | 0.00 | 0.034 |
| 11/11/2021 | 280.19 | 0.39 | 0.00 | 0.036 |
| 11/12/2021 | 279.81 | 1.36 | 0.00 | 0.040 |
| 11/13/2021 | 278.45 | 0.42 | 0.00 | 0.042 |
| 11/14/2021 | 278.04 | 1.38 | 0.00 | 0.046 |
| 11/15/2021 | 276.65 | 1.12 | 0.00 | 0.050 |
| 11/20/2021 | 275.54 | 2.11 | 0.00 | 0.058 |
| 11/22/2021 | 273.43 | 1.62 | 0.00 | 0.063 |
| 11/24/2021 | 271.80 | 0.33 | 0.00 | 0.064 |
| 11/26/2021 | 271.48 | 0.39 | 0.00 | 0.066 |
| 12/1/2021 | 271.09 | 1.52 | 0.00 | 0.071 |
| 12/3/2021 | 269.56 | 0.44 | 0.00 | 0.072 |
| 12/5/2021 | 269.12 | 0.91 | 0.00 | 0.075 |
| 12/6/2021 | 268.21 | 0.32 | 0.00 | 0.077 |
| 12/8/2021 | 267.89 | 0.42 | 0.00 | 0.078 |
| 12/9/2021 | 267.47 | 0.43 | 0.00 | 0.080 |
| 12/10/2021 | 267.05 | 1.73 | 0.00 | 0.085 |
| 12/15/2021 | 265.32 | 0.76 | 0.00 | 0.088 |
| 12/16/2021 | 264.56 | 1.11 | 0.00 | 0.092 |
| 12/17/2021 | 263.45 | 0.81 | 0.00 | 0.095 |
| 12/18/2021 | 262.64 | 0.68 | 0.00 | 0.097 |
| 12/21/2021 | 261.96 | 1.51 | 0.00 | 0.102 |
| 12/22/2021 | 260.45 | 0.34 | 0.00 | 0.103 |
| 12/30/2021 | 260.11 | 0.97 | 0.00 | 0.107 |
| 1/2/2022 | 259.13 | 0.16 | 0.00 | 0.107 |
| 1/13/2022 | 258.98 | 0.68 | 0.00 | 0.110 |
| 1/14/2022 | 258.29 | 0.56 | 0.00 | 0.112 |
| 1/15/2022 | 257.73 | 1.17 | 0.00 | 0.116 |
| 1/21/2022 | 256.56 | 0.63 | 0.00 | 0.118 |
| 1/24/2022 | 255.93 | 0.35 | 0.00 | 0.119 |
| 1/27/2022 | 255.57 | 0.36 | 0.00 | 0.120 |
| 1/31/2022 | 255.21 | 0.54 | 0.00 | 0.122 |
| 2/5/2022 | 254.67 | 1.33 | 0.00 | 0.127 |
| 2/8/2022 | 253.34 | 0.62 | 0.00 | 0.129 |
| 2/10/2022 | 252.72 | 1.61 | 0.00 | 0.134 |
| 2/18/2022 | 251.10 | 1.43 | 0.00 | 0.139 |
| 2/27/2022 | 249.67 | 1.18 | 0.00 | 0.143 |
| 2/28/2022 | 248.49 | 0.50 | 0.00 | 0.145 |
| 3/4/2022 | 247.99 | 0.38 | 0.00 | 0.147 |
| 3/10/2022 | 247.61 | 0.76 | 0.00 | 0.149 |
| 3/11/2022 | 246.85 | 1.95 | 0.00 | 0.156 |
| 3/15/2022 | 244.90 | 0.41 | 0.00 | 0.157 |
| 4/4/2022 | 244.49 | 0.77 | 0.00 | 0.160 |
| 4/8/2022 | 243.72 | 0.45 | 0.00 | 0.161 |
| 4/10/2022 | 243.27 | 0.51 | 0.00 | 0.163 |
| 4/11/2022 | 242.76 | 0.68 | 0.00 | 0.166 |
| 4/20/2022 | 242.08 | 2.21 | 0.00 | 0.173 |
| 5/14/2022 | 239.87 | 1.82 | 0.00 | 0.179 |
| 5/17/2022 | 238.05 | 0.16 | 0.00 | 0.180 |
| 5/20/2022 | 237.89 | 0.48 | 0.00 | 0.182 |
| 5/22/2022 | 237.42 | 0.22 | 0.00 | 0.182 |
| 5/26/2022 | 237.19 | 0.00 | 15.76 | 0.182 |
| 5/27/2022 | 221.44 | 0.00 | 62.77 | 0.182 |
| 5/28/2022 | 158.66 | 0.00 | 25.91 | 0.182 |
| 5/29/2022 | 132.75 | 0.00 | 24.01 | 0.182 |
| 5/30/2022 | 108.75 | 0.00 | 27.43 | 0.182 |
| 5/31/2022 | 81.32 | 0.00 | 14.39 | 0.182 |
| 6/1/2022 | 66.93 | 0.00 | 18.67 | 0.182 |
| 6/2/2022 | 48.27 | 0.00 | 0.85 | 0.182 |
| 6/4/2022 | 47.41 | 0.00 | 2.81 | 0.182 |
| 6/5/2022 | 44.60 | 0.00 | 7.75 | 0.182 |
| 6/6/2022 | 36.85 | 0.00 | 4.21 | 0.182 |
| 6/7/2022 | 32.64 | 0.00 | 2.37 | 0.182 |
| 6/8/2022 | 30.27 | 0.00 | 1.71 | 0.182 |
| 6/10/2022 | 28.56 | 0.00 | 5.06 | 0.182 |
| 6/12/2022 | 23.49 | 0.00 | 2.29 | 0.182 |
| 6/13/2022 | 21.21 | 0.00 | 8.50 | 0.182 |
| 6/15/2022 | 12.71 | 0.00 | 2.33 | 0.182 |
| 6/18/2022 | 10.38 | 0.00 | 1.12 | 0.182 |
| 6/20/2022 | 9.26 | 0.00 | 3.10 | 0.182 |
| 6/21/2022 | 6.16 | 0.00 | 3.11 | 0.182 |
| 6/23/2022 | 3.05 | 0.00 | 2.08 | 0.182 |
| 6/27/2022 | 0.97 | 0.00 | 0.97 | 0.182 |

| *Table S30. Skeptical Confidents Booster Uptake Survival Table* | | | | |
| --- | --- | --- | --- | --- |
| Time | At risk | Fail | Net lost | Failure function |
| 8/1/2021 | 0.00 | 0.00 | -484.69 | 0.000 |
| 9/24/2021 | 484.69 | 0.31 | 0.00 | 0.001 |
| 9/26/2021 | 484.38 | 0.78 | 0.00 | 0.002 |
| 9/27/2021 | 483.60 | 1.23 | 0.00 | 0.005 |
| 9/28/2021 | 482.37 | 1.36 | 0.00 | 0.008 |
| 9/29/2021 | 481.01 | 0.36 | 0.00 | 0.008 |
| 9/30/2021 | 480.65 | 2.57 | 0.00 | 0.014 |
| 10/1/2021 | 478.08 | 1.89 | 0.00 | 0.018 |
| 10/5/2021 | 476.19 | 1.92 | 0.00 | 0.021 |
| 10/6/2021 | 474.27 | 0.65 | 0.00 | 0.023 |
| 10/7/2021 | 473.62 | 1.06 | 0.00 | 0.025 |
| 10/8/2021 | 472.57 | 1.60 | 0.00 | 0.028 |
| 10/9/2021 | 470.97 | 0.94 | 0.00 | 0.030 |
| 10/10/2021 | 470.03 | 4.12 | 0.00 | 0.039 |
| 10/11/2021 | 465.91 | 1.21 | 0.00 | 0.041 |
| 10/14/2021 | 464.70 | 0.70 | 0.00 | 0.043 |
| 10/15/2021 | 464.00 | 1.20 | 0.00 | 0.045 |
| 10/16/2021 | 462.80 | 0.61 | 0.00 | 0.046 |
| 10/18/2021 | 462.19 | 1.16 | 0.00 | 0.049 |
| 10/19/2021 | 461.03 | 2.35 | 0.00 | 0.054 |
| 10/20/2021 | 458.68 | 1.99 | 0.00 | 0.058 |
| 10/21/2021 | 456.70 | 0.41 | 0.00 | 0.059 |
| 10/22/2021 | 456.28 | 2.71 | 0.00 | 0.064 |
| 10/23/2021 | 453.57 | 2.22 | 0.00 | 0.069 |
| 10/24/2021 | 451.35 | 1.07 | 0.00 | 0.071 |
| 10/25/2021 | 450.29 | 0.49 | 0.00 | 0.072 |
| 10/27/2021 | 449.80 | 1.08 | 0.00 | 0.074 |
| 10/28/2021 | 448.72 | 2.27 | 0.00 | 0.079 |
| 10/29/2021 | 446.44 | 1.62 | 0.00 | 0.082 |
| 10/30/2021 | 444.82 | 1.74 | 0.00 | 0.086 |
| 11/1/2021 | 443.08 | 4.13 | 0.00 | 0.094 |
| 11/2/2021 | 438.95 | 0.85 | 0.00 | 0.096 |
| 11/3/2021 | 438.10 | 2.29 | 0.00 | 0.101 |
| 11/4/2021 | 435.81 | 0.74 | 0.00 | 0.102 |
| 11/5/2021 | 435.08 | 2.58 | 0.00 | 0.108 |
| 11/6/2021 | 432.49 | 1.19 | 0.00 | 0.110 |
| 11/7/2021 | 431.30 | 0.29 | 0.00 | 0.111 |
| 11/8/2021 | 431.01 | 3.43 | 0.00 | 0.118 |
| 11/9/2021 | 427.58 | 2.29 | 0.00 | 0.123 |
| 11/10/2021 | 425.29 | 4.25 | 0.00 | 0.131 |
| 11/11/2021 | 421.04 | 1.35 | 0.00 | 0.134 |
| 11/12/2021 | 419.68 | 5.12 | 0.00 | 0.145 |
| 11/14/2021 | 414.56 | 0.37 | 0.00 | 0.145 |
| 11/15/2021 | 414.20 | 7.89 | 0.00 | 0.162 |
| 11/16/2021 | 406.31 | 1.11 | 0.00 | 0.164 |
| 11/17/2021 | 405.19 | 0.89 | 0.00 | 0.166 |
| 11/18/2021 | 404.31 | 2.98 | 0.00 | 0.172 |
| 11/19/2021 | 401.33 | 3.01 | 0.00 | 0.178 |
| 11/20/2021 | 398.32 | 4.00 | 0.00 | 0.186 |
| 11/21/2021 | 394.32 | 0.52 | 0.00 | 0.188 |
| 11/22/2021 | 393.80 | 2.18 | 0.00 | 0.192 |
| 11/23/2021 | 391.62 | 3.11 | 0.00 | 0.198 |
| 11/24/2021 | 388.51 | 2.19 | 0.00 | 0.203 |
| 11/25/2021 | 386.33 | 2.93 | 0.00 | 0.209 |
| 11/26/2021 | 383.40 | 1.26 | 0.00 | 0.212 |
| 11/27/2021 | 382.14 | 0.85 | 0.00 | 0.213 |
| 11/28/2021 | 381.28 | 1.07 | 0.00 | 0.216 |
| 11/29/2021 | 380.22 | 0.69 | 0.00 | 0.217 |
| 11/30/2021 | 379.52 | 7.18 | 0.00 | 0.232 |
| 12/1/2021 | 372.34 | 1.63 | 0.00 | 0.235 |
| 12/2/2021 | 370.71 | 5.14 | 0.00 | 0.246 |
| 12/3/2021 | 365.57 | 2.33 | 0.00 | 0.251 |
| 12/4/2021 | 363.24 | 1.72 | 0.00 | 0.254 |
| 12/5/2021 | 361.52 | 0.90 | 0.00 | 0.256 |
| 12/6/2021 | 360.62 | 1.88 | 0.00 | 0.260 |
| 12/7/2021 | 358.74 | 1.76 | 0.00 | 0.264 |
| 12/8/2021 | 356.97 | 2.90 | 0.00 | 0.269 |
| 12/9/2021 | 354.07 | 8.37 | 0.00 | 0.287 |
| 12/10/2021 | 345.70 | 9.56 | 0.00 | 0.306 |
| 12/11/2021 | 336.14 | 0.25 | 0.00 | 0.307 |
| 12/14/2021 | 335.89 | 2.58 | 0.00 | 0.312 |
| 12/15/2021 | 333.31 | 4.22 | 0.00 | 0.321 |
| 12/17/2021 | 329.09 | 1.78 | 0.00 | 0.325 |
| 12/18/2021 | 327.31 | 1.15 | 0.00 | 0.327 |
| 12/19/2021 | 326.16 | 0.74 | 0.00 | 0.329 |
| 12/20/2021 | 325.42 | 2.52 | 0.00 | 0.334 |
| 12/21/2021 | 322.90 | 1.67 | 0.00 | 0.337 |
| 12/22/2021 | 321.23 | 7.41 | 0.00 | 0.353 |
| 12/23/2021 | 313.82 | 0.29 | 0.00 | 0.353 |
| 12/24/2021 | 313.53 | 0.44 | 0.00 | 0.354 |
| 12/28/2021 | 313.09 | 1.58 | 0.00 | 0.357 |
| 12/29/2021 | 311.51 | 2.18 | 0.00 | 0.362 |
| 12/30/2021 | 309.33 | 2.05 | 0.00 | 0.366 |
| 1/1/2022 | 307.28 | 1.69 | 0.00 | 0.370 |
| 1/2/2022 | 305.59 | 0.95 | 0.00 | 0.371 |
| 1/3/2022 | 304.64 | 0.50 | 0.00 | 0.373 |
| 1/4/2022 | 304.14 | 3.22 | 0.00 | 0.379 |
| 1/5/2022 | 300.93 | 2.57 | 0.00 | 0.384 |
| 1/6/2022 | 298.35 | 3.74 | 0.00 | 0.392 |
| 1/7/2022 | 294.61 | 0.31 | 0.00 | 0.393 |
| 1/9/2022 | 294.31 | 0.91 | 0.00 | 0.395 |
| 1/10/2022 | 293.40 | 2.71 | 0.00 | 0.400 |
| 1/11/2022 | 290.70 | 1.50 | 0.00 | 0.403 |
| 1/12/2022 | 289.19 | 0.34 | 0.00 | 0.404 |
| 1/13/2022 | 288.86 | 1.63 | 0.00 | 0.407 |
| 1/14/2022 | 287.23 | 0.61 | 0.00 | 0.409 |
| 1/15/2022 | 286.62 | 1.03 | 0.00 | 0.411 |
| 1/19/2022 | 285.59 | 0.77 | 0.00 | 0.412 |
| 1/20/2022 | 284.82 | 0.75 | 0.00 | 0.414 |
| 1/21/2022 | 284.07 | 0.89 | 0.00 | 0.416 |
| 1/24/2022 | 283.18 | 0.61 | 0.00 | 0.417 |
| 1/25/2022 | 282.57 | 0.50 | 0.00 | 0.418 |
| 1/31/2022 | 282.07 | 0.46 | 0.00 | 0.419 |
| 2/1/2022 | 281.61 | 0.30 | 0.00 | 0.420 |
| 2/2/2022 | 281.32 | 0.46 | 0.00 | 0.421 |
| 2/5/2022 | 280.85 | 0.56 | 0.00 | 0.422 |
| 2/9/2022 | 280.29 | 0.62 | 0.00 | 0.423 |
| 2/11/2022 | 279.67 | 0.91 | 0.00 | 0.425 |
| 2/12/2022 | 278.76 | 1.63 | 0.00 | 0.428 |
| 2/14/2022 | 277.13 | 0.50 | 0.00 | 0.429 |
| 2/18/2022 | 276.64 | 0.93 | 0.00 | 0.431 |
| 3/4/2022 | 275.70 | 1.06 | 0.00 | 0.433 |
| 3/7/2022 | 274.64 | 2.77 | 0.00 | 0.439 |
| 3/18/2022 | 271.87 | 0.77 | 0.00 | 0.441 |
| 4/1/2022 | 271.09 | 3.28 | 0.00 | 0.447 |
| 4/2/2022 | 267.81 | 0.40 | 0.00 | 0.448 |
| 4/4/2022 | 267.41 | 0.40 | 0.00 | 0.449 |
| 4/7/2022 | 267.01 | 0.69 | 0.00 | 0.451 |
| 4/11/2022 | 266.32 | 1.52 | 0.00 | 0.454 |
| 4/16/2022 | 264.80 | 1.08 | 0.00 | 0.456 |
| 4/17/2022 | 263.73 | 3.00 | 0.00 | 0.462 |
| 4/20/2022 | 260.73 | 0.76 | 0.00 | 0.464 |
| 4/22/2022 | 259.97 | 1.20 | 0.00 | 0.466 |
| 4/27/2022 | 258.78 | 0.63 | 0.00 | 0.467 |
| 5/23/2022 | 258.15 | 0.50 | 0.00 | 0.468 |
| 5/26/2022 | 257.65 | 0.00 | 19.29 | 0.468 |
| 5/27/2022 | 238.36 | 0.00 | 57.09 | 0.468 |
| 5/28/2022 | 181.26 | 0.00 | 61.24 | 0.468 |
| 5/29/2022 | 120.03 | 0.00 | 8.93 | 0.468 |
| 5/30/2022 | 111.10 | 0.00 | 17.93 | 0.468 |
| 5/31/2022 | 93.16 | 0.00 | 20.40 | 0.468 |
| 6/1/2022 | 72.76 | 0.00 | 4.99 | 0.468 |
| 6/2/2022 | 67.77 | 0.00 | 20.69 | 0.468 |
| 6/3/2022 | 47.08 | 0.00 | 2.49 | 0.468 |
| 6/4/2022 | 44.59 | 0.00 | 4.98 | 0.468 |
| 6/6/2022 | 39.60 | 0.00 | 2.10 | 0.468 |
| 6/7/2022 | 37.51 | 0.00 | 6.16 | 0.468 |
| 6/8/2022 | 31.34 | 0.00 | 2.91 | 0.468 |
| 6/10/2022 | 28.43 | 0.00 | 1.70 | 0.468 |
| 6/12/2022 | 26.73 | 0.00 | 3.69 | 0.468 |
| 6/14/2022 | 23.04 | 0.00 | 1.54 | 0.468 |
| 6/16/2022 | 21.49 | 0.00 | 1.57 | 0.468 |
| 6/17/2022 | 19.92 | 0.00 | 6.23 | 0.468 |
| 6/18/2022 | 13.69 | 0.00 | 5.86 | 0.468 |
| 6/21/2022 | 7.84 | 0.00 | 2.78 | 0.468 |
| 6/22/2022 | 5.06 | 0.00 | 0.42 | 0.468 |
| 6/24/2022 | 4.64 | 0.00 | 0.41 | 0.468 |
| 6/28/2022 | 4.23 | 0.00 | 3.20 | 0.468 |
| 6/29/2022 | 1.03 | 0.00 | 1.03 | 0.468 |

| *Table S31. Ready Confidents Booster Uptake Survival Table* | | | | |
| --- | --- | --- | --- | --- |
| Time | At risk | Fail | Net lost | Failure function |
| 8/1/2021 | 0.00 | 0.00 | -699.45 | 0.000 |
| 8/7/2021 | 699.45 | 0.51 | 0.00 | 0.001 |
| 8/10/2021 | 698.95 | 0.74 | 0.00 | 0.002 |
| 8/12/2021 | 698.21 | 0.40 | 0.00 | 0.002 |
| 8/15/2021 | 697.81 | 0.77 | 0.00 | 0.003 |
| 8/16/2021 | 697.05 | 0.48 | 0.00 | 0.004 |
| 8/18/2021 | 696.57 | 0.45 | 0.00 | 0.005 |
| 8/19/2021 | 696.12 | 2.80 | 0.00 | 0.009 |
| 8/20/2021 | 693.32 | 1.05 | 0.00 | 0.010 |
| 8/21/2021 | 692.27 | 1.22 | 0.00 | 0.012 |
| 8/23/2021 | 691.05 | 0.47 | 0.00 | 0.013 |
| 8/30/2021 | 690.58 | 2.27 | 0.00 | 0.016 |
| 8/31/2021 | 688.31 | 0.95 | 0.00 | 0.017 |
| 9/1/2021 | 687.36 | 0.40 | 0.00 | 0.018 |
| 9/2/2021 | 686.95 | 1.60 | 0.00 | 0.020 |
| 9/9/2021 | 685.35 | 0.64 | 0.00 | 0.021 |
| 9/11/2021 | 684.71 | 0.44 | 0.00 | 0.022 |
| 9/12/2021 | 684.27 | 0.27 | 0.00 | 0.022 |
| 9/13/2021 | 684.01 | 0.69 | 0.00 | 0.023 |
| 9/14/2021 | 683.31 | 0.66 | 0.00 | 0.024 |
| 9/17/2021 | 682.65 | 0.45 | 0.00 | 0.025 |
| 9/18/2021 | 682.20 | 0.55 | 0.00 | 0.025 |
| 9/21/2021 | 681.65 | 0.61 | 0.00 | 0.026 |
| 9/23/2021 | 681.04 | 2.33 | 0.00 | 0.030 |
| 9/24/2021 | 678.71 | 2.57 | 0.00 | 0.033 |
| 9/25/2021 | 676.14 | 4.37 | 0.00 | 0.040 |
| 9/26/2021 | 671.77 | 2.32 | 0.00 | 0.043 |
| 9/27/2021 | 669.46 | 4.98 | 0.00 | 0.050 |
| 9/28/2021 | 664.47 | 3.39 | 0.00 | 0.055 |
| 9/29/2021 | 661.09 | 3.68 | 0.00 | 0.060 |
| 9/30/2021 | 657.40 | 4.07 | 0.00 | 0.066 |
| 10/1/2021 | 653.34 | 7.03 | 0.00 | 0.076 |
| 10/2/2021 | 646.31 | 1.79 | 0.00 | 0.079 |
| 10/3/2021 | 644.52 | 2.09 | 0.00 | 0.082 |
| 10/4/2021 | 642.43 | 1.95 | 0.00 | 0.084 |
| 10/5/2021 | 640.48 | 3.65 | 0.00 | 0.090 |
| 10/6/2021 | 636.84 | 4.92 | 0.00 | 0.097 |
| 10/7/2021 | 631.92 | 2.99 | 0.00 | 0.101 |
| 10/8/2021 | 628.93 | 8.01 | 0.00 | 0.112 |
| 10/9/2021 | 620.92 | 3.94 | 0.00 | 0.118 |
| 10/10/2021 | 616.98 | 6.32 | 0.00 | 0.127 |
| 10/11/2021 | 610.65 | 4.57 | 0.00 | 0.133 |
| 10/12/2021 | 606.08 | 5.01 | 0.00 | 0.141 |
| 10/13/2021 | 601.07 | 1.46 | 0.00 | 0.143 |
| 10/14/2021 | 599.61 | 4.35 | 0.00 | 0.149 |
| 10/15/2021 | 595.26 | 11.58 | 0.00 | 0.166 |
| 10/16/2021 | 583.69 | 0.85 | 0.00 | 0.167 |
| 10/17/2021 | 582.84 | 0.33 | 0.00 | 0.167 |
| 10/18/2021 | 582.52 | 2.09 | 0.00 | 0.170 |
| 10/19/2021 | 580.43 | 5.64 | 0.00 | 0.178 |
| 10/20/2021 | 574.79 | 2.90 | 0.00 | 0.182 |
| 10/21/2021 | 571.89 | 4.64 | 0.00 | 0.189 |
| 10/22/2021 | 567.25 | 2.94 | 0.00 | 0.193 |
| 10/23/2021 | 564.31 | 2.27 | 0.00 | 0.196 |
| 10/24/2021 | 562.04 | 1.17 | 0.00 | 0.198 |
| 10/25/2021 | 560.87 | 5.49 | 0.00 | 0.206 |
| 10/26/2021 | 555.38 | 8.57 | 0.00 | 0.218 |
| 10/27/2021 | 546.81 | 6.88 | 0.00 | 0.228 |
| 10/28/2021 | 539.93 | 10.86 | 0.00 | 0.244 |
| 10/29/2021 | 529.07 | 6.54 | 0.00 | 0.253 |
| 10/30/2021 | 522.53 | 4.62 | 0.00 | 0.260 |
| 10/31/2021 | 517.91 | 2.32 | 0.00 | 0.263 |
| 11/1/2021 | 515.59 | 6.96 | 0.00 | 0.273 |
| 11/2/2021 | 508.63 | 5.99 | 0.00 | 0.281 |
| 11/3/2021 | 502.64 | 3.78 | 0.00 | 0.287 |
| 11/4/2021 | 498.86 | 7.47 | 0.00 | 0.297 |
| 11/5/2021 | 491.39 | 8.50 | 0.00 | 0.310 |
| 11/6/2021 | 482.89 | 11.61 | 0.00 | 0.326 |
| 11/7/2021 | 471.28 | 0.41 | 0.00 | 0.327 |
| 11/8/2021 | 470.86 | 6.28 | 0.00 | 0.336 |
| 11/9/2021 | 464.58 | 9.47 | 0.00 | 0.349 |
| 11/10/2021 | 455.12 | 8.14 | 0.00 | 0.361 |
| 11/11/2021 | 446.98 | 6.47 | 0.00 | 0.370 |
| 11/12/2021 | 440.51 | 10.40 | 0.00 | 0.385 |
| 11/13/2021 | 430.11 | 4.67 | 0.00 | 0.392 |
| 11/14/2021 | 425.44 | 5.99 | 0.00 | 0.400 |
| 11/15/2021 | 419.45 | 14.98 | 0.00 | 0.422 |
| 11/16/2021 | 404.47 | 4.05 | 0.00 | 0.428 |
| 11/17/2021 | 400.42 | 5.85 | 0.00 | 0.436 |
| 11/18/2021 | 394.57 | 2.99 | 0.00 | 0.440 |
| 11/19/2021 | 391.58 | 6.48 | 0.00 | 0.449 |
| 11/20/2021 | 385.09 | 7.67 | 0.00 | 0.460 |
| 11/21/2021 | 377.43 | 4.24 | 0.00 | 0.466 |
| 11/22/2021 | 373.19 | 6.87 | 0.00 | 0.476 |
| 11/23/2021 | 366.32 | 3.04 | 0.00 | 0.481 |
| 11/24/2021 | 363.28 | 2.38 | 0.00 | 0.484 |
| 11/25/2021 | 360.90 | 1.58 | 0.00 | 0.486 |
| 11/26/2021 | 359.32 | 3.66 | 0.00 | 0.492 |
| 11/27/2021 | 355.66 | 3.08 | 0.00 | 0.496 |
| 11/28/2021 | 352.58 | 2.72 | 0.00 | 0.500 |
| 11/29/2021 | 349.87 | 5.77 | 0.00 | 0.508 |
| 11/30/2021 | 344.09 | 3.14 | 0.00 | 0.513 |
| 12/1/2021 | 340.95 | 11.87 | 0.00 | 0.530 |
| 12/2/2021 | 329.08 | 4.48 | 0.00 | 0.536 |
| 12/3/2021 | 324.60 | 7.67 | 0.00 | 0.547 |
| 12/4/2021 | 316.93 | 3.31 | 0.00 | 0.552 |
| 12/5/2021 | 313.63 | 3.17 | 0.00 | 0.556 |
| 12/6/2021 | 310.46 | 2.72 | 0.00 | 0.560 |
| 12/7/2021 | 307.74 | 2.41 | 0.00 | 0.563 |
| 12/8/2021 | 305.32 | 6.85 | 0.00 | 0.573 |
| 12/9/2021 | 298.47 | 4.14 | 0.00 | 0.579 |
| 12/10/2021 | 294.33 | 4.42 | 0.00 | 0.586 |
| 12/11/2021 | 289.90 | 4.25 | 0.00 | 0.592 |
| 12/12/2021 | 285.65 | 4.85 | 0.00 | 0.599 |
| 12/13/2021 | 280.80 | 0.50 | 0.00 | 0.599 |
| 12/14/2021 | 280.30 | 0.29 | 0.00 | 0.600 |
| 12/15/2021 | 280.01 | 6.84 | 0.00 | 0.609 |
| 12/16/2021 | 273.17 | 2.28 | 0.00 | 0.613 |
| 12/17/2021 | 270.89 | 3.46 | 0.00 | 0.618 |
| 12/18/2021 | 267.43 | 1.88 | 0.00 | 0.620 |
| 12/19/2021 | 265.55 | 1.17 | 0.00 | 0.622 |
| 12/20/2021 | 264.38 | 3.83 | 0.00 | 0.627 |
| 12/21/2021 | 260.55 | 7.47 | 0.00 | 0.638 |
| 12/22/2021 | 253.08 | 0.84 | 0.00 | 0.639 |
| 12/26/2021 | 252.24 | 0.61 | 0.00 | 0.640 |
| 12/27/2021 | 251.63 | 1.26 | 0.00 | 0.642 |
| 12/28/2021 | 250.37 | 4.24 | 0.00 | 0.648 |
| 12/29/2021 | 246.13 | 2.16 | 0.00 | 0.651 |
| 12/30/2021 | 243.96 | 2.14 | 0.00 | 0.654 |
| 12/31/2021 | 241.82 | 1.28 | 0.00 | 0.656 |
| 1/1/2022 | 240.55 | 2.00 | 0.00 | 0.659 |
| 1/2/2022 | 238.54 | 2.19 | 0.00 | 0.662 |
| 1/3/2022 | 236.36 | 2.92 | 0.00 | 0.666 |
| 1/5/2022 | 233.44 | 0.82 | 0.00 | 0.667 |
| 1/6/2022 | 232.62 | 1.72 | 0.00 | 0.670 |
| 1/7/2022 | 230.90 | 2.20 | 0.00 | 0.673 |
| 1/8/2022 | 228.70 | 0.50 | 0.00 | 0.674 |
| 1/10/2022 | 228.20 | 12.37 | 0.00 | 0.691 |
| 1/11/2022 | 215.83 | 1.48 | 0.00 | 0.694 |
| 1/12/2022 | 214.35 | 1.85 | 0.00 | 0.696 |
| 1/13/2022 | 212.50 | 0.86 | 0.00 | 0.697 |
| 1/14/2022 | 211.64 | 1.95 | 0.00 | 0.700 |
| 1/15/2022 | 209.70 | 0.62 | 0.00 | 0.701 |
| 1/17/2022 | 209.08 | 1.36 | 0.00 | 0.703 |
| 1/18/2022 | 207.72 | 0.40 | 0.00 | 0.704 |
| 1/20/2022 | 207.32 | 2.17 | 0.00 | 0.707 |
| 1/21/2022 | 205.14 | 1.57 | 0.00 | 0.709 |
| 1/23/2022 | 203.57 | 0.72 | 0.00 | 0.710 |
| 1/26/2022 | 202.85 | 2.99 | 0.00 | 0.714 |
| 1/29/2022 | 199.86 | 4.14 | 0.00 | 0.720 |
| 2/2/2022 | 195.73 | 2.02 | 0.00 | 0.723 |
| 2/3/2022 | 193.70 | 2.51 | 0.00 | 0.727 |
| 2/5/2022 | 191.19 | 0.84 | 0.00 | 0.728 |
| 2/7/2022 | 190.35 | 2.48 | 0.00 | 0.731 |
| 2/10/2022 | 187.87 | 1.32 | 0.00 | 0.733 |
| 2/13/2022 | 186.55 | 0.28 | 0.00 | 0.734 |
| 2/15/2022 | 186.27 | 1.93 | 0.00 | 0.736 |
| 2/19/2022 | 184.34 | 0.38 | 0.00 | 0.737 |
| 2/26/2022 | 183.96 | 0.46 | 0.00 | 0.738 |
| 3/1/2022 | 183.50 | 1.05 | 0.00 | 0.739 |
| 3/4/2022 | 182.45 | 3.69 | 0.00 | 0.744 |
| 3/7/2022 | 178.76 | 2.59 | 0.00 | 0.748 |
| 3/10/2022 | 176.16 | 0.74 | 0.00 | 0.749 |
| 3/20/2022 | 175.43 | 0.72 | 0.00 | 0.750 |
| 3/22/2022 | 174.71 | 0.89 | 0.00 | 0.751 |
| 4/1/2022 | 173.82 | 1.41 | 0.00 | 0.754 |
| 4/8/2022 | 172.41 | 1.03 | 0.00 | 0.755 |
| 4/14/2022 | 171.39 | 0.72 | 0.00 | 0.756 |
| 4/20/2022 | 170.67 | 0.25 | 0.00 | 0.756 |
| 4/26/2022 | 170.42 | 2.20 | 0.00 | 0.759 |
| 5/10/2022 | 168.22 | 0.69 | 0.00 | 0.760 |
| 5/16/2022 | 167.53 | 1.36 | 0.00 | 0.762 |
| 5/26/2022 | 166.17 | 0.00 | 3.92 | 0.762 |
| 5/27/2022 | 162.24 | 0.00 | 37.40 | 0.762 |
| 5/28/2022 | 124.85 | 0.88 | 35.40 | 0.764 |
| 5/29/2022 | 88.57 | 0.00 | 11.22 | 0.764 |
| 5/30/2022 | 77.35 | 0.00 | 11.07 | 0.764 |
| 5/31/2022 | 66.27 | 0.00 | 17.18 | 0.764 |
| 6/1/2022 | 49.09 | 0.00 | 9.52 | 0.764 |
| 6/2/2022 | 39.57 | 0.00 | 0.78 | 0.764 |
| 6/3/2022 | 38.79 | 0.00 | 0.65 | 0.764 |
| 6/4/2022 | 38.14 | 0.00 | 7.94 | 0.764 |
| 6/5/2022 | 30.21 | 0.00 | 2.39 | 0.764 |
| 6/6/2022 | 27.82 | 0.00 | 0.61 | 0.764 |
| 6/7/2022 | 27.21 | 0.00 | 7.46 | 0.764 |
| 6/8/2022 | 19.75 | 0.00 | 1.32 | 0.764 |
| 6/9/2022 | 18.43 | 0.00 | 2.52 | 0.764 |
| 6/13/2022 | 15.90 | 0.00 | 1.84 | 0.764 |
| 6/15/2022 | 14.07 | 0.00 | 0.22 | 0.764 |
| 6/17/2022 | 13.84 | 0.00 | 0.75 | 0.764 |
| 6/18/2022 | 13.09 | 0.00 | 0.63 | 0.764 |
| 6/19/2022 | 12.47 | 0.00 | 1.80 | 0.764 |
| 6/20/2022 | 10.67 | 0.00 | 1.81 | 0.764 |
| 6/23/2022 | 8.86 | 0.00 | 1.02 | 0.764 |
| 6/24/2022 | 7.84 | 0.00 | 0.80 | 0.764 |
| 6/30/2022 | 7.04 | 0.00 | 6.31 | 0.764 |
| 7/5/2022 | 0.73 | 0.00 | 0.73 | 0.764 |

**References**

15. NORC. Technical overview of the AmeriSpeak panel NORC’s probability-based household panel. (2022).

16. Pogue, K. et al. Influences on attitudes regarding potential COVID-19 vaccination in the United States. Vaccines 8, 582 (2020).

17. Reiter, P. L., Pennell, M. L. & Katz, M. L. Acceptability of a COVID-19 vaccine among adults in the United States: How many people would get vaccinated? Vaccine 38, 6500–6507 (2020).

18. Hornik, R. et al. Association of COVID-19 misinformation with face mask wearing and social distancing in a nationally representative US sample. Health Commun. 36, 6–14 (2021).

19. Alattar, L., Messel, M. & Rogofsky, D. An introduction to the understanding America study Internet panel. Soc Sec Bull 78, 13 (2018).

20. Kwok, K. O. et al. Influenza vaccine uptake, COVID-19 vaccination intention and vaccine hesitancy among nurses: A survey. Int. J. Nurs. Stud. 114, 103854 (2021).

21. Howard, M. C. Understanding face mask use to prevent coronavirus and other illnesses: Development of a multidimensional face mask perceptions scale. Br. J. Health Psychol. 25, 912–924 (2020).

22. Knotek II, E. et al. Consumers and COVID-19: survey results on mask-wearing behaviors and beliefs. Econ. Comment. (2020).

23. So, J. & Popova, L. A profile of individuals with anti-tobacco message fatigue. Am. J. Health Behav. 42, 109–118 (2018).

24. Nadelson, L. et al. I just don’t trust them: the development and validation of an assessment instrument to measure trust in science and scientists. Sch. Sci. Math. 114, 76–86 (2014).

25. Materu, J. et al. The psychometric properties of PHQ-4 anxiety and depression screening scale among out of school adolescent girls and young women in Tanzania: a cross-sectional study. BMC Psychiatry 20, 1–8 (2020).

26. Ahorsu, D. K. et al. The fear of COVID-19 scale: development and initial validation. Int. J. Ment. Health Addict. 1–9 (2020).

27. USDA. USDA ERS - Rural-Urban Continuum Codes. https://www.ers.usda.gov/data-products/rural-urban-continuum-codes.aspx (2023).

28. FDA. FDA Approves First COVID-19 Vaccine. FDA.Gov https://www.fda.gov/news-events/press-announcements/fda-approves-first-covid-19-vaccine (2021).

29. Muller, H.-G. & Wang, J.-L. Hazard rate estimation under random censoring with varying kernels and bandwidths. Biometrics 61–76 (1994).
